# Candidate Genes Involved in Beneficial or Adverse Responses to Commonly Eaten Brassica Vegetables in a New Zealand Crohn’s Disease Cohort

**DOI:** 10.3390/nu5125046

**Published:** 2013-12-12

**Authors:** Bobbi Laing, Dug Yeo Han, Lynnette R. Ferguson

**Affiliations:** Discipline of Nutrition, School of Medical Sciences, Auckland University, 85 Park Road, Grafton Campus, Auckland 1142, New Zealand; E-Mails: dy.han@auckland.ac.nz (D.Y.H.); l.ferguson@auckland.ac.nz (L.R.F.)

**Keywords:** Brassicaceae, tolerability, Crohn’s disease, *DIO1*, *HLA*

## Abstract

Crohn’s disease (CD) is one of the two manifestations of inflammatory bowel disease. Particular foods are thought with CD to exacerbate their illness. Vegetables, especially Brassicaceae, are often shunned by people with CD because of the negative effects they are alleged to have on their symptoms. Brassicaceae supply key nutrients which are necessary to meet recommended daily intakes. We sought to identify the candidate genes involved in the beneficial or adverse effects of Brassicaceae most commonly eaten, as reported by the New Zealand adults from the “Genes and Diet in Inflammatory Bowel disease Study” based in Auckland. An analysis of associations between the single nucleotide polymorphisms (SNPs) and the beneficial or adverse effects of the ten most commonly eaten Brassicaceae was carried out. A total of 37 SNPs were significantly associated with beneficial effects (*p* = 0.00097 to 0.0497) and 64 SNPs were identified with adverse effects (*p* = 0.0000751 to 0.049). After correcting for multiple testing, rs7515322 (*DIO1*) and rs9469220 (*HLA*) remained significant. Our findings show that the tolerance of some varieties of Brassicaceae may be shown by analysis of a person’s genotype.

## 1. Introduction

Crohn’s disease (CD) is one of two commonly identified inflammatory bowel diseases (IBD) the other being ulcerative colitis (UC). Both disorders are inflammatory and people experience phases of remission and deterioration.

The incidence of CD seems to be increasing and it appears that New Zealand (NZ) in 2006 had the highest rate at 16.5/10^5^ [1]. Increases are observed particularly in the western world, and research has shown that the environment plays a considerable role [2]. It has been observed that individuals from Bangladesh (where the incidence of IBD is very low) on shifting to the United Kingdom, develop a very high incidence of IBD within a generation [3]. Nutrition is thought to play a key role and is considered to be an important environmental factor influencing the development of CD and its symptoms [4], through the nutrigenomic and epigenetic modification of the susceptibility genes [5].

Vegetables, especially Brassicaceae, are often shunned by people with CD because of the negative effects they are alleged to have on their symptoms [6,7]. However, Brassicaceae supply key nutrients. Some varieties of Brassicaceae also appear to be well tolerated by people with CD [8]. Several studies show that Brassicaceae contain many significant nutrients: fibre, the antioxidants vitamin A and C, the vitamin folate and vitamin K and the minerals such as potassium, calcium, selenium and zinc as well as the numerous phytochemicals which have key roles in maintaining health [9,10,11,12]. They help improve immunity as well as contributing to anti-inflammatory and anti-cancer activities of the body [13,14,15,16,17,18,19,20,21]. The intake of sufficient amounts of these nutrients is important for people with CD. These nutrients are necessary to meet the daily intakes as recommended in the Nutrient Reference Values for Australia and New Zealand [22].

By studying the interaction of different Brassicaceae varieties with single nucleotide polymorphisms (SNPs) in people with CD, it may be possible to uncover a genetic basis for individual tolerances. This could lead to more specific nutrition advice with respect to these important vegetables, and enhance the opportunities for CD patients to avail themselves of the benefits of Brassicaceae. The aim of this study was therefore to identify the candidate genes involved in the beneficial or adverse effects of the Brassicaceae most commonly eaten as reported by NZ adults from the “Genes and Diet in IBD Study” [4], based in Auckland. 

## 2. Materials and Methods

### 2.1. Brassicaceae Selection

The Brassicaceae analysed for tolerability were those reported to be consumed by the subjects in the “Genes and Diet in IBD Study” based in Auckland NZ. These Brassicaceae were: broccoli, cabbage, cauliflower, Chinese greens, rocket (arugula), watercress, horseradish, mustard sauce, mustard powder and wasabi.

### 2.2. Tolerability of Brassicaceae

The tolerability of the Brassicaceae was ascertained from secondary analysis of the information gained from the responses to the dietary questionnaire. This questionnaire was based on Joachim’s methodology [23] and feedback from a group of patients with CD [4]. Questions in the survey were asked (and scored on a five-point scale on whether the person’s IBD condition became either: definitely better, (++) probably better (+), had no effect (=), probably worse (−), and definitely worse (− − in response to the listed food. This allowed information to be collected on how food affected their disease symptoms. This takes into account that many foods are never consumed because they are not considered palatable irrespective of the effects they are perceived to have on their symptoms of CD.

Two scales were used: the percentage of beneficial effects (definitely better and probably better) and adverse effects (definitely worse and probably worse). The responses to “makes no difference” were omitted. A nutritionist or registered nurse checked out the responses with respondents when there were any queries [4]. The dietary questionnaire also included open ended questions. These were also evaluated for additional information on foods which may affect symptoms associated with CD.

### 2.3. Study Population

Study participants were from the main North Island centre, Auckland and other major North Island centres. They were enrolled as part of a population based study in the IBD project whose purpose was to determine the genetic and environmental factors of CD aetiology. These people were enlisted from gastroenterology clinics or by their response from advertising in the media between May 2005 and April 2009. A total of 339 Caucasian subjects gave their informed consent to take part. The nutritional questionnaire was completed by 290 and up to 323 were genotyped (Table 1). Consent was obtained before the collection of peripheral blood for DNA extraction and genotyping. The questionnaires were taken away for participants to complete and on their return scrutinised for accuracy and completion and where necessary subjects were contacted again for clarification. All of the participants were selected on the basis of self-disclosed Caucasian ancestry only [4]. Clinical data (age, IBD diagnosis and the most recent Montreal classification of CD location [24]) were extracted from the clinical questionnaire and patient medical notes, which were supplied by the diagnosing gastroenterologist in the primary “Genes and Diet in IBD Study”. Ethical approval was given by the NZ Multi-region Human Ethics Committee (MEC04/12/11).

### 2.4. Genotyping

SNP data with respect to the beneficial or adverse effects of selected Brassicaceae were taken from the original “Genes and Diet in IBD Study” based in Auckland, NZ. Gene polymorphisms in this study were determined by polymerase chain reaction-restriction fragment length polymorphism (PCR-RFLP) analysis. The SNPs identified for each Brassicaceae variety were corrected for multiple testing using a false discovery rate [25]. R was used for statistical analyses. A *p*-value was considered significant if it was less than 0.05 [26].

**Table 1:** Summary of sample sizes and clinical data for those with Crohn’s disease (CD).

Sub-phenotypes	Phenotype Description	*N* (%)
Age at diagnosis	<17	31 (11.0)
	17–40	205 (72.7)
	>40	46 (16.3)
Behaviour	Inflammatory	158 (56.0)
	Stricturing	91 (32.3)
	Penetrating	33 (11.7)
Location	Ileal	111 (39.4)
	Colonic	92 (32.6)
	Ileocolon	79 (28.0)
Bowel Resection	No	180 (63.8)
	Yes	102 (36.2)
Extra-intestinal manifestations	No	232 (82.3)
	Yes	50 (17.7)

Note: A total of 339 participants were available for genotyping and up to 323 were genotyped. Of those, clinical information was available for 282 and the nutrition questionnaire was completed by 290; *N*—sample size.

## 3. Results

Table 2 shows the candidate genes. Table 3 shows the SNPs that were significantly associated with beneficial effects while Table 4 shows the significant SNPs which were associated with adverse effects on the symptoms of people with CD. Table S1 shows the Brassicacea frequencies [*n*(%)] by SNPS. A total of 37 variants were identified as having beneficial effects (*p* values from 0.00097 to 0.0497) and 64 SNPs with adverse effects (0.0000751 to 0.049). After correcting for multiple testing, two SNPs in two genes remained significant with adverse reactions to Brassicaceae. The rs7515322 variant in *DIO1* was associated with Broccoli, and the rs9469220 variant in *HLA* was associated with rocket. These *p*-values were marked with an asterisk (*) (Table 4).

**Table 2:** CD related single nucleotide polymorphisms (SNPs) for association with Brassicaceae.

Gene	Name	SNP	Chr
*DIO1*	deiodinase, iodothyronine, type I	rs11206244	1
		rs7515322	1
*IL23R*	interleukin 23 receptor	rs11209026	1
	interleukin 23 receptor	rs7517847	1
*ITLN1*	intelectin (galactofuranose binding)	rs2274910	1
*SEP15*	15 kDa selenoprotein	rs5845	1
	15 kDa selenoprotein	rs5859	1
*TNFRSF1B*	tumor necrosis factor receptor superfamily, member 1B	rs3397	1
*TNFSF15*	tumor necrosis factor (ligand) superfamily, member 15	rs7029554	1
*ATG16L1*	ATG16 autophagy related 16-like 1	rs10210302	2
*SLC11A1*	solute carrier family 11 (proton-coupled divalent metal ion transporters), member 1	rs3731865	2
*AMT*	aminomethyltransferase	rs11922013	3
	Aminomethyltransferase	rs1464567	3
*BSN*	bassoon presynaptic cytomatrix	rs2131109	3
	bassoon presynaptic cytomatrix	rs4283605	3
*CDKAL1*	CDK5 regulatory subunit	rs6908425	3
*FHIT*	fragile histidine triad gene	rs2250114	3
*GPX1*	glutathione peroxidase 1	rs1800668	3
*SLC6A6*	solute carrier family 6 (neurotransmitter transporter, taurine), member 6	rs41284011	3
	solute carrier family 6 (neurotransmitter transporter, taurine), member 6	rs4685154	3
*TF*	transferrin	rs1799899	3
*TLR9*	toll-like receptor 9	rs5743836	3
*TRAIP*	TRAF interacting protein	rs10865959	3
	TRAF interacting protein	rs17598137	3
	TRAF interacting protein	rs2271960	3
	TRAF interacting protein	rs6446298	3
*USP4*	ubiquitin specific peptidase 4 (proto-oncogene)	rs1865741	3
	ubiquitin specific peptidase 4 (proto-oncogene)	rs9881860	3
*CDH29*	cadherin-related family member 4	rs7629936	4
*ATG12*	*ATG12 autophagy related 12 homolog (S. cerevisiae)*	rs26532	5
*CSF1R*	colony stimulating factor 1 receptor	rs2282804	5
*desert_PTGER4*	desert Prostaglandin E receptor 4 (subtype EP4)	rs17234657	5
*desert_PTGER4*	desert Prostaglandin E receptor 4 (subtype EP4)	rs9292777	5
*GPX3*	glutathione perosidase 3 (plasma)	rs2042235	5
	glutathione perosidase 3 (plasma)	rs3763013	5
	glutathione perosidase 3 (plasma)	rs3792796	5
	glutathione perosidase 3 (plasma)	rs3792797	5
	glutathione perosidase 3 (plasma)	rs3805435	5
	glutathione perosidase 3 (plasma)	rs3828599	5
	glutathione perosidase 3 (plasma)	rs8177425	5
	glutathione perosidase 3 (plasma)	rs870407	5
*IBD5*	inflammatory bowel disease	rs10077785	5
*IL12B*	interleukin 12B (natural killer cell stimulatory factor 2)	rs6887695	5
*IRGM*	immunity-related GTPase family,	rs4958847	5
*OCTN1/SLC22A4*	solute carrier family 22 (organic cation/ergothioneine transporter), member	rs1050152	5
*OCTN2/SLC22A5*	solute carrier family 22 (organic cation/carnitine transporter), member 5	rs2631367	5
*P4HA2*	prolyl 4-hydroxylase, alpha polypeptide II	rs4361509	5
*PTGER4*	prostaglandin E receptor 4 (subtype EP4)	rs1373692	5
	prostaglandin E receptor 4 (subtype EP4)	rs4613763	5
*SEPP1*	selenoprotein P, plasma, 1	rs3877899	5
*HLA*	major histocompatibility complex, class II, DO alpha	rs9469220	6
*TNFALPHA*	tumor necrosis factor	rs1800629	6
*CLDN12*	claudin 12	rs1017106	7
	claudin 12	rs17864006	7
*CNTNAP2*	contactin associated protein-like 2	rs7807268	7
*IL6*	interleukin 6 (interferon, beta 2)	rs1800795	7
*DEFA6*	defensin, alpha 6, Paneth cell-specific	rs712276	8
*JAK2*	janus kinase 2	rs10758669	9
*TNFSF15*	tumor necrosis factor (ligand) superfamily, member 15	rs10982412	9
	tumor necrosis factor (ligand) superfamily, member 15	rs3810936	9
	tumor necrosis factor (ligand) superfamily, member 15	rs7867918	9
*DLG5*	discs, large homolog 5 (Drosophila)	rs2289311	10
*NKX2-3*	NK2 homeobox 3	rs10883365	10
*JAM3*	junctional adhesion molecule 3	rs11604455	11
*SLC11A2*	solute carrier family 11 (proton-coupled divalent metal ion transporters), member 2	rs224589	12
	solute carrier family 11 (proton-coupled divalent metal ion transporters), member 3	rs427020	12
*VDR*	vitamin D (1,25- dihydroxyvitamin D3) receptor	rs7975232	12
*LAMP1*	lysosomal-associated membrane protein 1	rs12871648	13
*DIO2*	deiodinase, iodothyronine, type II	rs12885300	14
*DIO3*	deiodinase, iodothyronine, type III	rs1190715	14
	deiodinase, iodothyronine, type III	rs1190716	14
	deiodinase, iodothyronine, type III	rs945006	14
*GPX2*|*CHURC1-FNTB*	glutathione peroxidase 2, CHURC1-FNTB readthrough	rs1800669	14
	glutathione peroxidase 2, CHURC1-FNTB readthrough	rs2296327	14
	glutathione peroxidase 2, CHURC1-FNTB readthrough	rs2412065	14
	glutathione peroxidase 2, CHURC1-FNTB readthrough	rs2737844	14
	glutathione peroxidase 2, CHURC1-FNTB readthrough	rs3742599	14
*SELS*	selenoprotein S	rs4965814	15
	selenoprotein S	rs7178239	15
*FAM92B*	family with sequence similarity 92, member B	rs8050910	16
*MAP1LC3B*	microtubule-associated protein 1 light chain 3 beta	rs2288483	16
	microtubule-associated protein 1 light chain 3 beta	rs7204722	16
	microtubule-associated protein 1 light chain 3 beta	rs8044820	16
	microtubule-associated protein 1 light chain 3 beta	rs8051218	16
*NOD2*	nucleotide-binding oligomerization domain containing 2	rs2066844	16
*STAT3*	signal transducer and activator of transcription 3	rs744166	17
*TNRC6C*	trinucleotide repeat containing 6C	rs4362447	17
*PTPN2*	protein tyrosine phosphatase, non-receptor type 2	rs2542151	18
*ICAM1*	intercellular adhesion molecule 1	rs1799969	19
*TFF3*	trefoil factor 3 (intestinal)	rs225369	21
*MIF*	macrophage migration inhibitory factor	rs755622	22
*CLDN2*	claudin 2	rs12008279	X

**Table 3:** Results of SNPs from the candidate genes associated with beneficial effects of Brassicaceae; showing *p*-values only; significant *p* values highlighted in italic and bold; n/r: no response.

Gene	SNP	Cauliflower	Broccoli	Cabbage	Chinese Greens	rocket	Watercress	Mustard powder	Mustard Sauce	Wasabi
*AMT*	rs11922013	0.2451	0.7510	0.3506	0.5299	0.6430	***0.0418***	0.3177	***0.0172***	0.9995
*ATG12*	rs26532	0.0653	0.3809	0.3724	0.1532	***0.0128***	0.9788	***0.0237***	0.1822	0.0642
*CDH29*	rs7629936	0.7431	0.7083	***0.0454***	0.3585	0.9900	0.3356	0.1522	0.4627	0.8607
*CNTNAP2*	rs7807268	0.0899	0.4994	0.2787	***0.0226***	0.3516	0.1203	0.3510	0.1390	0.9995
*CSF1R*	rs2282804	***0.0139***	0.3442	0.4589	0.1351	0.6996	0.5417	0.9999	0.9441	0.9996
*DEFA6*	rs712276	***0.00097***	***0.0016***	***0.0048***	***0.0094***	***0.0302***	***0.0197***	***0.0310***	0.7953	0.7298
*desert_PTGER4*	rs9292777	n/r	n/r	n/r	***0.0296***	n/r	n/r	0.9772	0.2711	n/r
*FAM92B*	rs8050910	0.9996	***0.0410***	0.9995	0.1001	0.2675	0.9996	0.9999	0.9999	1.0000
*FHIT*	rs2250114	0.7337	0.8403	0.0590	0.9747	0.0508	0.7876	***0.0354***	***0.0226***	0.0507
*GPX3*	rs3763013	***0.0103***	***0.0169***	0.9996	0.2862	0.2754	0.2866	0.4011	0.6294	0.4772
*GPX3*	rs3792796	***0.0187***	***0.0280***	0.8501	0.3827	0.9010	0.6353	0.9240	0.2130	0.5985
*GPX3*	rs870407	0.9996	***0.0475***	0.7659	0.9368	0.7542	0.9859	0.8520	0.9999	0.9996
*ICAM1*	rs1799969	0.9168	0.9046	***0.0270***	0.8428	***0.0060***	***0.0143***	***0.0138***	0.2538	0.0635
*IL6*	rs1800795	0.9363	0.5219	0.6916	0.8112	***0.0046***	0.4276	0.0744	***0.0293***	0.9996
*IRGM*	rs4958847	0.1783	0.4637	0.2814	***0.0378***	0.5938	0.8985	0.8482	0.8803	0.4834
*ITLN1*	rs2274910	0.3727	***0.0374***	0.6161	0.4158	0.5626	0.8039	0.3690	0.6410	0.7704
*JAM3*	rs11604455	0.7261	***0.0350***	0.9996	0.8063	0.9997	0.9799	0.9999	0.9999	0.9997
*MAP1LC3B*	rs2288483	***0.0403***	***0.0111***	0.0590	0.2511	0.3290	0.5593	0.1842	0.9373	0.6128
*MAP1LC3B*	rs7204722	0.2386	***0.0361***	0.3033	0.3514	0.5558	0.8065	0.3161	0.7094	0.7709
*MAP1LC3B*	rs8044820	0.5899	0.8822	***0.0223***	0.5065	0.8666	0.4233	0.4110	0.9999	0.9997
*MIF*	rs755622	***0.0139***	***0.0047***	0.2191	0.3475	0.3667	0.5391	0.9999	0.2737	1.0000
*NKX2.3*	rs10883365	***0.0269***	0.1211	0.1447	***0.0157***	0.0552	***0.0225***	0.0964	0.1120	0.9195
*NOD2*	rs2066844	0.8928	0.6265	0.8517	0.9735	0.4115	0.9820	***0.0397***	0.9999	0.9996
*PTGER4*	rs1373692	0.1842	0.0890	0.9742	***0.0112***	0.4711	0.5627	0.9594	0.3113	0.4380
*PTPN2*	rs2542151	***0.0103***	***0.0019***	***0.0023***	***0.0286***	0.1077	***0.0090***	0.9999	0.9858	0.9168
*SEP_15*	rs5859	0.6706	0.1994	0.0535	0.4249	0.3915	0.7017	***0.0497***	0.5820	0.9369
*SEPP1*	rs3877899	***0.0045***	***0.0127***	0.7790	0.1128	0.7454	***0.0360***	0.7790	0.6607	0.5572
*SLC6A6*	rs41284011	n/r	n/r	***0.0094***	n/r	n/r	n/r	0.9999	0.9999	n/r
*SLC6A6*	rs4685154	0.5897	0.9635	0.6535	0.1191	0.1927	***0.0470***	0.2540	0.8530	0.4554
*TNFRSF1B*	rs3397	0.2835	0.0896	0.1402	0.1193	***0.0493***	0.5872	0.9999	0.9990	1.0000
*TNFSF15*	rs3810936	0.8782	0.5239	0.5199	0.1291	0.8847	***0.0457***	0.8960	0.2650	0.7494
*TNFSF15*	rs7867918	***0.0438***	0.0824	0.1652	0.0613	0.2248	0.0718	0.0729	0.1870	0.7609
*TNRC6C*	rs4362447	0.0636	0.1906	0.9391	***0.0285***	0.8595	0.0795	0.9970	0.5730	0.4840
*TRAIP*	rs17598137	0.9995	0.9997	0.9996	0.9996	0.9996	0.9996	0.9999	0.0951	***0.0248***
*TRAIP*	rs2271960	0.3267	***0.0354***	0.3621	0.3636	0.4224	0.8182	0.2940	0.1050	0.9996
*TRAIP*	rs6446298	***0.0424***	0.0556	0.6071	0.0989	0.1827	0.1409	0.9840	0.2270	0.9997
*USP4*	rs9881860	0.8590	0.3827	0.3214	0.8727	0.6817	0.9307	0.8077	***0.0449***	0.2229

**Table 4:** Results of SNPs from candidate genes associated with adverse effects of Brassicaceae; showing *p*-values only; significant *p* values highlighted in italic and bold. (* Remained significant after applying multiple testing correction using false discovery rate).

Gene	SNP	Broccoli	Cabbage	Cauliflower	Chinese Greens	Rocket	Watercress	Horseradish	Mustard powder	Mustard Sauce	Wasabi
AMT	rs1464567	0.0652	0.9368	0.0667	***0.0376***	0.2402	0.2171	0.9354	0.4783	0.8241	0.5862
ATG16L1	rs10210302	0.7140	***0.0232***	0.2158	0.0559	0.3950	0.3398	0.2528	0.4525	0.0537	0.3769
BSN	rs2131109	0.3418	0.5838	***0.0317***	0.6593	0.4865	0.4534	0.5293	0.5810	0.5959	0.6147
BSN	rs4283605	0.7291	0.1096	0.7757	0.8219	0.5448	0.6208	***0.0235***	0.9420	0.7979	0.4625
CDH29	rs7629936	0.1395	0.7087	***0.0494***	0.1481	0.2022	0.2251	0.5519	0.5841	0.9684	0.0803
CDKAL1	rs6908425	0.8750	0.6255	0.8891	0.8246	0.2999	***0.0298***	0.2657	0.7070	0.3470	0.5407
CLDN12	rs1017106	0.8662	***0.0291***	0.3861	0.9996	0.9996	0.9900	0.0534	0.3336	0.0812	0.0527
CLDN12	rs17864006	0.3710	0.1913	0.3723	***0.0312***	0.2893	0.8806	0.8001	0.5230	0.9520	0.8456
CLDN2	rs12008279	0.4042	0.8127	0.8816	0.1002	***0.0481***	0.8982	0.3240	0.8170	0.8822	0.5584
*desert_PTGER4*	rs17234657	0.3319	***0.0458***	0.6535	0.0672	0.8378	0.9958	0.0959	***0.0244***	0.6530	0.1830
DIO1	rs11206244	0.3296	0.0968	0.4987	0.2154	0.2448	0.5280	***0.0136***	0.3460	0.1520	0.4456
DIO1	rs7515322	0.000167 *	0.1070	0.0864	0.5197	0.2208	0.0512	0.6658	0.3440	0.6550	0.3161
DIO2	rs12885300	0.2305	0.7110	0.9809	0.2811	0.5710	0.5532	***0.0083***	0.0833	0.2000	0.1935
DIO3	rs1190715	***0.0252***	0.8963	0.6316	0.6761	0.9875	0.5535	0.2095	0.5380	0.5386	***0.0211***
DIO3	rs1190716	0.1362	0.1126	0.9806	0.3182	0.5579	***0.0361***	0.1060	0.2720	0.2530	***0.0039***
DIO3	rs945006	0.5967	0.2792	0.2923	0.7176	0.6002	0.9202	0.0791	0.2230	0.1980	***0.0019***
DLG5	rs2289311	0.3003	0.0830	0.8446	0.7854	0.8730	***0.0423***	0.2711	0.1390	0.7660	0.5258
FHIT	rs2250114	0.3770	0.4506	0.4490	***0.0066***	0.8620	0.4285	0.1317	0.9320	0.5110	***0.0191***
GPX1	rs1800668	0.5773	0.7500	0.7313	0.6076	0.2872	0.2612	***0.0418***	0.1677	0.4277	0.1434
GPX2	rs1800669	0.3562	***0.0374***	0.2652	0.3266	0.9201	0.9997	0.3097	0.1110	0.0830	0.7837
GPX2	rs2296327	0.6288	0.8722	0.6316	0.4766	0.1252	0.8774	0.3734	0.8050	0.8450	***0.0392***
GPX2	rs2412065	0.6747	0.8985	0.6886	0.7937	0.9786	0.7360	0.0807	***0.0377***	***0.0101***	***0.0164***
GPX2	rs2737844	0.5789	0.6613	0.2104	0.2119	0.2871	0.4791	0.2838	0.0637	0.1543	***0.0230***
GPX2	rs3742599	0.5281	0.7762	0.8361	0.5321	0.7983	0.7495	0.1121	0.1326	0.0673	***0.0276***
GPX3	rs2042235	0.3841	0.5588	0.0719	0.3311	***0.0019***	0.0958	0.4810	0.2850	0.5060	0.1288
GPX3	rs3792796	0.6028	0.5677	0.7284	0.7336	0.1833	0.4795	0.8515	***0.0202***	0.3904	0.7421
GPX3	rs3792797	0.8569	0.6572	0.9486	0.4927	***0.0135***	0.0546	0.8075	0.3264	0.9458	0.2587
GPX3	rs3805435	***0.0355***	0.3907	0.2392	1.0000	***0.0251***	0.0996	0.1762	0.5750	0.8380	0.1363
GPX3	rs3828599	0.6441	0.1837	0.6508	0.7001	***0.0368***	0.1743	0.7133	0.0674	0.7072	0.3305
GPX3	rs8177425	***0.0300***	0.6436	***0.0040***	0.1006	0.2489	0.4742	0.8150	0.9999	0.9999	0.3026
HLA	rs9469220	0.5098	0.3760	0.4180	0.2910	0.0000751 *	0.2241	0.2165	0.6360	0.5656	0.4644
IBD5	rs10077785	0.6067	0.6985	0.3401	0.9251	0.2185	***0.0447***	0.7456	0.7740	0.9120	0.8265
IL12B	rs6887695	0.7405	***0.0399***	0.5853	***0.0159***	0.4712	0.5117	0.0807	0.2417	***0.0309***	***0.0131***
IL23R	rs11209026	***0.0239***	0.1584	0.5010	0.3494	0.4815	0.8682	0.5304	0.4450	0.8000	0.7140
IL23R	rs7517847	0.1036	***0.0194***	0.0845	***0.0040***	0.8793	***0.0253***	0.2731	0.3032	0.6869	0.9516
JAK2	rs10758669	0.6194	0.5804	***0.0112***	0.5926	0.6808	0.1891	0.4730	0.3900	0.9834	0.4026
JAM3	rs11604455	0.2993	0.7612	0.3313	0.7678	***0.0172***	0.9997	0.2734	0.8570	0.2700	0.9695
LAMP1	rs12871648	0.1360	0.8643	***0.0154***	0.4294	0.8006	1.0000	0.5318	0.4350	0.2880	0.2019
MAP1LC3B	rs8051218	0.8073	***0.0473***	0.4587	0.4253	0.9996	0.0709	0.0687	0.4940	0.4480	0.3652
NOD2	rs2066844	0.1270	0.8670	***0.0484***	0.4170	0.9760	0.1940	0.7067	***0.0463***	0.1670	0.7287
OCTN1	rs1050152	***0.0028***	0.0630	***0.0167***	0.1174	0.2692	0.9600	***0.0422***	0.0714	0.1230	0.0691
OCTN2	rs2631367	***0.0026***	0.1222	***0.0071***	0.0862	0.4066	0.7919	***0.0273***	***0.0322***	0.1665	***0.0317***
P4HA2	rs4361509	0.1683	0.2970	0.2569	0.0796	***0.0367***	0.2332	0.5564	0.1805	0.2497	***0.0298***
PTGER4	rs4613763	0.2721	0.0752	0.6681	***0.0364***	0.5922	0.5215	0.1237	***0.0210***	0.6250	0.4243
SELS	rs4965814	***0.0348***	***0.0326***	0.8601	0.6410	0.1583	0.3080	0.0646	0.6777	0.7620	0.8457
SELS	rs7178239	***0.0329***	***0.0219***	0.5408	0.9585	0.2971	0.1962	0.1168	0.5900	0.8680	0.9406
SEP_15	rs5845	0.4563	***0.0232***	0.9003	0.2900	0.6073	***0.0475***	0.5742	0.3418	0.0692	0.8078
SEP_15	rs5859	0.4050	***0.0149***	0.8041	0.2895	0.5395	***0.0287***	0.2979	0.3980	0.0511	0.4922
SLC11A1	rs3731865	0.7790	0.7499	***0.0302***	0.5538	0.9035	0.8647	0.6170	0.7168	0.4885	0.5708
SLC11A2	rs224589	0.1352	0.2771	0.3233	0.5982	***0.0433***	***0.0184***	0.2881	0.1440	0.5550	0.4478
SLC11A2	rs427020	0.6998	0.7563	***0.0494***	0.8253	***0.0098***	0.0772	0.2422	0.6477	0.8684	***0.0282***
SLC6A6	rs4685154	0.9413	0.3352	0.7719	***0.0359***	0.4094	0.6288	0.9841	0.5260	0.9491	0.3369
STAT3	rs744166	0.5240	***0.0327***	0.2239	0.2932	0.9202	0.4768	0.5676	0.6510	0.2256	0.6283
TF	rs1799899	***0.0269***	0.4295	0.6292	0.7491	0.8285	0.6787	0.6008	0.7040	0.5270	0.5817
TFF3	rs225369	0.1607	0.3494	***0.0171***	0.9542	0.8237	0.8419	0.8338	0.1928	0.7704	0.3020
TLR9	rs5743836	0.5591	***0.0495***	0.6591	0.3329	0.3057	0.4265	0.5340	0.9000	0.6370	0.2379
TNF.ALPHA	rs1800629	***0.0391***	0.8413	0.3526	0.3925	0.3941	0.2303	0.9387	0.2080	0.3790	0.1965
TNFSF15	rs10982412	0.8758	0.6216	0.7821	0.7470	0.6171	***0.0288***	0.9820	0.7528	0.2133	0.7304
TNFSF15	rs7029554	0.6069	0.7134	0.7987	0.8799	0.5054	***0.0272***	0.5918	0.5770	***0.0482***	0.4466
TRAIP	rs10865959	***0.0194***	0.3572	0.0580	0.3637	0.0823	0.4026	0.5807	0.9487	0.9680	0.0578
TRAIP	rs17598137	***0.0194***	0.3766	***0.0152***	0.7812	0.6667	0.2056	0.3028	0.5790	0.6020	0.4352
USP4	rs1865741	0.1737	0.2335	0.0663	***0.0337***	0.3255	0.6525	0.1860	0.6360	0.8208	0.6487
USP4	rs9881860	***0.0232***	0.1275	***0.0474***	0.0657	0.9879	0.8338	0.0635	0.7669	0.8724	0.4960
VDR	rs7975232	0.8557	0.5518	0.6355	0.6789	0.1431	0.4863	***0.0488***	0.6940	0.3780	0.4483

## 4. Discussion

On looking more closely at the candidate SNPs associated with CD that were tested for beneficial or adverse effects with the ingestion of selected Brassicaceae, we noted, as one would expect with an inflammatory disorder like Crohn’s disease that several of the genes associated with these SNPs were involved in functions relating to immunity. *AGT12* (rs26532) and *AGT16L1* (rs10210302) are necessary for autophagy; *CSFIR* (rs2282804), governs macrophages, *DEFA6* (rs712276) relates to the Paneth cells and its role in defence and *HLA* (rs9469220) a major histocompatibility complex, also involves the immune system. The *ICAM1gene*, (rs1799969) is expressed on the cells of the immune system and the endothelium [27]. *IL12B*, (rs6887695), has also been associated with another immune disorder, asthma [28]. *IRGM*, (rs4958847) is a gene associated with the autophagy pathway in CD [29]. *IL23R*, (rs11209026 and rs7517847) is another gene recognised as being involved in the adaptive immunity pathway as is *JAK2* (rs10758669) which is also essential for signalling events in innate immunity [28]. *PTPN2* (rs2542151) controls a range of cellular processes e.g., cell growth and *TLR9* (rs5743836) has a major role in both innate and adaptive immunity [28].

A number of SNPs are also associated with genes involved in cell transport, rs1050152 (*SLC22A4*/OCTN1) and rs2631367 (*SLC22A5*/*OCTN2)* linked to carnitine [30,31,32]; rs224589 and rs427020 (*SLC11A2*) linked to iron. *ITLN1* (rs2274910) and *TF* (rs1799899) are other genes associated with iron metabolism [28]. The SNPs rs41284011 and rs4685154 (*SLC6A6)* are linked with taurine which is linked to muscle function and having CD has been linked to diminished muscle strength [33,34,35]. Another gene of interest is the *VDR* gene (rs7975232) which facilitates the action of vitamin D3, as well as having a role in the homeostasis of calcium [36]. *CDH29* (rs7629936) is also a gene, with a SNP identified in this analysis, linked to calcium via calcium-reliant cell adhesion proteins [28]. Other genes of note are associated with responses of people with CD eating Brassicaceae are the *DIO1* (rs11206244 and rs75153322) and *DIO3* (rs1190715, rs1190716, and rs945006) genes which are important for the proper functioning of the thyroid gland. *DIO1*, also encodes selenoproteins [37,38] as does *GPX3*, (rs2042235, rs3763013, rs3792796, rs3792797, rs3805435, rs3828599, rs8177425, and rs870407, *GPX1* (rs1800668) and *GPX2* (rs1800669, rs2296327, rs2412065, rs2737844, rs37425990).

It is curious to note that six of the candidate genes identified have SNPS that are linked with both adverse and beneficial effects. The SNP rs7629936 (*CDH29*) is associated with adverse effects for cauliflower and beneficial effects for cabbage. The SNP rs2250114 (*FHIT*) is associated with adverse effects to Chinese greens and wasabi but beneficial effects for mustard powder and mustard sauce. The SNP rs3792796 (*GPX3*) is associated with adverse effects to mustard powder with beneficial effects to cauliflower and broccoli. The SNP rs2066844 (*NOD2*) is associated with adverse effects to cauliflower and mustard sauce and beneficial effects to mustard powder. The SNP rs5859 (*SEP15*) is associated with adverse effects to cabbage and watercress but beneficial effects to mustard powder. The SNP rs9881860 (*USP4*) is associated with adverse effects to broccoli and Chinese greens and beneficial effects to mustard sauce.

These differences in tolerance may relate to the different composition of each Brassicaceae. Brassicaceae differ in their nutritional components especially with respect to their phytochemical composition [8]. They are also unique in that they contain the phytochemicals known as glucosinolates. Up to two hundred different glucosinolates have been recorded [39]. These have been intensely researched because of their links with cancer reduction and properties associated with destroying or inhibiting the growth of bacteria, fungi and nematodes [40]. Each plant species of the Brassicaceae family may also contain up to four different classes of glucosinolates. The most common glucosinolates in cabbage are for example glucobrassicin, glucoraphanin, glucotropaeolin and sinigrin whereas in broccoli the abundant glucosinolates are glucoraphanin, glucobrassicin, gluconapin, and progoitrin [41]. Rocket’s predominate glucosinolates are glucoraphanin, DMB-GLS and glucoerucin [42].

When the vegetables are cooked, the glucosinolates reach the large intestine intact and the microbiota release the isothiocyanates and other metabolites with diverse outcomes [43]. The different combinations of glucosinolates in combination with particular SNPs may contribute to dissimilar outcomes produced by the microbiota. People with Crohn’s disease also have a microbiota community that differs from those with a normal gut [44]. So these differences too in the microbiota communities will also play a part in the difference in response. *Escherichia coli* are bacteria which have been identified as having increased numbers in those with Crohn’s disease [45,46,47,48]. They have also been associated with formation of granuloma (a common feature of Crohn’s disease) when internalised by macrophages *in vitro* [49]. *Escherichia coli* movement across Membranous cells (M-cells) has been shown *in vitro* to be reduced by soluble plant fibres such as broccoli [50]. [M-cells are part of the epithelium on the lymphoid follicles of the large intestine and act as a portal by which microorganisms can gain entry] [51].The ingestion of broccoli has also been shown in the IBD mouse model mdr1a(−/−) to lower inflammation in the large bowel through changes in the microbiota metabolism [52]. However, from the analysis provided in this paper, this response maybe modulated in a different way in humans depending on the particular SNP they have. For example the SNP rs9469220 variant in *HLA* and the SNP rs7515322 in *DIO1* which remained significant after correcting for multiple testing.

The rs9469220 variant in *HLA* was associated with an adverse reaction to the Brassicaceae rocket. *HLA* (human leucocyte antigen) genes are located in the region of MHCII in chromosome 6. This is very close to the HLA complexP5 (HCP5) which is associated with susceptibility to autoimmune diseases [53]. Variations in the *HLA* region have been associated with an inflammatory colonic phenotype [54,55,56]. *HLA* contains 4 classes and this SNP is with the class II MHC subgroup DO alpha. Both Class I and II *HLA* genes are necessary for normal lymphocyte performance [56]. This subgroup is associated with extracellular proteins that regulate peptide loading with antigens [57,58]. Class II molecules engage with CD4 T cells. These activate an immune response which may involve inflammation with the enlisting of phagocytes or activate B cells engaging antibodies in the immune response [59,60]. The polymorphism rs9469220 (identified in this study with an adverse response to rocket) has been linked to total IgE levels [61,62]. IgE has a pivotal role in type I sensitivity associated with allergic forms of asthma, rhinitis, urticaria and dermatitis [63]. IgE is also recognized as providing immunity to parasites [64]. The fact that the SNP rs9469220, in people with CD in this study is significantly associated with an adverse reaction to the Brassicaceae rocket would suggest that ingestion of this food engages the immune response in a type I sensitivity reaction. Hence an exacerbation of symptoms of CD occurs.

The other SNP which remained significant after correcting for multiple testing, was SNP (rs7515322) in *DIO1*. *DIO1* is part of the family of selenoenzymes which are important signalling molecules which activate or deactivate thyroid hormones, so play a key role in thyroid metabolism. As thyroid hormones are key to the development and metabolism of most tissues, these selenoproteins have a significant contribution to make. The *DIO1* gene is located in chromosome 1p33-p32 [28]. It is described as a thioredoxin fold integral membrane protein found in the plasma membrane. Transcripts of it have been identified in the intestines, thyroid, gonads, pituitary gland and placenta [65]. *DIO1* has a number of roles. It supplies a large portion of circulating plasma T_3_, it also acts as a scavenging enzyme removing inactive iodothyronines and recycling iodine, as well as playing a part in thyronamine biosynthesis [66,67,68]. Studies have also shown *DIO* activity associated with local inflammation and tumoural tissues [69,70]. *DIO1* as a selenoprotein has also been discovered to be protective against iodine deficiency when its activity is reduced with selenium deficiency. This was observed in regions of endemic goitre, when selenium was given to people with iodine deficiency with a consequent lowering of thyroid function [71]. Selenium levels have been reported as being significantly lower in New Zealand people with Crohn’s disease [72]. People in New Zealand with Crohn’s disease and low selenium levels and with this SNP rs7515322 may have also a heightened adverse response to Broccoli.

However, another mechanism may be involved. Critically ill people have been shown to have lower *DIO1* hepatic activity, and this has been thought to be modulated through Cytokines e.g., *IL1*, *IL6* and *TNF*α [73]. Cytokines may also decrease the function of *DIO1* and *IL-1*β has been shown to inhibit *DIO1* activities in hepatocarcinoma cell lines [74]. *IL-1*β is one of the cytokines increased in patients with Crohn’s disease [75] and associated with inflammation in this disorder. This SNP rs9469220 could link to an adverse response through progoitrin one of the most abundant glucosinolates in broccoli [41]. Progoitrin after ingestion is converted to goitrin by the activity in bacteria [76] and goitrin is known to decrease thyroid hormone production [77].

In addition, many adults in NZ are in a state of mild iodine deficiency with the adult population having a medium iodine concentration of 53 µg/L [78]. Schneider *et al.* in their murine study showed that *DIO1* may enable the major thyroid hormone T_3_ to be released into the circulatory system when an animal was iodine deficient [66]. Thyrocytes also react quickly to iodine deficiency by increasing the release of angiogenic signals. This is a VEGF-A dependent process [79]. VEGF production has been shown to be significantly increased with active Crohn’s disease [80].

Those people with CD having this SNP rs7515322 may with the ingestion of broccoli experience a greater degree of deficiency in iodine. This combined with a decrease in *DIO1* activity from the influence of *IL-1beta* activity associated with CD (*DIO1* normally increases with iodine deficiency) as well as an increased VEGF levels with the release of angiogenic signals with thyrocyte production, may exacerbate their symptoms of CD.

When investigating individual SNPs from this analysis of a New Zealand population with CD, it is interesting to note the two SNPs which are significantly associated with adverse effects (after correcting for multiple testing) to the two Brassicaceae—broccoli and rocket. Studying a wider range of Brassicaceae would increase the possibility of the identification of more significant SNPs and improve the range of Brassicaceae that would be available to choose from. If people want to avoid the long and tedious trial and error approach to working out what they can eat, or be more discerning about advice given to avoid many of the Brassicaceae as in the FODMAP diet [81] knowledge of their SNP profile and how it interacts with Brassicaceae would be helpful. It would enable people to select Brassicaceae more appropriately and maximise on their nutritional benefits.

## 5. Conclusions

It is now possible to identify two specific SNPs rs7515322 (*DIO1*), and rs9469220 (*HLA*) associated with the two forms of Brassicaceae broccoli and rocket respectively, with regard to having an adverse effect on the symptoms of people with CD. Further research is required to substantiate our findings and to conclusively determine the nature of the observed association of an adverse effect with the two forms of Brassicaceae broccoli and rocket and the two SNPs rs7515322 (*DIO1*), and rs9469220 (*HLA*) respectively, with CD. This study indicates that the tolerance of some varieties of Brassicaceae may be predicted by analysis of a person’s genotype. This information is a step towards enabling those with CD to select appropriate Brassicaceae and be exposed to key nutrients. This raises the possibility that with suitable nutrition, there is a real prospect of a significant improvement in symptoms associated with this disorder.

## Abbreviations

CD: Crohn’s Disease
IBD: inflammatory bowel disease
NZ: New Zealand
SNP: Single nucleotide polymorphism
UC: Ulcerative Colitis

## Supplementary Files

Supplementary File 1 Supplementary Information (PDF, 510 KB)
