# Supplementary material for: Candidate Genes Involved in Beneficial or Adverse Responses to Commonly Eaten Brassica Vegetables in a New Zealand Crohn’s Disease Cohort
Source: Nutrients. 2013 Dec 12;5(12):5046–64. doi: 10.3390/nu5125046 (PMC3875924; doi:10.3390/nu5125046)
Supplement: Supplementary File 1 — Supplementary Information (PDF, 510 KB) [file nutrients-05-05046-s001.pdf]

Supplementary Information

Table S1. Brassicaceae frequencies [*n* (%)] by SNPs.

		**AMT rs1464567**			**ATG12 rs26532**			**ATG16L1 rs10210302**			**BSN rs2131109**		
		C/C	C/G	G/G	A/A	A/C	C/C	C/C	C/T	T/T	A/A	A/G	G/G
Broccoli	No Difference	30 (16.4)	101 (55.2)	52 (28.4)	105 (57.7)	66 (36.3)	11 (6.0)	35 (19.2)	87 (47.8)	60 (33.0)	41 (22.5)	95 (52.2)	46 (25.3)
	Beneficial	3 (15)	10 (50.0)	7 (35.0)	9 (40.9)	12 (54.6)	1 (4.6)	2 (9.1)	14 (63.6)	6 (27.3)	5 (23.8)	9 (42.9)	7 (33.3)
	Adverse	5 (9.6)	22 (42.3)	25 (48.1)	35 (67.3)	16 (30.8)	1 (1.9)	8 (16.0)	27 (54)	15 (30.0)	15 (28.9)	28 (53.9)	9 (17.3)
Cabbage	No Difference	19 (15.1)	69 (54.8)	38 (30.2)	81 (64.3)	36 (28.6)	9 (7.1)	18 (14.5)	60 (48.4)	46 (37.1)	29 (23.2)	66 (52.8)	30 (24.0)
	Beneficial	3 (42.9)	2 (28.6)	2 (28.6)	3 (42.9)	3 (42.9)	1 (14.3)	1 (14.3)	4 (57.1)	2 (28.6)	2 (28.6)	1 (14.3)	4 (57.1)
	Adverse	20 (16.0)	64 (51.2)	41 (32.8)	70 (56.0)	51 (40.8)	4 (3.2)	27 (21.6)	66 (52.8)	32 (25.6)	28 (22.4)	65 (52.0)	32 (25.6)
Cauliflower	No Difference	30 (16.8)	98 (54.8)	51 (28.5)	105 (59.3)	62 (35.0)	10 (5.7)	31 (17.5)	84 (47.5)	62 (35.0)	41 (23.0)	87 (48.9)	50 (28.1)
	Beneficial	3 (17.7)	9 (52.9)	5 (29.4)	6 (31.6)	12 (63.2)	1 (5.3)	2 (10.5)	11 (57.9)	6 (31.6)	4 (21.1)	8 (42.1)	7 (36.8)
	Adverse	8 (12.7)	26 (41.3)	29 (46.0)	42 (65.6)	20 (31.3)	2 (3.1)	11 (17.7)	37 (59.7)	14 (22.6)	17 (27.4)	39 (62.9)	6 (9.7)
Chinese Greens	No Difference	25 (22.1)	60 (53.1)	28 (24.8)	64 (56.1)	42 (36.8)	8 (7.0)	17 (15.2)	56 (50.0)	39 (34.8)	23 (20.7)	55 (49.6)	33 (29.7)
	Beneficial	0 (0)	6 (75.0)	2 (25.0)	2 (22.2)	7 (77.8)	0 (0)	0 (0)	4 (50.0)	4 (50.0)	2 (22.2)	5 (55.6)	2 (22.2)
	Adverse	2 (5.9)	19 (55.9)	13 (38.2)	20 (60.6)	11 (33.3)	2 (6.1)	7 (21.2)	20 (60.6)	6 (18.2)	7 (21.2)	19 (57.6)	7 (21.2)
Rocket	No Difference	26 (16.6)	83 (52.9)	48 (30.6)	89 (57.4)	58 (37.4)	8 (5.2)	23 (14.9)	75 (48.7)	56 (36.4)	36 (22.9)	82 (52.2)	39 (24.8)
	Beneficial	1 (20.0)	3 (60.0)	1 (20.0)	1 (20.0)	3 (60.0)	1 (20.0)	0 (0)	4 (80.0)	1 (20.0)	0 (0)	2 (40.0)	3 (60.0)
	Adverse	2 (8.0)	12 (48.0)	11 (44.0)	13 (52.0)	11 (44.0)	1 (4.0)	4 (16.7)	14 (58.3)	6 (25.0)	7 (29.2)	13 (54.2)	4 (16.7)
Watercress	No Difference	14 (18.9)	39 (52.7)	21 (28.4)	44 (58.7)	25 (33.3)	6 (8.0)	10 (13.7)	38 (52.1)	25 (34.3)	15 (21.1)	35 (49.3)	21 (29.6)

Table S1. *Cont.*

		**AMT rs1464567**			**ATG12 rs26532**			**ATG16L1 rs10210302**			**BSN rs2131109**		
		C/C	C/G	G/G	A/A	A/C	C/C	C/C	C/T	T/T	A/A	A/G	G/G
	Beneficial	1 (12.5)	7 (87.5)	0 (0)	4 (44.4)	5 (55.6)	0 (0)	0 (0)	4 (44.4)	5 (55.6)	1 (11.1)	4 (44.4)	4 (44.4)
	Adverse	0 (0)	10 (58.8)	7 (41.2)	10 (58.8)	7 (41.2)	0 (0)	3 (17.7)	9 (52.9)	5 (29.4)	4 (23.5)	11 (64.7)	2 (11.8)
Horseradish	No Difference	13 (14.4)	53 (58.9)	24 (26.7)	51 (58.0)	31 (35.2)	6 (6.8)	16 (18)	42 (47.2)	31 (34.8)	22 (24.7)	44 (49.4)	23 (25.8)
	Beneficial	1 (50.0)	1 (50.0)	0 (0)	1 (50.0)	0 (0)	1 (50.0)	0 (0)	1 (100)	0 (0)	0 (0)	1 (50.0)	1 (50.0)
	Adverse	9 (19.2)	23 (48.9)	15 (31.9)	19 (39.6)	27 (56.3)	2 (4.2)	10 (20.8)	26 (54.2)	12 (25.0)	9 (18.8)	25 (52.1)	14 (29.2)
Mustard powder	No Difference	26 (16.2)	81 (50.3)	54 (33.5)	101 (62.7)	53 (32.9)	7 (4.4)	27 (16.9)	78 (48.8)	55 (34.4)	41 (25.8)	80 (50.3)	38 (23.9)
	Beneficial	1 (33.3)	2 (66.7)	0 (0)	0 (0)	2 (66.7)	1 (33.3)	0 (0)	2 (66.7)	1 (33.3)	0 (0)	1 (33.3)	2 (66.7)
	Adverse	8 (14.6)	34 (61.8)	13 (23.6)	29 (51.8)	25 (44.6)	2 (3.6)	12 (22.6)	24 (45.3)	17 (32.1)	11 (19.6)	31 (55.4)	14 (25.0)
Mustard sauce	No Difference	26 (17.3)	79 (52.7)	45 (30.0)	99 (65.1)	46 (30.3)	7 (4.6)	23 (15.4)	76 (51)	50 (33.6)	37 (24.8)	74 (49.7)	38 (25.5)
	Beneficial	1 (25.0)	1 (25.0)	2 (50.0)	0 (0)	3 (75.0)	1 (25.0)	0 (0)	3 (75.0)	1 (25.0)	1 (25.0)	2 (50.0)	1 (25.0)
	Adverse	11 (16.9)	36 (55.4)	18 (27.7)	32 (49.2)	30 (46.2)	3 (4.6)	17 (26.2)	32 (49.2)	16 (24.6)	13 (20.0)	35 (53.9)	17 (26.2)
Wasabi	No Difference	14 (17.7)	41 (51.9)	24 (30.4)	45 (57.0)	29 (36.7)	5 (6.3)	13 (16.5)	40 (50.6)	26 (32.9)	19 (24.4)	37 (47.4)	22 (28.2)
	Beneficial	1 (33.3)	1 (33.3)	1 (33.3)	0 (0)	2 (66.7)	1 (33.3)	0 (0)	3 (100)	0 (0)	1 (33.3)	1 (33.3)	1 (33.3)
	Adverse	7 (11.7)	33 (55.0)	20 (33.3)	32 (54.2)	24 (40.7)	3 (5.1)	13 (21.7)	30 (50.0)	17 (28.3)	13 (21.7)	36 (60.0)	11 (18.3)

Table S1. *Cont.*

		BSN rs4283605			CDH29 rs7629936			CDKAL1 rs6908425			CLDN12 rs1017106		
		A/A	A/G	G/G	A/A	A/G	G/G	C/C	C/T	T/T	C/C	C/T	T/T
Broccoli	No Difference	75 (40.5)	89 (48.1)	21 (11.4)	13 (7.1)	64 (35.0)	106 (57.9)	121 (65.8)	52 (28.3)	11 (6.0)	0 (0)	14 (7.7)	169 (92.4)
	Beneficial	9 (40.9)	8 (36.4)	5 (22.7)	0 (0)	8 (40.0)	12 (60.0)	11 (50.0)	11 (50.0)	0 (0)	0 (0)	1 (4.6)	21 (95.5)
	Adverse	20 (38.5)	23 (44.2)	9 (17.3)	2 (3.9)	11 (21.2)	39 (75.0)	34 (66.7)	14 (27.5)	3 (5.9)	0 (0)	4 (7.7)	48 (92.3)
Cabbage	No Difference	52 (40.9)	58 (45.7)	17 (13.4)	8 (6.4)	45 (35.7)	73 (57.9)	86 (68.3)	31 (24.6)	9 (7.1)	0 (0)	14 (11.4)	109 (88.6)
	Beneficial	4 (57.1)	1 (14.3)	2 (28.6)	1 (14.3)	4 (57.1)	2 (28.6)	4 (57.1)	3 (42.9)	0 (0)	0 (0)	0 (0)	7 (100)
	Adverse	53 (41.7)	59 (46.5)	15 (11.8)	8 (6.4)	40 (32)	77 (61.6)	82 (65.1)	40 (31.8)	4 (3.2)	0 (0)	6 (4.7)	123 (95.4)
Cauliflower	No Difference	77 (42.8)	79 (43.9)	24 (13.3)	14 (7.8)	62 (34.6)	103 (57.5)	120 (66.7)	50 (27.8)	10 (5.6)	0 (0)	15 (8.4)	164 (91.6)
	Beneficial	9 (47.4)	7 (36.8)	3 (15.8)	0 (0)	8 (47.1)	9 (52.9)	8 (42.1)	11 (57.9)	0 (0)	0 (0)	1 (5.3)	18 (94.7)
	Adverse	20 (31.3)	35 (54.7)	9 (14.1)	1 (1.6)	17 (27)	45 (71.4)	43 (69.4)	15 (24.2)	4 (6.5)	0 (0)	3 (4.8)	60 (95.2)
Chinese Greens	No Difference	53 (46.9)	46 (40.7)	14 (12.4)	11 (9.7)	41 (36.3)	61 (54)	73 (64.6)	32 (28.3)	8 (7.1)	0 (0)	11 (10.0)	99 (90.0)
	Beneficial	2 (22.2)	6 (66.7)	1 (11.1)	0 (0)	4 (50.0)	4 (50.0)	4 (44.4)	5 (55.6)	0 (0)	0 (0)	0 (0)	9 (100)
	Adverse	14 (41.2)	15 (44.1)	5 (14.7)	1 (2.9)	10 (29.4)	23 (67.7)	21 (63.6)	11 (33.3)	1 (3)	0 (0)	0 (0)	33 (100)
Rocket	No Difference	67 (42.4)	72 (45.6)	19 (12.0)	9 (5.7)	65 (41.4)	83 (52.9)	101 (64.7)	44 (28.2)	11 (7.1)	0 (0)	16 (10.3)	139 (89.7)
	Beneficial	3 (60.0)	2 (40.0)	0 (0)	0 (0)	3 (60.0)	2 (40.0)	2 (40.0)	3 (60.0)	0 (0)	0 (0)	0 (0)	5 (100)
	Adverse	7 (28.0)	14 (56.0)	4 (16.0)	2 (8.0)	5 (20.0)	18 (72)	18 (75.0)	6 (25.0)	0 (0)	0 (0)	0 (0)	24 (100)
Watercress	No Difference	33 (44.6)	36 (48.7)	5 (6.8)	6 (8.1)	24 (32.4)	44 (59.5)	51 (68.9)	19 (25.7)	4 (5.4)	0 (0)	4 (5.6)	67 (94.4)
	Beneficial	5 (55.6)	3 (33.3)	1 (11.1)	0 (0)	5 (62.5)	3 (37.5)	5 (55.6)	4 (44.4)	0 (0)	0 (0)	0 (0)	9 (100)
	Adverse	7 (41.2)	8 (47.1)	2 (11.8)	0 (0)	3 (17.7)	14 (82.4)	8 (50.0)	6 (37.5)	2 (12.5)	0 (0)	1 (5.9)	16 (94.1)
Horseradish	No Difference	37 (41.6)	39 (43.8)	13 (14.6)	6 (6.7)	33 (36.7)	51 (56.7)	60 (66.7)	25 (27.8)	5 (5.6)	0 (0)	12 (13.6)	76 (86.4)
	Beneficial	1 (50.0)	1 (50.0)	0 (0)	0 (0)	1 (50.0)	1 (50.0)	0 (0)	2 (100)	0 (0)	0 (0)	0 (0)	2 (100)
	Adverse	25 (52.1)	20 (41.7)	3 (6.3)	3 (6.4)	14 (29.8)	30 (63.8)	26 (55.3)	18 (38.3)	3 (6.4)	0 (0)	1 (2.1)	47 (97.9)
Mustard powder	No Difference	68 (42.5)	69 (43.1)	23 (14.4)	9 (5.6)	54 (33.5)	98 (60.9)	110 (67.5)	44 (27)	9 (5.5)	0 (0)	16 (9.9)	145 (90.1)
	Beneficial	2 (66.7)	1 (33.3)	0 (0)	0 (0)	3 (100)	0 (0)	1 (33.3)	2 (66.7)	0 (0)	0 (0)	0 (0)	3 (100)
	Adverse	22 (38.6)	29 (50.9)	6 (10.5)	4 (7.3)	20 (36.4)	31 (56.4)	36 (66.7)	17 (31.5)	1 (1.9)	0 (0)	3 (5.5)	52 (94.6)
Mustard sauce	No Difference	62 (41.3)	65 (43.3)	23 (15.3)	10 (6.7)	53 (35.3)	87 (58.0)	106 (69.7)	40 (26.3)	6 (4.0)	0 (0)	16 (10.7)	133 (89.3)
	Beneficial	3 (75.0)	1 (25.0)	0 (0)	0 (0)	1 (25.0)	3 (75.0)	1 (25.0)	3 (75.0)	0 (0)	0 (0)	0 (0)	4 (100)
	Adverse	27 (40.3)	33 (49.3)	7 (10.5)	4 (6.2)	23 (35.4)	38 (58.5)	40 (61.5)	22 (33.9)	3 (4.6)	0 (0)	2 (3)	65 (97.0)
Wasabi	No Difference	36 (46.2)	30 (38.5)	12 (15.4)	6 (7.6)	30 (38.0)	43 (54.4)	57 (71.3)	19 (23.8)	4 (5)	0 (0)	10 (12.7)	69 (87.3)
	Beneficial	2 (66.7)	1 (33.3)	0 (0)	0 (0)	2 (66.7)	1 (33.3)	2 (66.7)	1 (33.3)	0 (0)	0 (0)	0 (0)	3 (100)
	Adverse	25 (41)	31 (50.8)	5 (8.2)	2 (3.3)	15 (25.0)	43 (71.7)	34 (58.6)	21 (36.2)	3 (5.2)	0 (0)	1 (1.7)	57 (98.3)

Table S1. *Cont.*

		CLDN12 rs17864006			CLDN2 rs12008279			CNTNAP2 rs7807268			CSF1R rs2282804		
		A/A	A/C	C/C	A/A	A/G	G/G	C/C	C/G	G/G	A/A	A/T	T/T
Broccoli	No Difference	0 (0)	12 (6.6)	171 (93.4)	62 (34.1)	66 (36.3)	54 (29.7)	45 (24.7)	91 (50.0)	46 (25.3)	37 (30.3)	46 (37.7)	39 (32)
Broccoli	Beneficial	0 (0)	4 (18.2)	18 (81.8)	9 (40.9)	4 (18.2)	9 (40.9)	7 (31.8)	7 (31.8)	8 (36.4)	4 (28.6)	4 (28.6)	6 (42.9)
Broccoli	Adverse	0 (0)	6 (11.5)	46 (88.5)	18 (34.6)	22 (42.3)	12 (23.1)	14 (28.6)	28 (57.1)	7 (14.3)	9 (23.1)	24 (61.5)	6 (15.4)
Cabbage	No Difference	0 (0)	8 (6.4)	118 (93.7)	41 (33.1)	41 (33.1)	42 (33.9)	34 (27.9)	57 (46.7)	31 (25.4)	28 (32.6)	33 (38.4)	25 (29.1)
Cabbage	Beneficial	0 (0)	1 (14.3)	6 (85.7)	4 (66.7)	1 (16.7)	1 (16.7)	1 (14.3)	3 (42.9)	3 (42.9)	1 (50.0)	1 (50.0)	0 (0)
Cabbage	Adverse	0 (0)	14 (11.1)	112 (88.9)	43 (33.9)	46 (36.2)	38 (29.9)	30 (24.0)	65 (52.0)	30 (24.0)	23 (26.4)	42 (48.3)	22 (25.3)
Cauliflower	No Difference	0 (0)	12 (6.7)	167 (93.3)	62 (34.4)	65 (36.1)	53 (29.4)	47 (26.4)	84 (47.2)	47 (26.4)	40 (31.8)	50 (39.7)	36 (28.6)
Cauliflower	Beneficial	0 (0)	3 (15.8)	16 (84.2)	8 (42.1)	4 (21.1)	7 (36.8)	5 (26.3)	6 (31.6)	8 (42.1)	4 (33.3)	4 (33.3)	4 (33.3)
Cauliflower	Adverse	0 (0)	7 (11.1)	56 (88.9)	20 (32.8)	25 (41)	16 (26.2)	15 (25.0)	35 (58.3)	10 (16.7)	9 (22)	22 (53.7)	10 (24.4)
Chinese Greens	No Difference	0 (0)	5 (4.4)	109 (95.6)	35 (32.1)	37 (33.9)	37 (33.9)	39 (34.5)	53 (46.9)	21 (18.6)	24 (29.3)	30 (36.6)	28 (34.2)
Chinese Greens	Beneficial	0 (0)	0 (0)	8 (100)	4 (50.0)	0 (0)	4 (50.0)	2 (28.6)	1 (14.3)	4 (57.1)	2 (40.0)	2 (40.0)	1 (20.0)
Chinese Greens	Adverse	0 (0)	5 (15.2)	28 (84.9)	14 (42.4)	13 (39.4)	6 (18.2)	10 (32.3)	15 (48.4)	6 (19.4)	7 (28.0)	11 (44.0)	7 (28.0)
Rocket	No Difference	0 (0)	14 (9)	141 (91)	50 (32.1)	50 (32.1)	56 (35.9)	44 (28.8)	71 (46.4)	38 (24.8)	34 (29.6)	46 (40.0)	35 (30.4)
Rocket	Beneficial	0 (0)	0 (0)	5 (100)	2 (40.0)	2 (40.0)	1 (20.0)	3 (60.0)	0 (0)	2 (40.0)	0 (0)	1 (50.0)	1 (50.0)
Rocket	Adverse	0 (0)	4 (16.0)	21 (84)	11 (45.8)	10 (41.7)	3 (12.5)	9 (39.1)	12 (52.2)	2 (8.7)	4 (22.2)	9 (50.0)	5 (27.8)
Watercress	No Difference	0 (0)	5 (6.8)	69 (93.2)	20 (27.8)	22 (30.6)	30 (41.7)	23 (31.5)	36 (49.3)	14 (19.2)	16 (32)	16 (32)	18 (36)
Watercress	Beneficial	0 (0)	1 (11.1)	8 (88.9)	2 (25.0)	4 (50.0)	2 (25.0)	2 (22.2)	3 (33.3)	4 (44.4)	2 (28.6)	3 (42.9)	2 (28.6)
Watercress	Adverse	0 (0)	1 (5.9)	16 (94.1)	4 (23.5)	8 (47.1)	5 (29.4)	7 (41.2)	8 (47.1)	2 (11.8)	7 (46.7)	5 (33.3)	3 (20.0)
Horseradish	No Difference	0 (0)	7 (8.0)	81 (92.1)	31 (34.8)	29 (32.6)	29 (32.6)	30 (34.1)	40 (45.5)	18 (20.5)	17 (27)	28 (44.4)	18 (28.6)
Horseradish	Beneficial	0 (0)	0 (0)	2 (100)	2 (100)	0 (0)	0 (0)	0 (0)	0 (0)	1 (100)	0 (0)	1 (100)	0 (0)
Horseradish	Adverse	0 (0)	5 (10.4)	43 (89.6)	12 (25.5)	15 (31.9)	20 (42.6)	10 (20.8)	26 (54.2)	12 (25.0)	13 (50.0)	8 (30.8)	5 (19.2)
Mustard powder	No Difference	0 (0)	13 (8.1)	148 (91.9)	55 (34.2)	53 (32.9)	53 (32.9)	45 (28.1)	79 (49.4)	36 (22.5)	35 (31.8)	44 (40.0)	31 (28.2)
Mustard powder	Beneficial	0 (0)	0 (0)	3 (100)	2 (66.7)	1 (33.3)	0 (0)	1 (33.3)	0 (0)	2 (66.7)	0 (0)	0 (0)	0 (0)
Mustard powder	Adverse	0 (0)	6 (10.7)	50 (89.3)	20 (35.7)	19 (33.9)	17 (30.4)	14 (27.5)	27 (52.9)	10 (19.6)	13 (29.6)	20 (45.5)	11 (25.0)
Mustard sauce	No Difference	0 (0)	13 (8.6)	139 (91.5)	51 (34.5)	50 (33.8)	47 (31.8)	43 (28.9)	73 (49)	33 (22.2)	31 (29.3)	46 (43.4)	29 (27.4)
Mustard sauce	Beneficial	0 (0)	1 (25.0)	3 (75.0)	2 (50.0)	1 (25.0)	1 (25.0)	1 (25.0)	0 (0)	3 (75.0)	0 (0)	1 (100)	0 (0)
Mustard sauce	Adverse	0 (0)	6 (9.2)	59 (90.8)	22 (33.3)	23 (34.9)	21 (31.8)	16 (25.4)	34 (54)	13 (20.6)	16 (34.8)	20 (43.5)	10 (21.7)
Wasabi	No Difference	0 (0)	7 (8.8)	73 (91.3)	27 (34.2)	24 (30.4)	28 (35.4)	23 (29.5)	39 (50.0)	16 (20.5)	13 (23.2)	22 (39.3)	21 (37.5)
Wasabi	Beneficial	0 (0)	0 (0)	3 (100)	2 (66.7)	1 (33.3)	0 (0)	1 (33.3)	0 (0)	2 (66.7)	0 (0)	0 (0)	1 (100)
Wasabi	Adverse	0 (0)	6 (10.0)	54 (90.0)	20 (34.5)	20 (34.5)	18 (31)	16 (26.7)	30 (50.0)	14 (23.3)	17 (42.5)	16 (40.0)	7 (17.5)

Table S1. *Cont.*

		DEFA6	rs712276		desert-PTGER4	rs17234657		desert-PTGER4	rs9292777		DIO1	rs11206244	
		C/C	C/T	T/T	G/G	G/T	T/T	C/C	C/T	T/T	C/C	C/T	T/T
Broccoli	No Difference	2 (1.1)	47 (25.7)	134 (73.2)	3 (1.6)	48 (26)	134 (72.4)	19 (10.4)	81 (44.3)	83 (45.4)	93 (51.4)	69 (38.1)	19 (10.5)
Broccoli	Beneficial	1 (4.6)	10 (45.5)	11 (50.0)	0 (0)	6 (27.3)	16 (72.7)	4 (18.2)	11 (50.0)	7 (31.8)	9 (40.9)	12 (54.6)	1 (4.6)
Broccoli	Adverse	0 (0)	16 (31.4)	35 (68.6)	2 (4.1)	14 (28.6)	33 (67.4)	2 (3.9)	22 (43.1)	27 (52.9)	20 (39.2)	26 (51)	5 (9.8)
Cabbage	No Difference	1 (0.8)	38 (30.4)	86 (68.8)	2 (1.6)	30 (24.0)	93 (74.4)	16 (12.9)	54 (43.6)	54 (43.6)	67 (54.9)	45 (36.9)	10 (8.2)
Cabbage	Beneficial	1 (14.3)	4 (57.1)	2 (28.6)	0 (0)	1 (14.3)	6 (85.7)	0 (0)	5 (71.4)	2 (28.6)	4 (66.7)	0 (0)	2 (33.3)
Cabbage	Adverse	1 (0.8)	32 (25.4)	93 (73.8)	5 (4.0)	40 (31.8)	81 (64.3)	11 (8.7)	53 (41.7)	63 (49.6)	52 (40.9)	62 (48.8)	13 (10.2)
Cauliflower	No Difference	2 (1.1)	52 (29.1)	125 (69.8)	5 (2.8)	47 (26.1)	128 (71.1)	19 (10.7)	80 (44.9)	79 (44.4)	91 (51.1)	70 (39.3)	17 (9.6)
Cauliflower	Beneficial	1 (5.3)	8 (42.1)	10 (52.6)	0 (0)	5 (26.3)	14 (73.7)	3 (15.8)	10 (52.6)	6 (31.6)	8 (42.1)	10 (52.6)	1 (5.3)
Cauliflower	Adverse	0 (0)	16 (25.8)	46 (74.2)	0 (0)	17 (27.9)	44 (72.1)	4 (6.4)	26 (41.3)	33 (52.4)	27 (44.3)	28 (45.9)	6 (9.8)
Chinese Greens	No Difference	1 (0.9)	30 (26.6)	82 (72.6)	2 (1.8)	30 (26.6)	81 (71.7)	9 (8.0)	48 (42.9)	55 (49.1)	60 (55.1)	41 (37.6)	8 (7.3)
Chinese Greens	Beneficial	1 (11.1)	3 (33.3)	5 (55.6)	0 (0)	2 (25.0)	6 (75.0)	2 (22.2)	5 (55.6)	2 (22.2)	2 (28.6)	5 (71.4)	0 (0)
Chinese Greens	Adverse	0 (0)	11 (33.3)	22 (66.7)	1 (3)	14 (42.4)	18 (54.6)	3 (9.1)	11 (33.3)	19 (57.6)	13 (39.4)	17 (51.5)	3 (9.1)
Rocket	No Difference	1 (0.6)	38 (24.4)	117 (75.0)	4 (2.6)	42 (26.9)	110 (70.5)	13 (8.3)	67 (43)	76 (48.7)	79 (51.3)	60 (39)	15 (9.7)
Rocket	Beneficial	1 (20.0)	2 (40.0)	2 (40.0)	0 (0)	0 (0)	5 (100)	1 (20.0)	3 (60.0)	1 (20.0)	2 (40.0)	2 (40.0)	1 (20.0)

Table S1. *Cont.*

		**DEFA6 rs712276**			**desert-PTGER4 rs17234657**			**desert-PTGER4 rs9292777**			**DIO1 rs11206244**		
		C/C	C/T	T/T	G/G	G/T	T/T	C/C	C/T	T/T	C/C	C/T	T/T
Rocket	Adverse	0 (0)	6 (26.1)	17 (73.9)	0 (0)	8 (33.3)	16 (66.7)	3 (12.5)	10 (41.7)	11 (45.8)	8 (33.3)	13 (54.2)	3 (12.5)
Watercress	No Difference	1 (1.4)	21 (28.4)	52 (70.3)	3 (4.1)	18 (24.3)	53 (71.6)	7 (9.6)	32 (43.8)	34 (46.6)	36 (50.7)	31 (43.7)	4 (5.6)
Watercress	Beneficial	1 (11.1)	5 (55.6)	3 (33.3)	0 (0)	2 (22.2)	7 (77.8)	1 (11.1)	5 (55.6)	3 (33.3)	5 (62.5)	3 (37.5)	0 (0)
Watercress	Adverse	0 (0)	5 (29.4)	12 (70.6)	0 (0)	5 (31.3)	11 (68.8)	3 (17.7)	2 (11.8)	12 (70.6)	7 (41.2)	9 (52.9)	1 (5.9)
Horseradish	No Difference	1 (1.1)	26 (28.9)	63 (70.0)	1 (1.1)	23 (25.6)	66 (73.3)	6 (6.7)	38 (42.2)	46 (51.1)	44 (50.6)	37 (42.5)	6 (6.9)
Horseradish	Beneficial	0 (0)	1 (50.0)	1 (50.0)	0 (0)	0 (0)	1 (100)	0 (0)	2 (100)	0 (0)	0 (0)	1 (50.0)	1 (50.0)
Horseradish	Adverse	0 (0)	15 (31.3)	33 (68.8)	1 (2.1)	18 (38.3)	28 (59.6)	6 (12.5)	18 (37.5)	24 (50.0)	14 (29.8)	27 (57.5)	6 (12.8)
Mustard powder	No Difference	1 (0.6)	48 (29.6)	113 (69.8)	5 (3.1)	34 (20.9)	124 (76.1)	14 (8.7)	73 (45.3)	74 (46.0)	81 (50.3)	61 (37.9)	19 (11.8)
Mustard powder	Beneficial	1 (33.3)	1 (33.3)	1 (33.3)	0 (0)	0 (0)	3 (100)	0 (0)	2 (66.7)	1 (33.3)	1 (33.3)	1 (33.3)	1 (33.3)
Mustard powder	Adverse	1 (1.9)	11 (20.4)	42 (77.8)	1 (1.9)	22 (41.5)	30 (56.6)	8 (14.8)	24 (44.4)	22 (40.7)	20 (37)	29 (53.7)	5 (9.3)
Mustard sauce	No Difference	2 (1.3)	46 (30.5)	103 (68.2)	4 (2.6)	37 (24.3)	111 (73)	12 (8.0)	69 (46.0)	69 (46.0)	76 (51.7)	55 (37.4)	16 (10.9)
Mustard sauce	Beneficial	0 (0)	1 (25.0)	3 (75.0)	0 (0)	1 (25.0)	3 (75.0)	1 (25.0)	2 (50.0)	1 (25.0)	1 (25.0)	1 (25.0)	2 (50.0)
Mustard sauce	Adverse	0 (0)	18 (27.3)	48 (72.7)	0 (0)	21 (32.8)	43 (67.2)	9 (13.6)	24 (36.4)	33 (50.0)	23 (35.4)	35 (53.9)	7 (10.8)
Wasabi	No Difference	2 (2.5)	24 (30.4)	53 (67.1)	2 (2.5)	20 (25.0)	58 (72.5)	7 (8.8)	34 (42.5)	39 (48.8)	40 (50.6)	33 (41.8)	6 (7.6)
Wasabi	Beneficial	0 (0)	1 (33.3)	2 (66.7)	0 (0)	0 (0)	3 (100)	1 (33.3)	2 (66.7)	0 (0)	0 (0)	1 (33.3)	2 (66.7)
Wasabi	Adverse	0 (0)	14 (23.3)	46 (76.7)	1 (1.7)	22 (37.3)	36 (61)	5 (8.3)	24 (40.0)	31 (51.7)	23 (40.4)	29 (50.9)	5 (8.8)

Table S1. *Cont.*

		DIO1	rs7515322		DIO2	rs12885300		DIO2	rs2250114		DIO3	rs1190715	
		A/A	A/G	G/G	C/C	C/T	T/T	A/A	A/G	G/G	C/C	C/T	T/T
Broccoli	No Difference	179 (97.3)	5 (2.7)	0 (0)	62 (33.7)	96 (52.2)	26 (14.1)	112 (61.2)	61 (33.3)	10 (5.5)	54 (31.8)	84 (49.4)	32 (18.8)
Broccoli	Beneficial	19 (86.4)	3 (13.6)	0 (0)	9 (40.9)	11 (50.0)	2 (9.1)	13 (59.1)	7 (31.8)	2 (9.1)	2 (10.0)	14 (70.0)	4 (20.0)
Broccoli	Adverse	43 (82.7)	9 (17.3)	0 (0)	24 (46.2)	21 (40.4)	7 (13.5)	38 (74.5)	10 (19.6)	3 (5.9)	11 (21.6)	23 (45.1)	17 (33.3)
Cabbage	No Difference	120 (96.8)	4 (3.2)	0 (0)	44 (35.2)	66 (52.8)	15 (12.0)	79 (63.7)	39 (31.5)	6 (4.8)	29 (25.2)	61 (53)	25 (21.7)
Cabbage	Beneficial	6 (85.7)	1 (14.3)	0 (0)	4 (57.1)	2 (28.6)	1 (14.3)	2 (33.3)	2 (33.3)	2 (33.3)	1 (14.3)	5 (71.4)	1 (14.3)
Cabbage	Adverse	119 (92.3)	10 (7.8)	0 (0)	46 (35.9)	63 (49.2)	19 (14.8)	79 (62.2)	40 (31.5)	8 (6.3)	38 (31.2)	57 (46.7)	27 (22.1)
Cauliflower	No Difference	172 (95.6)	8 (4.4)	0 (0)	65 (35.9)	90 (49.7)	26 (14.4)	110 (61.1)	59 (32.8)	11 (6.1)	48 (28.4)	86 (50.9)	35 (20.7)
Cauliflower	Beneficial	16 (84.2)	3 (15.8)	0 (0)	8 (42.1)	9 (47.4)	2 (10.5)	11 (57.9)	7 (36.8)	1 (5.3)	2 (11.8)	11 (64.7)	4 (23.5)
Cauliflower	Adverse	57 (90.5)	6 (9.5)	0 (0)	21 (33.3)	35 (55.6)	7 (11.1)	44 (72.1)	14 (23.0)	3 (4.9)	18 (30.0)	26 (43.3)	16 (26.7)
Chinese Greens	No Difference	105 (94.6)	6 (5.4)	0 (0)	39 (34.8)	60 (53.6)	13 (11.6)	74 (67.3)	32 (29.1)	4 (3.6)	30 (28.3)	57 (53.8)	19 (17.9)
Chinese Greens	Beneficial	8 (88.9)	1 (11.1)	0 (0)	4 (44.4)	3 (33.3)	2 (22.2)	6 (75.0)	2 (25.0)	0 (0)	2 (22.2)	4 (44.4)	3 (33.3)
Chinese Greens	Adverse	31 (93.9)	2 (6.1)	0 (0)	10 (30.3)	18 (54.6)	5 (15.2)	14 (42.4)	14 (42.4)	5 (15.2)	6 (20.7)	18 (62.1)	5 (17.2)
Rocket	No Difference	148 (94.9)	8 (5.1)	0 (0)	58 (37.4)	78 (50.3)	19 (12.3)	95 (60.5)	53 (33.8)	9 (5.7)	45 (30.6)	74 (50.3)	28 (19.1)
Rocket	Beneficial	4 (80.0)	1 (20.0)	0 (0)	2 (40.0)	3 (60.0)	0 (0)	1 (20.0)	2 (40.0)	2 (40.0)	1 (20.0)	2 (40.0)	2 (40.0)
Rocket	Adverse	21 (87.5)	3 (12.5)	0 (0)	7 (28.0)	16 (64.0)	2 (8.0)	14 (58.3)	9 (37.5)	1 (4.2)	7 (29.2)	12 (50.0)	5 (20.8)
Watercress	No Difference	72 (100)	0 (0)	0 (0)	28 (38.4)	38 (52.1)	7 (9.6)	43 (58.9)	26 (35.6)	4 (5.5)	22 (31.4)	35 (50.0)	13 (18.6)
Watercress	Beneficial	8 (88.9)	1 (11.1)	0 (0)	3 (33.3)	4 (44.4)	2 (22.2)	4 (50.0)	4 (50.0)	0 (0)	2 (25.0)	5 (62.5)	1 (12.5)
Watercress	Adverse	16 (94.1)	1 (5.9)	0 (0)	6 (35.3)	9 (52.9)	2 (11.8)	14 (82.4)	2 (11.8)	1 (5.9)	8 (53.3)	5 (33.3)	2 (13.3)

Table S1. *Cont.*

		DIO1	rs7515322		DIO2	rs12885300		DIO2	rs2250114		DIO3	rs1190715	
		A/A	A/G	G/G	C/C	C/T	T/T	A/A	A/G	G/G	C/C	C/T	T/T
Horseradish	No Difference	84 (94.4)	5 (5.6)	0 (0)	40 (45.5)	42 (47.7)	6 (6.8)	57 (63.3)	30 (33.3)	3 (3.3)	24 (27.9)	42 (48.8)	20 (23.3)
Horseradish	Beneficial	2 (100)	0 (0)	0 (0)	0 (0)	1 (50.0)	1 (50.0)	1 (50.0)	0 (0)	1 (50.0)	0 (0)	0 (0)	2 (100)
Horseradish	Adverse	45 (93.8)	3 (6.3)	0 (0)	15 (31.3)	24 (50.0)	9 (18.8)	24 (51.1)	19 (40.4)	4 (8.5)	17 (38.6)	23 (52.3)	4 (9.1)
Mustard powder	No Difference	154 (95.1)	8 (4.9)	0 (0)	67 (41.4)	78 (48.2)	17 (10.5)	100 (61.7)	53 (32.7)	9 (5.6)	52 (33.8)	70 (45.5)	32 (20.8)
Mustard powder	Beneficial	2 (66.7)	1 (33.3)	0 (0)	1 (33.3)	2 (66.7)	0 (0)	1 (33.3)	0 (0)	2 (66.7)	1 (33.3)	1 (33.3)	1 (33.3)
Mustard powder	Adverse	50 (90.9)	5 (9.1)	0 (0)	18 (32.7)	26 (47.3)	11 (20.0)	35 (62.5)	17 (30.4)	4 (7.1)	12 (23.1)	31 (59.6)	9 (17.3)
Mustard sauce	No Difference	143 (95.3)	7 (4.7)	0 (0)	61 (40.7)	72 (48.0)	17 (11.3)	97 (65.1)	44 (29.5)	8 (5.4)	43 (29.5)	72 (49.3)	31 (21.2)
Mustard sauce	Beneficial	4 (100)	0 (0)	0 (0)	3 (75.0)	1 (25.0)	0 (0)	0 (0)	3 (75.0)	1 (25.0)	0 (0)	2 (50.0)	2 (50.0)
Mustard sauce	Adverse	63 (94)	4 (6.0)	0 (0)	23 (34.3)	33 (49.3)	11 (16.4)	40 (60.6)	20 (30.3)	6 (9.1)	17 (27.9)	35 (57.4)	9 (14.8)
Wasabi	No Difference	76 (95)	4 (5)	0 (0)	32 (40.0)	39 (48.8)	9 (11.3)	55 (68.8)	23 (28.8)	2 (2.5)	17 (22.1)	43 (55.8)	17 (22.1)
Wasabi	Beneficial	3 (100)	0 (0)	0 (0)	2 (66.7)	1 (33.3)	0 (0)	0 (0)	2 (66.7)	1 (33.3)	0 (0)	1 (50.0)	1 (50.0)
Wasabi	Adverse	57 (98.3)	1 (1.7)	0 (0)	18 (30.5)	33 (55.9)	8 (13.6)	30 (51.7)	23 (39.7)	5 (8.6)	23 (41.1)	29 (51.8)	4 (7.1)

Table S1. *Cont.*

		**DIO3**	**rs1190716**		**DIO3**	**rs945006**		**DLG5**	**rs2289311**		**FAM92B**	**rs8050910**	
		C/C	C/T	T/T	G/G	G/T	T/T	C/C	C/T	T/T	G/G	G/T	T/T
Broccoli	No Difference	109 (80.2)	25 (18.4)	2 (1.5)	3 (1.6)	42 (23.0)	138 (75.4)	84 (42.4)	98 (49.5)	16 (8.1)	5 (11.4)	31 (70.5)	8 (18.2)
Broccoli	Beneficial	12 (75.0)	3 (18.8)	1 (6.3)	0 (0)	3 (14.3)	18 (85.7)	11 (45.8)	10 (41.7)	3 (12.5)	2 (25.0)	3 (37.5)	3 (37.5)
Broccoli	Adverse	34 (89.5)	4 (10.5)	0 (0)	1 (1.9)	9 (17.3)	42 (80.8)	23 (41.8)	24 (43.6)	8 (14.6)	2 (25.0)	5 (62.5)	1 (12.5)
Cabbage	No Difference	75 (82.4)	16 (17.6)	0 (0)	0 (0)	26 (20.8)	99 (79.2)	55 (39.9)	71 (51.5)	12 (8.7)	1 (3.7)	20 (74.1)	6 (22.2)
Cabbage	Beneficial	4 (66.7)	2 (33.3)	0 (0)	0 (0)	2 (28.6)	5 (71.4)	2 (33.3)	2 (33.3)	2 (33.3)	0 (0)	1 (50.0)	1 (50.0)
Cabbage	Adverse	77 (78.6)	18 (18.4)	3 (3.1)	3 (2.4)	28 (22.2)	95 (75.4)	65 (48.9)	55 (41.4)	13 (9.8)	7 (24.1)	17 (58.6)	5 (17.2)
Cauliflower	No Difference	108 (80.6)	25 (18.7)	1 (0.8)	1 (0.6)	41 (22.9)	137 (76.5)	81 (42)	94 (48.7)	18 (9.3)	4 (10.0)	29 (72.5)	7 (17.5)
Cauliflower	Beneficial	11 (84.6)	1 (7.7)	1 (7.7)	0 (0)	2 (11.1)	16 (88.9)	10 (50.0)	7 (35.0)	3 (15)	2 (28.6)	2 (28.6)	3 (42.9)
Cauliflower	Adverse	36 (83.7)	6 (14.0)	1 (2.3)	3 (4.8)	13 (20.6)	47 (74.6)	28 (40.6)	35 (50.7)	6 (8.7)	2 (16.7)	8 (66.7)	2 (16.7)
Chinese Greens	No Difference	67 (79.8)	15 (17.9)	2 (2.4)	3 (2.7)	22 (19.8)	86 (77.5)	49 (39.5)	67 (54)	8 (6.5)	2 (8.7)	17 (73.9)	4 (17.4)
Chinese Greens	Beneficial	6 (75.0)	1 (12.5)	1 (12.5)	0 (0)	1 (11.1)	8 (88.9)	4 (50.0)	3 (37.5)	1 (12.5)	0 (0)	2 (50.0)	2 (50.0)
Chinese Greens	Adverse	21 (87.5)	3 (12.5)	0 (0)	0 (0)	9 (27.3)	24 (72.7)	15 (41.7)	16 (44.4)	5 (13.9)	2 (66.7)	1 (33.3)	0 (0)
Rocket	No Difference	92 (81.4)	19 (16.8)	2 (1.8)	3 (1.9)	32 (20.7)	120 (77.4)	69 (40.1)	85 (49.4)	18 (10.5)	7 (18.4)	25 (65.8)	6 (15.8)
Rocket	Beneficial	3 (100)	0 (0)	0 (0)	1 (20.0)	1 (20.0)	3 (60.0)	2 (40.0)	3 (60.0)	0 (0)	0 (0)	0 (0)	1 (100)
Rocket	Adverse	15 (75.0)	5 (25.0)	0 (0)	0 (0)	7 (29.2)	17 (70.8)	13 (44.8)	11 (37.9)	5 (17.2)	1 (50.0)	1 (50.0)	0 (0)
Watercress	No Difference	42 (77.8)	12 (22.2)	0 (0)	0 (0)	20 (27.4)	53 (72.6)	26 (31.7)	45 (54.9)	11 (13.4)	3 (18.8)	9 (56.3)	4 (25.0)
Watercress	Beneficial	6 (85.7)	1 (14.3)	0 (0)	0 (0)	2 (22.2)	7 (77.8)	3 (37.5)	3 (37.5)	2 (25.0)	1 (50.0)	0 (0)	1 (50.0)

Table S1. *Cont.*

		**DIO3**	**rs1190716**		**DIO3**	**rs945006**		**DLG5**	**rs2289311**		**FAM92B**	**rs8050910**	
		C/C	C/T	T/T	G/G	G/T	T/T	C/C	C/T	T/T	G/G	G/T	T/T
Watercress	Adverse	7 (63.6)	2 (18.2)	2 (18.2)	0 (0)	4 (25.0)	12 (75.0)	11 (55.0)	7 (35.0)	2 (10.0)	1 (33.3)	2 (66.7)	0 (0)
Horseradish	No Difference	55 (83.3)	10 (15.2)	1 (1.5)	0 (0)	18 (20.5)	70 (79.6)	38 (39.2)	50 (51.6)	9 (9.3)	3 (18.8)	9 (56.3)	4 (25.0)
Horseradish	Beneficial	2 (100)	0 (0)	0 (0)	0 (0)	0 (0)	2 (100)	1 (50.0)	1 (50.0)	0 (0)	0 (0)	1 (100)	0 (0)
Horseradish	Adverse	29 (78.4)	6 (16.2)	2 (5.4)	2 (4.3)	11 (23.4)	34 (72.3)	24 (50.0)	20 (41.7)	4 (8.3)	2 (25.0)	4 (50.0)	2 (25.0)
Mustard powder	No Difference	98 (82.4)	20 (16.8)	1 (0.8)	3 (1.8)	30 (18.4)	130 (79.8)	76 (44.2)	82 (47.7)	14 (8.1)	4 (12.1)	24 (72.7)	5 (15.2)
Mustard powder	Beneficial	2 (100)	0 (0)	0 (0)	1 (33.3)	0 (0)	2 (66.7)	1 (33.3)	2 (66.7)	0 (0)	0 (0)	0 (0)	0 (0)
Mustard powder	Adverse	31 (75.6)	9 (22)	1 (2.4)	0 (0)	17 (32.1)	36 (67.9)	22 (36.7)	29 (48.3)	9 (15)	3 (21.4)	8 (57.1)	3 (21.4)
Mustard sauce	No Difference	94 (82.5)	19 (16.7)	1 (0.9)	2 (1.3)	30 (19.9)	119 (78.8)	65 (40.4)	81 (50.3)	15 (9.3)	3 (10.7)	20 (71.4)	5 (17.9)
Mustard sauce	Beneficial	4 (100)	0 (0)	0 (0)	0 (0)	0 (0)	4 (100)	2 (50.0)	1 (25.0)	1 (25.0)	0 (0)	0 (0)	1 (100)
Mustard sauce	Adverse	38 (77.6)	9 (18.4)	2 (4.1)	1 (1.5)	18 (27.7)	46 (70.8)	29 (40.9)	33 (46.5)	9 (12.7)	2 (14.3)	11 (78.6)	1 (7.1)
Wasabi	No Difference	52 (86.7)	8 (13.3)	0 (0)	0 (0)	12 (15)	68 (85)	41 (46.1)	40 (44.9)	8 (9)	3 (20.0)	9 (60.0)	3 (20.0)
Wasabi	Beneficial	3 (100)	0 (0)	0 (0)	0 (0)	0 (0)	3 (100)	1 (33.3)	1 (33.3)	1 (33.3)	1 (100)	0 (0)	0 (0)
Wasabi	Adverse	30 (71.4)	9 (21.4)	3 (7.1)	2 (3.5)	18 (31.6)	37 (64.9)	23 (35.4)	35 (53.9)	7 (10.8)	1 (8.3)	7 (58.3)	4 (33.3)

Table S1. *Cont.*

		**GPX1**	**rs1800668**		**GPX2**	**rs1800669**		**GPX2**	**rs2296327**		**GPX2**	**rs2412065**	
		C/C	C/T	T/T	A/A	A/T	T/T	C/C	C/T	T/T	C/C	C/G	G/G
Broccoli	No Difference	28 (38.9)	35 (48.6)	9 (12.5)	178 (96.2)	7 (3.8)	0 (0)	115 (63.2)	55 (30.2)	12 (6.6)	114 (64.8)	56 (31.8)	6 (3.4)
Broccoli	Beneficial	2 (18.2)	6 (54.6)	3 (27.3)	20 (90.9)	2 (9.1)	0 (0)	13 (61.9)	8 (38.1)	0 (0)	14 (63.6)	8 (36.4)	0 (0)
Broccoli	Adverse	4 (33.3)	7 (58.3)	1 (8.3)	48 (92.3)	4 (7.7)	0 (0)	34 (66.7)	15 (29.4)	2 (3.9)	30 (58.8)	20 (39.2)	1 (2)
Cabbage	No Difference	23 (42.6)	25 (46.3)	6 (11.1)	123 (98.4)	2 (1.6)	0 (0)	76 (62.8)	36 (29.8)	9 (7.4)	72 (62.1)	40 (34.5)	4 (3.5)
Cabbage	Beneficial	2 (50.0)	1 (25.0)	1 (25.0)	7 (100)	0 (0)	0 (0)	6 (85.7)	1 (14.3)	0 (0)	6 (85.7)	1 (14.3)	0 (0)
Cabbage	Adverse	13 (29.6)	23 (52.3)	8 (18.2)	119 (92.3)	10 (7.8)	0 (0)	80 (63.5)	41 (32.5)	5 (4.0)	77 (61.1)	46 (36.5)	3 (2.4)
Cauliflower	No Difference	28 (41.8)	31 (46.3)	8 (11.9)	174 (96.1)	7 (3.9)	0 (0)	113 (63.8)	54 (30.5)	10 (5.7)	113 (65.7)	54 (31.4)	5 (2.9)
Cauliflower	Beneficial	2 (22.2)	5 (55.6)	2 (22.2)	18 (94.7)	1 (5.3)	0 (0)	12 (66.7)	6 (33.3)	0 (0)	12 (63.2)	7 (36.8)	0 (0)
Cauliflower	Adverse	7 (31.8)	11 (50.0)	4 (18.2)	58 (92.1)	5 (7.9)	0 (0)	42 (67.7)	16 (25.8)	4 (6.5)	36 (59)	23 (37.7)	2 (3.3)
Chinese Greens	No Difference	18 (42.9)	21 (50.0)	3 (7.1)	109 (97.3)	3 (2.7)	0 (0)	65 (59.6)	39 (35.8)	5 (4.6)	69 (63.3)	38 (34.9)	2 (1.8)
Chinese Greens	Beneficial	0 (0)	1 (50.0)	1 (50.0)	9 (100)	0 (0)	0 (0)	6 (66.7)	3 (33.3)	0 (0)	5 (55.6)	4 (44.4)	0 (0)
Chinese Greens	Adverse	3 (18.8)	9 (56.3)	4 (25.0)	31 (93.9)	2 (6.1)	0 (0)	20 (62.5)	12 (37.5)	0 (0)	20 (62.5)	12 (37.5)	0 (0)
Rocket	No Difference	22 (37.3)	28 (47.5)	9 (15.3)	149 (95.5)	7 (4.5)	0 (0)	92 (60.5)	54 (35.5)	6 (4.0)	98 (65.3)	49 (32.7)	3 (2)
Rocket	Beneficial	1 (50.0)	1 (50.0)	0 (0)	5 (100)	0 (0)	0 (0)	4 (80.0)	1 (20.0)	0 (0)	4 (80.0)	1 (20.0)	0 (0)
Rocket	Adverse	0 (0)	6 (85.7)	1 (14.3)	24 (96)	1 (4.0)	0 (0)	19 (79.2)	4 (16.7)	1 (4.2)	16 (66.7)	7 (29.2)	1 (4.2)
Watercress	No Difference	11 (40.7)	13 (48.2)	3 (11.1)	72 (98.6)	1 (1.4)	0 (0)	44 (61.1)	24 (33.3)	4 (5.6)	50 (69.4)	20 (27.8)	2 (2.8)
Watercress	Beneficial	1 (20.0)	3 (60.0)	1 (20.0)	9 (100)	0 (0)	0 (0)	6 (66.7)	3 (33.3)	0 (0)	6 (66.7)	3 (33.3)	0 (0)
Watercress	Adverse	1 (14.3)	6 (85.7)	0 (0)	17 (100)	0 (0)	0 (0)	9 (56.3)	7 (43.8)	0 (0)	9 (52.9)	8 (47.1)	0 (0)
Horseradish	No Difference	11 (34.4)	19 (59.4)	2 (6.3)	87 (97.8)	2 (2.3)	0 (0)	50 (57.5)	32 (36.8)	5 (5.8)	49 (57.0)	34 (39.5)	3 (3.5)
Horseradish	Beneficial	1 (100)	0 (0)	0 (0)	2 (100)	0 (0)	0 (0)	2 (100)	0 (0)	0 (0)	2 (100)	0 (0)	0 (0)
Horseradish	Adverse	8 (40.0)	10 (50.0)	2 (10.0)	45 (93.8)	3 (6.3)	0 (0)	30 (63.8)	15 (31.9)	2 (4.3)	33 (68.8)	15 (31.3)	0 (0)
Mustard powder	No Difference	27 (43.6)	25 (40.3)	10 (16.1)	159 (97.6)	4 (2.5)	0 (0)	105 (66.5)	44 (27.9)	9 (5.7)	93 (60.4)	55 (35.7)	6 (3.9)
Mustard powder	Beneficial	1 (50.0)	1 (50.0)	0 (0)	3 (100)	0 (0)	0 (0)	3 (100)	0 (0)	0 (0)	3 (100)	0 (0)	0 (0)
Mustard powder	Adverse	5 (21.7)	14 (60.9)	4 (17.4)	51 (92.7)	4 (7.3)	0 (0)	33 (61.1)	20 (37)	1 (1.9)	40 (75.5)	13 (24.5)	0 (0)
Mustard sauce	No Difference	21 (36.8)	27 (47.4)	9 (15.8)	146 (96.7)	5 (3.3)	0 (0)	95 (64.6)	43 (29.3)	9 (6.1)	84 (58.3)	54 (37.5)	6 (4.2)
Mustard sauce	Beneficial	3 (100)	0 (0)	0 (0)	4 (100)	0 (0)	0 (0)	3 (75.0)	1 (25.0)	0 (0)	3 (75.0)	1 (25.0)	0 (0)
Mustard sauce	Adverse	9 (29)	17 (54.8)	5 (16.1)	61 (91)	6 (9)	0 (0)	41 (62.1)	24 (36.4)	1 (1.5)	50 (75.8)	16 (24.2)	0 (0)
Wasabi	No Difference	12 (44.4)	10 (37)	5 (18.5)	78 (97.5)	2 (2.5)	0 (0)	47 (58.8)	28 (35.0)	5 (6.3)	44 (56.4)	32 (41)	2 (2.6)
Wasabi	Beneficial	2 (66.7)	1 (33.3)	0 (0)	3 (100)	0 (0)	0 (0)	2 (66.7)	1 (33.3)	0 (0)	2 (66.7)	1 (33.3)	0 (0)
Wasabi	Adverse	8 (28.6)	18 (64.3)	2 (7.1)	57 (96.6)	2 (3.4)	0 (0)	40 (69)	18 (31)	0 (0)	43 (74.1)	15 (25.9)	0 (0)

Table S1. *Cont.*

		**GPX2**	**rs2737844**		**GPX2**	**rs3742599**		**GPX3**	**rs2042235**		**GPX3**	**rs3763013**	
		A/A	A/G	G/G	A/A	A/C	C/C	C/C	C/T	T/T	C/C	C/T	T/T
Broccoli	No Difference	13 (7.5)	67 (38.5)	94 (54)	5 (2.9)	42 (24.1)	127 (73)	135 (67.2)	61 (30.4)	5 (2.5)	20 (11)	88 (48.4)	74 (40.7)
Broccoli	Beneficial	0 (0)	7 (36.8)	12 (63.2)	0 (0)	7 (35.0)	13 (65)	19 (73.1)	7 (26.9)	0 (0)	0 (0)	7 (31.8)	15 (68.2)
Broccoli	Adverse	3 (6.3)	24 (50.0)	21 (43.8)	1 (2)	16 (31.4)	34 (66.7)	36 (62.1)	18 (31)	4 (6.9)	7 (14.0)	20 (40.0)	23 (46.0)
Cabbage	No Difference	10 (8.7)	45 (39.1)	60 (52.2)	4 (3.4)	32 (27.4)	81 (69.2)	91 (65)	45 (32.1)	4 (2.9)	8 (6.5)	62 (50.4)	53 (43.1)
Cabbage	Beneficial	0 (0)	1 (16.7)	5 (83.3)	0 (0)	0 (0)	7 (100)	6 (85.7)	1 (14.3)	0 (0)	0 (0)	1 (16.7)	5 (83.3)
Cabbage	Adverse	6 (5.1)	52 (44.1)	60 (50.9)	2 (1.6)	34 (27.4)	88 (71)	97 (70.3)	37 (26.8)	4 (2.9)	17 (13.5)	46 (36.5)	63 (50.0)
Cauliflower	No Difference	11 (6.6)	63 (37.5)	94 (56.0)	4 (2.4)	43 (25.3)	123 (72.4)	133 (67.5)	58 (29.4)	6 (3.1)	19 (10.6)	87 (48.6)	73 (40.8)
Cauliflower	Beneficial	0 (0)	5 (31.3)	11 (68.8)	0 (0)	6 (35.3)	11 (64.7)	17 (77.3)	5 (22.7)	0 (0)	0 (0)	4 (21.1)	15 (79)
Cauliflower	Adverse	5 (8.5)	29 (49.2)	25 (42.4)	2 (3.2)	16 (25.4)	45 (71.4)	43 (60.6)	23 (32.4)	5 (7.0)	10 (16.7)	21 (35.0)	29 (48.3)
Chinese Greens	No Difference	9 (8.5)	44 (41.5)	53 (50.0)	2 (1.9)	29 (26.9)	77 (71.3)	92 (71.9)	31 (24.2)	5 (3.9)	9 (8.3)	45 (41.3)	55 (50.5)
Chinese Greens	Beneficial	0 (0)	4 (50.0)	4 (50.0)	0 (0)	3 (33.3)	6 (66.7)	8 (88.9)	1 (11.1)	0 (0)	0 (0)	2 (25.0)	6 (75.0)
Chinese Greens	Adverse	0 (0)	13 (41.9)	18 (58.1)	0 (0)	9 (30.0)	21 (70.0)	26 (68.4)	12 (31.6)	0 (0)	3 (9.4)	13 (40.6)	16 (50.0)
Rocket	No Difference	10 (6.8)	58 (39.5)	79 (53.7)	3 (2)	37 (24.7)	110 (73.3)	123 (69.9)	49 (27.8)	4 (2.3)	12 (7.7)	74 (47.4)	70 (44.9)
Rocket	Beneficial	0 (0)	1 (20.0)	4 (80.0)	0 (0)	1 (20.0)	4 (80.0)	5 (83.3)	1 (16.7)	0 (0)	0 (0)	1 (20.0)	4 (80.0)
Rocket	Adverse	2 (9.5)	10 (47.6)	9 (42.9)	1 (4.2)	5 (20.8)	18 (75.0)	15 (51.7)	11 (37.9)	3 (10.3)	6 (25.0)	6 (25.0)	12 (50.0)
Watercress	No Difference	5 (7.1)	25 (35.7)	40 (57.1)	2 (2.9)	17 (24.3)	51 (72.9)	57 (65.5)	28 (32.2)	2 (2.3)	6 (8.3)	33 (45.8)	33 (45.8)
Watercress	Beneficial	0 (0)	3 (33.3)	6 (66.7)	0 (0)	3 (37.5)	5 (62.5)	8 (80.0)	2 (20.0)	0 (0)	0 (0)	2 (25.0)	6 (75.0)

Table S1. *Cont.*

		**GPX2**	**rs2737844**		**GPX2**	**rs3742599**		**GPX3**	**rs2042235**		**GPX3**	**rs3763013**	
		A/A	A/G	G/G	A/A	A/C	C/C	C/C	C/T	T/T	C/C	C/T	T/T
Watercress	Adverse	0 (0)	6 (42.9)	8 (57.1)	0 (0)	5 (33.3)	10 (66.7)	12 (60.0)	7 (35.0)	1 (5)	2 (12.5)	8 (50.0)	6 (37.5)
Horseradish	No Difference	6 (7.1)	37 (43.5)	42 (49.4)	3 (3.5)	27 (31)	57 (65.5)	73 (72.3)	25 (24.8)	3 (3)	7 (7.9)	41 (46.1)	41 (46.1)
Horseradish	Beneficial	0 (0)	1 (50.0)	1 (50.0)	0 (0)	0 (0)	2 (100)	2 (100)	0 (0)	0 (0)	0 (0)	0 (0)	2 (100)
Horseradish	Adverse	2 (4.4)	18 (40.0)	25 (55.6)	0 (0)	12 (26.7)	33 (73.3)	30 (61.2)	19 (38.8)	0 (0)	5 (10.9)	17 (37)	24 (52.2)
Mustard powder	No Difference	12 (7.8)	64 (41.8)	77 (50.3)	6 (3.8)	42 (26.8)	109 (69.4)	114 (64.4)	58 (32.8)	5 (2.8)	17 (10.5)	80 (49.4)	65 (40.1)
Mustard powder	Beneficial	0 (0)	0 (0)	3 (100)	0 (0)	0 (0)	3 (100)	3 (100)	0 (0)	0 (0)	0 (0)	1 (33.3)	2 (66.7)
Mustard powder	Adverse	0 (0)	19 (38.0)	31 (62)	0 (0)	11 (21.2)	41 (78.9)	43 (70.5)	18 (29.5)	0 (0)	4 (7.3)	21 (38.2)	30 (54.6)
Mustard sauce	No Difference	12 (8.5)	57 (40.1)	73 (51.4)	6 (4.1)	41 (27.7)	101 (68.2)	108 (65.5)	51 (30.9)	6 (3.6)	16 (10.8)	71 (48.0)	61 (41.2)
Mustard sauce	Beneficial	0 (0)	1 (25.0)	3 (75.0)	0 (0)	1 (25.0)	3 (75.0)	3 (75.0)	0 (0)	1 (25.0)	1 (25.0)	0 (0)	3 (75.0)
Mustard sauce	Adverse	2 (3.3)	22 (36.1)	37 (60.7)	0 (0)	13 (21)	49 (79)	49 (68.1)	22 (30.6)	1 (1.4)	6 (9.2)	26 (40.0)	33 (50.8)
Wasabi	No Difference	6 (8.0)	34 (45.3)	35 (46.7)	2 (2.6)	23 (29.9)	52 (67.5)	65 (71.4)	24 (26.4)	2 (2.2)	8 (10.0)	35 (43.8)	37 (46.3)
Wasabi	Beneficial	0 (0)	1 (33.3)	2 (66.7)	0 (0)	1 (50.0)	1 (50.0)	2 (66.7)	0 (0)	1 (33.3)	1 (33.3)	0 (0)	2 (66.7)
Wasabi	Adverse	2 (3.9)	17 (32.7)	33 (63.5)	0 (0)	12 (21.1)	45 (79)	42 (61.8)	23 (33.8)	3 (4.4)	7 (12.5)	24 (42.9)	25 (44.6)

Table S1. *Cont.*

		**GPX3**	**rs3792796**		**GPX3**	**rs3792797**		**GPX3**	**rs3805435**		**GPX3**	**rs3828599**	
		C/C	C/G	G/G	A/A	A/C	C/C	A/A	A/G	G/G	A/A	A/G	G/G
Broccoli	No Difference	70 (41.7)	75 (44.6)	23 (13.7)	5 (2.8)	50 (27.8)	125 (69.4)	153 (83.6)	23 (12.6)	7 (3.8)	11 (6.0)	63 (34.4)	109 (59.6)
Broccoli	Beneficial	13 (65)	7 (35.0)	0 (0)	0 (0)	4 (18.2)	18 (81.8)	17 (85)	3 (15)	0 (0)	0 (0)	6 (27.3)	16 (72.7)
Broccoli	Adverse	20 (39.2)	23 (45.1)	8 (15.7)	1 (1.9)	14 (26.9)	37 (71.2)	38 (74.5)	13 (25.5)	0 (0)	2 (3.9)	20 (39.2)	29 (56.9)
Cabbage	No Difference	45 (39.8)	55 (48.7)	13 (11.5)	5 (4.1)	35 (28.5)	83 (67.5)	105 (83.3)	16 (12.7)	5 (4.0)	10 (8.0)	47 (37.6)	68 (54.4)
Cabbage	Beneficial	4 (57.1)	2 (28.6)	1 (14.3)	0 (0)	0 (0)	6 (100)	4 (57.1)	3 (42.9)	0 (0)	0 (0)	2 (33.3)	4 (66.7)
Cabbage	Adverse	57 (46.7)	47 (38.5)	18 (14.8)	2 (1.6)	33 (26.2)	91 (72.2)	102 (81.6)	20 (16.0)	3 (2.4)	4 (3.2)	43 (34.1)	79 (62.7)
Cauliflower	No Difference	67 (40.4)	78 (47)	21 (12.7)	7 (4.0)	48 (27.1)	122 (68.9)	150 (83.8)	23 (12.9)	6 (3.4)	13 (7.2)	60 (33.3)	107 (59.4)
Cauliflower	Beneficial	13 (76.5)	4 (23.5)	0 (0)	0 (0)	2 (10.5)	17 (89.5)	14 (82.4)	3 (17.7)	0 (0)	0 (0)	4 (21.1)	15 (79)
Cauliflower	Adverse	27 (44.3)	23 (37.7)	11 (18)	2 (3.2)	16 (25.8)	44 (71)	48 (77.4)	13 (21)	1 (1.6)	3 (4.9)	23 (37.7)	35 (57.4)
Chinese Greens	No Difference	48 (45.7)	42 (40.0)	15 (14.3)	5 (4.6)	22 (20.4)	81 (75.0)	93 (82.3)	17 (15)	3 (2.7)	9 (8.2)	34 (30.9)	67 (60.9)
Chinese Greens	Beneficial	5 (55.6)	2 (22.2)	2 (22.2)	0 (0)	2 (25.0)	6 (75.0)	7 (87.5)	1 (12.5)	0 (0)	0 (0)	3 (37.5)	5 (62.5)
Chinese Greens	Adverse	10 (35.7)	15 (53.6)	3 (10.7)	0 (0)	11 (33.3)	22 (66.7)	31 (91.2)	3 (8.8)	0 (0)	1 (3)	13 (39.4)	19 (57.6)
Rocket	No Difference	63 (43.8)	69 (47.9)	12 (8.3)	3 (2)	38 (24.7)	113 (73.4)	129 (82.2)	23 (14.7)	5 (3.2)	6 (3.9)	54 (34.6)	96 (61.5)
Rocket	Beneficial	3 (60.0)	1 (20.0)	1 (20.0)	0 (0)	0 (0)	5 (100)	3 (60.0)	2 (40.0)	0 (0)	0 (0)	2 (40.0)	3 (60.0)

Table S1. *Cont.*

		GPX3	rs3792796		GPX3	rs3792797		GPX3	rs3805435		GPX3	rs3828599	
		C/C	C/G	G/G	A/A	A/C	C/C	A/A	A/G	G/G	A/A	A/G	G/G
Rocket	Adverse	9 (37.5)	9 (37.5)	6 (25.0)	3 (12.5)	7 (29.2)	14 (58.3)	21 (84)	4 (16.0)	0 (0)	4 (16.7)	8 (33.3)	12 (50.0)
Watercress	No Difference	31 (44.3)	30 (42.9)	9 (12.9)	3 (4.2)	17 (23.6)	52 (72.2)	62 (83.8)	9 (12.2)	3 (4.1)	5 (6.9)	26 (35.6)	42 (57.5)
Watercress	Beneficial	4 (57.1)	2 (28.6)	1 (14.3)	0 (0)	1 (12.5)	7 (87.5)	6 (75.0)	2 (25.0)	0 (0)	0 (0)	2 (25.0)	6 (75.0)
Watercress	Adverse	5 (33.3)	7 (46.7)	3 (20.0)	1 (6.3)	5 (31.3)	10 (62.5)	14 (82.4)	3 (17.7)	0 (0)	1 (5.9)	8 (47.1)	8 (47.1)
Horseradish	No Difference	34 (41)	39 (47)	10 (12.1)	2 (2.3)	24 (27.6)	61 (70.1)	75 (83.3)	12 (13.3)	3 (3.3)	4 (4.4)	36 (40.0)	50 (55.6)
Horseradish	Beneficial	1 (50.0)	1 (50.0)	0 (0)	0 (0)	1 (50.0)	1 (50.0)	2 (100)	0 (0)	0 (0)	0 (0)	1 (50.0)	1 (50.0)
Horseradish	Adverse	19 (43.2)	18 (40.9)	7 (15.9)	0 (0)	14 (30.4)	32 (69.6)	37 (78.7)	9 (19.2)	1 (2.1)	1 (2.1)	22 (46.8)	24 (51.1)
Mustard powder	No Difference	58 (38.2)	71 (46.7)	23 (15.1)	5 (3.1)	42 (26.3)	113 (70.6)	130 (80.8)	23 (14.3)	8 (5)	10 (6.2)	61 (37.7)	91 (56.2)
Mustard powder	Beneficial	2 (66.7)	0 (0)	1 (33.3)	0 (0)	0 (0)	3 (100)	2 (66.7)	1 (33.3)	0 (0)	0 (0)	1 (33.3)	2 (66.7)
Mustard powder	Adverse	27 (52.9)	22 (43.1)	2 (3.9)	0 (0)	13 (24.1)	41 (75.9)	44 (80.0)	11 (20.0)	0 (0)	1 (1.8)	16 (29.1)	38 (69.1)
Mustard sauce	No Difference	57 (39.9)	63 (44.1)	23 (16.1)	6 (4.1)	35 (23.8)	106 (72.1)	126 (84)	18 (12.0)	6 (4.0)	10 (6.7)	50 (33.6)	89 (59.7)
Mustard sauce	Beneficial	3 (75.0)	1 (25.0)	0 (0)	0 (0)	1 (25.0)	3 (75.0)	3 (75.0)	1 (25.0)	0 (0)	0 (0)	1 (25.0)	3 (75.0)
Mustard sauce	Adverse	29 (47.5)	24 (39.3)	8 (13.1)	1 (1.6)	18 (28.1)	45 (70.3)	51 (78.5)	14 (21.5)	0 (0)	2 (3.1)	24 (36.9)	39 (60.0)
Wasabi	No Difference	30 (39.5)	36 (47.4)	10 (13.2)	2 (2.5)	20 (25.3)	57 (72.2)	65 (82.3)	11 (13.9)	3 (3.8)	4 (5)	30 (37.5)	46 (57.5)
Wasabi	Beneficial	1 (50.0)	1 (50.0)	0 (0)	0 (0)	1 (33.3)	2 (66.7)	2 (66.7)	1 (33.3)	0 (0)	0 (0)	1 (33.3)	2 (66.7)
Wasabi	Adverse	22 (39.3)	26 (46.4)	8 (14.3)	3 (5.4)	16 (28.6)	37 (66.1)	47 (78.3)	11 (18.3)	2 (3.3)	3 (5.3)	25 (43.9)	29 (50.9)

Table S1. *Cont.*

		**GPX3**	**rs8177425**		**GPX3**	**rs870407**		**HLA**	**rs9469220**		**IBD5**	**rs10077785**	
		C/C	C/T	T/T	A/A	A/G	G/G	A/A	A/G	G/G	C/C	C/T	T/T
Broccoli	No Difference	177 (96.7)	6 (3.3)	0 (0)	122 (71.4)	45 (26.3)	4 (2.3)	53 (29)	87 (47.5)	43 (23.5)	112 (62.6)	64 (35.8)	3 (1.7)
Broccoli	Beneficial	22 (100)	0 (0)	0 (0)	19 (95)	1 (5)	0 (0)	7 (35.0)	11 (55.0)	2 (10.0)	16 (72.7)	6 (27.3)	0 (0)
Broccoli	Adverse	48 (92.3)	4 (7.7)	0 (0)	38 (74.5)	13 (25.5)	0 (0)	16 (31.4)	25 (49)	10 (19.6)	36 (72)	13 (26)	1 (2)
Cabbage	No Difference	121 (97.6)	3 (2.4)	0 (0)	88 (77.2)	24 (21.1)	2 (1.8)	30 (24.6)	63 (51.6)	29 (23.8)	82 (67.2)	38 (31.2)	2 (1.6)
Cabbage	Beneficial	7 (100)	0 (0)	0 (0)	6 (85.7)	0 (0)	1 (14.3)	3 (50.0)	2 (33.3)	1 (16.7)	4 (57.1)	3 (42.9)	0 (0)
Cabbage	Adverse	122 (95.3)	6 (4.7)	0 (0)	92 (74.2)	30 (24.2)	2 (1.6)	40 (31.8)	59 (46.8)	27 (21.4)	83 (67.5)	38 (30.9)	2 (1.6)
Cauliflower	No Difference	176 (97.8)	4 (2.2)	0 (0)	118 (70.7)	45 (27)	4 (2.4)	53 (29.8)	87 (48.9)	38 (21.4)	107 (61.5)	64 (36.8)	3 (1.7)
Cauliflower	Beneficial	19 (100)	0 (0)	0 (0)	17 (100)	0 (0)	0 (0)	6 (35.3)	9 (52.9)	2 (11.8)	14 (73.7)	5 (26.3)	0 (0)
Cauliflower	Adverse	57 (90.5)	6 (9.5)	0 (0)	51 (81)	12 (19.1)	0 (0)	17 (27)	30 (47.6)	16 (25.4)	45 (72.6)	16 (25.8)	1 (1.6)
Chinese Greens	No Difference	109 (98.2)	2 (1.8)	0 (0)	79 (73.8)	28 (26.2)	0 (0)	34 (30.1)	51 (45.1)	28 (24.8)	69 (62.7)	40 (36.4)	1 (0.9)
Chinese Greens	Beneficial	9 (100)	0 (0)	0 (0)	7 (77.8)	1 (11.1)	1 (11.1)	1 (12.5)	5 (62.5)	2 (25.0)	6 (75.0)	2 (25.0)	0 (0)
Chinese Greens	Adverse	31 (93.9)	2 (6.1)	0 (0)	24 (80.0)	6 (20.0)	0 (0)	11 (34.4)	16 (50.0)	5 (15.6)	21 (63.6)	12 (36.4)	0 (0)
Rocket	No Difference	151 (97.4)	4 (2.6)	0 (0)	108 (72.5)	40 (26.9)	1 (0.7)	37 (24.0)	77 (50.0)	40 (26)	100 (64.5)	52 (33.6)	3 (1.9)
Rocket	Beneficial	5 (100)	0 (0)	0 (0)	4 (80.0)	1 (20.0)	0 (0)	1 (20.0)	3 (60.0)	1 (20.0)	3 (60.0)	2 (40.0)	0 (0)

Table S1. *Cont.*

		**GPX3**	**rs8177425**		**GPX3**	**rs870407**		**HLA**	**rs9469220**		**IBD5**	**rs10077785**	
		C/C	C/T	T/T	A/A	A/G	G/G	A/A	A/G	G/G	C/C	C/T	T/T
Rocket	Adverse	22 (91.7)	2 (8.3)	0 (0)	21 (87.5)	3 (12.5)	0 (0)	17 (70.8)	6 (25.0)	1 (4.2)	16 (72.7)	6 (27.3)	0 (0)
Watercress	No Difference	69 (95.8)	3 (4.2)	0 (0)	52 (74.3)	18 (25.7)	0 (0)	16 (21.6)	38 (51.4)	20 (27)	50 (70.4)	21 (29.6)	0 (0)
Watercress	Beneficial	9 (100)	0 (0)	0 (0)	6 (85.7)	0 (0)	1 (14.3)	2 (25.0)	5 (62.5)	1 (12.5)	6 (66.7)	3 (33.3)	0 (0)
Watercress	Adverse	17 (100)	0 (0)	0 (0)	12 (80.0)	3 (20.0)	0 (0)	3 (17.7)	8 (47.1)	6 (35.3)	10 (58.8)	7 (41.2)	0 (0)
Horseradish	No Difference	87 (98.9)	1 (1.1)	0 (0)	65 (75.6)	21 (24.4)	0 (0)	21 (23.9)	46 (52.3)	21 (23.9)	58 (66.7)	27 (31)	2 (2.3)
Horseradish	Beneficial	2 (100)	0 (0)	0 (0)	2 (100)	0 (0)	0 (0)	1 (50.0)	1 (50.0)	0 (0)	1 (100)	0 (0)	0 (0)
Horseradish	Adverse	46 (95.8)	2 (4.2)	0 (0)	34 (77.3)	10 (22.7)	0 (0)	19 (39.6)	20 (41.7)	9 (18.8)	32 (68.1)	15 (31.9)	0 (0)
Mustard powder	No Difference	155 (96.3)	6 (3.7)	0 (0)	110 (70.5)	43 (27.6)	3 (1.9)	47 (29.2)	80 (49.7)	34 (21.1)	103 (64.8)	53 (33.3)	3 (1.9)
Mustard powder	Beneficial	3 (100)	0 (0)	0 (0)	2 (66.7)	1 (33.3)	0 (0)	1 (33.3)	1 (33.3)	1 (33.3)	1 (33.3)	2 (66.7)	0 (0)
Mustard powder	Adverse	55 (100)	0 (0)	0 (0)	43 (84.3)	7 (13.7)	1 (2)	17 (32.1)	26 (49.1)	10 (18.9)	33 (64.7)	18 (35.3)	0 (0)
Mustard sauce	No Difference	142 (95.3)	7 (4.7)	0 (0)	105 (71.4)	38 (25.9)	4 (2.7)	43 (28.7)	75 (50.0)	32 (21.3)	98 (66.7)	47 (32)	2 (1.4)
Mustard sauce	Beneficial	4 (100)	0 (0)	0 (0)	4 (100)	0 (0)	0 (0)	1 (25.0)	3 (75.0)	0 (0)	3 (75.0)	0 (0)	1 (25.0)
Mustard sauce	Adverse	67 (100)	0 (0)	0 (0)	48 (78.7)	12 (19.7)	1 (1.6)	23 (35.4)	28 (43.1)	14 (21.5)	41 (64.1)	23 (35.9)	0 (0)
Wasabi	No Difference	77 (96.3)	3 (3.8)	0 (0)	52 (68.4)	24 (31.6)	0 (0)	25 (31.7)	39 (49.4)	15 (19)	51 (66.2)	25 (32.5)	1 (1.3)
Wasabi	Beneficial	3 (100)	0 (0)	0 (0)	2 (100)	0 (0)	0 (0)	2 (66.7)	1 (33.3)	0 (0)	1 (33.3)	1 (33.3)	1 (33.3)
Wasabi	Adverse	57 (98.3)	1 (1.7)	0 (0)	46 (80.7)	10 (17.5)	1 (1.8)	20 (33.9)	23 (39)	16 (27.1)	38 (64.4)	21 (35.6)	0 (0)

Table S1. *Cont.*

		ICAM1	rs1799969		IL12B	rs6887695		IL23R	rs11209026		IL23R	rs7517847	
		A/A	A/G	G/G	C/C	C/G	G/G	A/A	A/G	G/G	G/G	G/T	T/T
Broccoli	No Difference	1 (0.6)	39 (22.2)	136 (77.3)	15 (8.2)	88 (48.1)	80 (43.7)	0 (0)	10 (5.4)	174 (94.6)	17 (9.4)	103 (57.2)	60 (33.3)
Broccoli	Beneficial	2 (9.5)	1 (4.8)	18 (85.7)	2 (9.1)	9 (40.9)	11 (50.0)	0 (0)	2 (9.1)	20 (90.9)	1 (4.8)	10 (47.6)	10 (47.6)
Broccoli	Adverse	1 (2)	10 (20.0)	39 (78)	8 (16.0)	23 (46.0)	19 (38.0)	0 (0)	7 (14.0)	43 (86)	1 (2)	27 (54)	22 (44.0)
Cabbage	No Difference	0 (0)	22 (18.3)	98 (81.7)	7 (5.7)	56 (45.5)	60 (48.8)	0 (0)	8 (6.4)	117 (93.6)	16 (13.1)	69 (56.6)	37 (30.3)
Cabbage	Beneficial	2 (28.6)	0 (0)	5 (71.4)	0 (0)	3 (42.9)	4 (57.1)	0 (0)	2 (28.6)	5 (71.4)	1 (14.3)	4 (57.1)	2 (28.6)
Cabbage	Adverse	2 (1.6)	25 (20.5)	95 (77.9)	17 (13.5)	59 (46.8)	50 (39.7)	0 (0)	9 (7.1)	117 (92.9)	6 (4.8)	64 (51.6)	54 (43.6)
Cauliflower	No Difference	1 (0.6)	35 (20.4)	136 (79.1)	16 (9)	83 (46.6)	79 (44.4)	0 (0)	11 (6.2)	168 (93.9)	16 (9.1)	104 (59.4)	55 (31.4)
Cauliflower	Beneficial	2 (11.1)	0 (0)	16 (88.9)	2 (10.5)	9 (47.4)	8 (42.1)	0 (0)	2 (10.5)	17 (89.5)	1 (5.6)	9 (50.0)	8 (44.4)
Cauliflower	Adverse	1 (1.6)	16 (26.2)	44 (72.1)	8 (12.9)	30 (48.4)	24 (38.7)	0 (0)	6 (9.7)	56 (90.3)	4 (6.5)	29 (46.8)	29 (46.8)
Chinese Greens	No Difference	2 (1.8)	30 (27.3)	78 (70.9)	3 (2.7)	50 (44.6)	59 (52.7)	0 (0)	7 (6.2)	106 (93.8)	13 (11.5)	64 (56.6)	36 (31.9)
Chinese Greens	Beneficial	1 (11.1)	0 (0)	8 (88.9)	0 (0)	3 (37.5)	5 (62.5)	0 (0)	0 (0)	8 (100)	2 (22.2)	4 (44.4)	3 (33.3)
Chinese Greens	Adverse	1 (3)	4 (12.1)	28 (84.9)	6 (18.8)	16 (50.0)	10 (31.3)	0 (0)	4 (12.1)	29 (87.9)	0 (0)	15 (45.5)	18 (54.6)
Rocket	No Difference	0 (0)	30 (20.3)	118 (79.7)	11 (7.2)	71 (46.4)	71 (46.4)	0 (0)	8 (5.1)	148 (94.9)	15 (9.8)	77 (50.3)	61 (39.9)
Rocket	Beneficial	2 (40.0)	1 (20.0)	2 (40.0)	1 (20.0)	1 (20.0)	3 (60.0)	0 (0)	0 (0)	5 (100)	0 (0)	4 (80.0)	1 (20.0)
Rocket	Adverse	0 (0)	4 (16.7)	20 (83.3)	0 (0)	13 (54.2)	11 (45.8)	0 (0)	2 (8.3)	22 (91.7)	1 (4.2)	16 (66.7)	7 (29.2)

Table S1. *Cont.*

		ICAM1	rs1799969		IL12B	rs6887695		IL23R	rs11209026		IL23R	rs7517847	
		A/A	A/G	G/G	C/C	C/G	G/G	A/A	A/G	G/G	G/G	G/T	T/T
Watercress	No Difference	0 (0)	10 (13.9)	62 (86.1)	4 (5.5)	33 (45.2)	36 (49.3)	0 (0)	7 (9.5)	67 (90.5)	10 (13.7)	39 (53.4)	24 (32.9)
Watercress	Beneficial	2 (22.2)	1 (11.1)	6 (66.7)	1 (11.1)	4 (44.4)	4 (44.4)	0 (0)	0 (0)	9 (100)	2 (22.2)	5 (55.6)	2 (22.2)
Watercress	Adverse	0 (0)	1 (5.9)	16 (94.1)	2 (11.8)	10 (58.8)	5 (29.4)	0 (0)	1 (5.9)	16 (94.1)	1 (6.3)	4 (25.0)	11 (68.8)
Horseradish	No Difference	2 (2.3)	18 (20.9)	66 (76.7)	2 (2.3)	42 (47.7)	44 (50.0)	0 (0)	5 (5.6)	85 (94.4)	8 (9)	52 (58.4)	29 (32.6)
Horseradish	Beneficial	1 (50.0)	0 (0)	1 (50.0)	0 (0)	0 (0)	1 (100)	0 (0)	0 (0)	1 (100)	0 (0)	2 (100)	0 (0)
Horseradish	Adverse	0 (0)	8 (16.7)	40 (83.3)	7 (14.6)	20 (41.7)	21 (43.8)	0 (0)	5 (10.4)	43 (89.6)	3 (6.5)	22 (47.8)	21 (45.7)
Mustard powder	No Difference	1 (0.6)	29 (18.5)	127 (80.9)	4 (2.5)	84 (52.5)	72 (45)	0 (0)	11 (6.8)	151 (93.2)	17 (10.6)	83 (51.9)	60 (37.5)
Mustard powder	Beneficial	1 (33.3)	1 (33.3)	1 (33.3)	0 (0)	1 (33.3)	2 (66.7)	0 (0)	0 (0)	3 (100)	0 (0)	3 (100)	0 (0)
Mustard powder	Adverse	1 (1.9)	10 (19.2)	41 (78.9)	7 (13.2)	22 (41.5)	24 (45.3)	0 (0)	2 (3.8)	51 (96.2)	2 (3.9)	29 (55.8)	21 (40.4)
Mustard sauce	No Difference	2 (1.4)	26 (17.7)	119 (81)	4 (2.7)	73 (49)	72 (48.3)	0 (0)	11 (7.3)	140 (92.7)	13 (8.7)	82 (55.0)	54 (36.2)
Mustard sauce	Beneficial	1 (25.0)	0 (0)	3 (75.0)	0 (0)	2 (50.0)	2 (50.0)	0 (0)	0 (0)	4 (100)	0 (0)	2 (50.0)	2 (50.0)
Mustard sauce	Adverse	1 (1.6)	15 (23.4)	48 (75.0)	10 (15.4)	28 (43.1)	27 (41.5)	0 (0)	4 (6.2)	61 (93.9)	4 (6.4)	35 (55.6)	24 (38.1)
Wasabi	No Difference	1 (1.3)	14 (18)	63 (80.8)	2 (2.6)	38 (48.7)	38 (48.7)	0 (0)	5 (6.3)	75 (93.8)	6 (7.8)	39 (50.7)	32 (41.6)
Wasabi	Beneficial	1 (33.3)	0 (0)	2 (66.7)	0 (0)	2 (66.7)	1 (33.3)	0 (0)	0 (0)	3 (100)	0 (0)	3 (100)	0 (0)
Wasabi	Adverse	1 (1.7)	13 (22.4)	44 (75.9)	10 (16.7)	28 (46.7)	22 (36.7)	0 (0)	6 (10.0)	54 (90.0)	7 (12.1)	25 (43.1)	26 (44.8)

Table S1. *Cont.*

		**IL6**	**rs1800795**		**IRGM**	**rs4958847**		**ITLN1**	**rs2274910**		**JAK2**	**rs10758669**	
		C/C	C/G	G/G	A/A	A/G	G/G	C/C	C/T	T/T	A/A	A/C	C/C
Broccoli	No Difference	31 (17)	106 (58.2)	45 (24.7)	7 (3.9)	37 (20.8)	134 (75.3)	91 (46.4)	91 (46.4)	14 (7.1)	53 (29)	102 (55.7)	28 (15.3)
Broccoli	Beneficial	7 (33.3)	7 (33.3)	7 (33.3)	0 (0)	7 (33.3)	14 (66.7)	8 (33.3)	10 (41.7)	6 (25.0)	7 (31.8)	10 (45.5)	5 (22.7)
Broccoli	Adverse	7 (13.7)	27 (52.9)	17 (33.3)	1 (2)	11 (22)	38 (76)	25 (45.5)	25 (45.5)	5 (9.1)	19 (37.3)	22 (43.1)	10 (19.6)
Cabbage	No Difference	23 (18.7)	71 (57.7)	29 (23.6)	5 (4.2)	19 (15.8)	96 (80.0)	61 (44.9)	62 (45.6)	13 (9.6)	35 (28.0)	71 (56.8)	19 (15.2)
Cabbage	Beneficial	2 (28.6)	3 (42.9)	2 (28.6)	0 (0)	3 (42.9)	4 (57.1)	3 (42.9)	4 (57.1)	0 (0)	2 (28.6)	3 (42.9)	2 (28.6)
Cabbage	Adverse	18 (14.4)	69 (55.2)	38 (30.4)	2 (1.6)	32 (25.8)	90 (72.6)	56 (42.4)	66 (50.0)	10 (7.6)	42 (33.3)	62 (49.2)	22 (17.5)
Cauliflower	No Difference	32 (17.9)	101 (56.4)	46 (25.7)	7 (4.1)	35 (20.2)	131 (75.7)	83 (43.2)	93 (48.4)	16 (8.3)	44 (24.7)	103 (57.9)	31 (17.4)
Cauliflower	Beneficial	5 (27.8)	7 (38.9)	6 (33.3)	0 (0)	7 (38.9)	11 (61.1)	8 (40.0)	8 (40.0)	4 (20.0)	6 (31.6)	9 (47.4)	4 (21.1)
Cauliflower	Adverse	9 (14.8)	37 (60.7)	15 (24.6)	1 (1.6)	15 (24.2)	46 (74.2)	33 (49.3)	30 (44.8)	4 (6.0)	29 (46.0)	26 (41.3)	8 (12.7)
Chinese Greens	No Difference	17 (15.6)	66 (60.6)	26 (23.9)	6 (5.5)	15 (13.6)	89 (80.9)	51 (41.1)	60 (48.4)	13 (10.5)	33 (29.5)	60 (53.6)	19 (17)
Chinese Greens	Beneficial	2 (25.0)	2 (25.0)	4 (50.0)	0 (0)	5 (62.5)	3 (37.5)	3 (33.3)	4 (44.4)	2 (22.2)	3 (33.3)	6 (66.7)	0 (0)
Chinese Greens	Adverse	6 (18.2)	19 (57.6)	8 (24.2)	0 (0)	7 (21.2)	26 (78.8)	18 (52.9)	11 (32.4)	5 (14.7)	9 (28.1)	15 (46.9)	8 (25.0)
Rocket	No Difference	23 (15)	85 (55.6)	45 (29.4)	5 (3.3)	29 (19.2)	117 (77.5)	71 (41.5)	83 (48.5)	17 (9.9)	51 (32.7)	74 (47.4)	31 (19.9)
Rocket	Beneficial	4 (80.0)	1 (20.0)	0 (0)	0 (0)	1 (25.0)	3 (75.0)	3 (60.0)	1 (20.0)	1 (20.0)	2 (40.0)	2 (40.0)	1 (20.0)
Rocket	Adverse	3 (12.0)	16 (64.0)	6 (24.0)	2 (8.3)	4 (16.7)	18 (75.0)	12 (46.2)	11 (42.3)	3 (11.5)	5 (20.8)	15 (62.5)	4 (16.7)

Table S1. *Cont.*

		IL6	rs1800795		IRGM	rs4958847		ITLN1	rs2274910		JAK2	rs10758669	
		C/C	C/G	G/G	A/A	A/G	G/G	C/C	C/T	T/T	A/A	A/C	C/C
Watercress	No Difference	8 (11.1)	44 (61.1)	20 (27.8)	4 (5.7)	14 (20.0)	52 (74.3)	35 (42.7)	37 (45.1)	10 (12.2)	26 (36.1)	35 (48.6)	11 (15.3)
Watercress	Beneficial	2 (22.2)	5 (55.6)	2 (22.2)	0 (0)	2 (22.2)	7 (77.8)	3 (33.3)	5 (55.6)	1 (11.1)	1 (11.1)	5 (55.6)	3 (33.3)
Watercress	Adverse	2 (13.3)	7 (46.7)	6 (40.0)	0 (0)	4 (25.0)	12 (75.0)	8 (42.1)	7 (36.8)	4 (21.1)	4 (23.5)	8 (47.1)	5 (29.4)
Horseradish	No Difference	18 (20.7)	42 (48.3)	27 (31)	2 (2.3)	13 (14.9)	72 (82.8)	47 (48.0)	42 (42.9)	9 (9.2)	29 (33.0)	39 (44.3)	20 (22.7)
Horseradish	Beneficial	1 (50.0)	1 (50.0)	0 (0)	0 (0)	1 (50.0)	1 (50.0)	0 (0)	2 (100)	0 (0)	2 (100)	0 (0)	0 (0)
Horseradish	Adverse	8 (17)	29 (61.7)	10 (21.3)	0 (0)	10 (21.7)	36 (78.3)	21 (43.8)	21 (43.8)	6 (12.5)	13 (27.7)	28 (59.6)	6 (12.8)
Mustard powder	No Difference	26 (16.3)	92 (57.5)	42 (26.3)	7 (4.5)	27 (17.2)	123 (78.3)	81 (47.1)	77 (44.8)	14 (8.1)	54 (33.8)	78 (48.8)	28 (17.5)
Mustard powder	Beneficial	2 (66.7)	1 (33.3)	0 (0)	0 (0)	1 (33.3)	2 (66.7)	1 (33.3)	1 (33.3)	1 (33.3)	1 (33.3)	2 (66.7)	0 (0)
Mustard powder	Adverse	10 (18.5)	26 (48.2)	18 (33.3)	1 (1.9)	15 (27.8)	38 (70.4)	19 (32.2)	33 (55.9)	7 (11.9)	15 (27.3)	29 (52.7)	11 (20.0)
Mustard sauce	No Difference	24 (16.1)	81 (54.4)	44 (29.5)	7 (4.8)	29 (19.7)	111 (75.5)	76 (46.6)	76 (46.6)	11 (6.8)	46 (31.1)	76 (51.4)	26 (17.6)
Mustard sauce	Beneficial	3 (75.0)	1 (25.0)	0 (0)	0 (0)	1 (33.3)	2 (66.7)	2 (50.0)	2 (50.0)	0 (0)	3 (75.0)	1 (25.0)	0 (0)
Mustard sauce	Adverse	11 (17.2)	37 (57.8)	16 (25.0)	0 (0)	18 (27.7)	47 (72.3)	26 (37.7)	33 (47.8)	10 (14.5)	20 (29.9)	37 (55.2)	10 (14.9)
Wasabi	No Difference	13 (16.7)	44 (56.4)	21 (26.9)	3 (4.0)	11 (14.7)	61 (81.3)	45 (50.6)	37 (41.6)	7 (7.9)	23 (29.1)	39 (49.4)	17 (21.5)
Wasabi	Beneficial	2 (66.7)	1 (33.3)	0 (0)	0 (0)	1 (33.3)	2 (66.7)	2 (66.7)	1 (33.3)	0 (0)	2 (66.7)	0 (0)	1 (33.3)
Wasabi	Adverse	11 (19)	29 (50.0)	18 (31)	0 (0)	16 (27.1)	43 (72.9)	23 (36.5)	31 (49.2)	9 (14.3)	12 (20.7)	34 (58.6)	12 (20.7)

Table S1. *Cont.*

		JAM3	rs11604455		LAMP1	rs12871648		MAP1LC3B	rs2288483		MAP1LC3B	rs7204722	
		C/C	C/T	T/T	A/A	A/C	C/C	C/C	C/G	G/G	C/C	C/T	T/T
Broccoli	No Difference	146 (85.9)	24 (14.1)	0 (0)	69 (40.6)	81 (47.7)	20 (11.8)	3 (1.7)	37 (21.5)	132 (76.7)	4 (2.2)	52 (28.1)	129 (69.7)
Broccoli	Beneficial	19 (90.5)	2 (9.5)	0 (0)	5 (25.0)	12 (60.0)	3 (15)	1 (5)	10 (50.0)	9 (45)	1 (4.6)	11 (50.0)	10 (45.5)
Broccoli	Adverse	39 (78)	11 (22)	0 (0)	25 (49)	21 (41.2)	5 (9.8)	1 (2)	9 (17.7)	41 (80.4)	1 (1.9)	14 (26.9)	37 (71.2)
Cabbage	No Difference	96 (84.2)	18 (15.8)	0 (0)	47 (40.9)	56 (48.7)	12 (10.4)	2 (1.7)	28 (23.9)	87 (74.4)	4 (3.2)	39 (31)	83 (65.9)
Cabbage	Beneficial	7 (100)	0 (0)	0 (0)	2 (28.6)	3 (42.9)	2 (28.6)	0 (0)	5 (71.4)	2 (28.6)	0 (0)	4 (66.7)	2 (33.3)
Cabbage	Adverse	103 (83.7)	20 (16.3)	0 (0)	48 (39.3)	59 (48.4)	15 (12.3)	3 (2.5)	24 (19.7)	95 (77.9)	2 (1.6)	34 (26.6)	92 (71.9)
Cauliflower	No Difference	141 (85.5)	24 (14.6)	0 (0)	65 (38.9)	80 (47.9)	22 (13.2)	4 (2.4)	35 (20.7)	130 (76.9)	6 (3.3)	50 (27.6)	125 (69.1)
Cauliflower	Beneficial	16 (88.9)	2 (11.1)	0 (0)	4 (23.5)	10 (58.8)	3 (17.7)	0 (0)	9 (52.9)	8 (47.1)	0 (0)	9 (47.4)	10 (52.6)
Cauliflower	Adverse	48 (80.0)	12 (20.0)	0 (0)	31 (50.0)	27 (43.6)	4 (6.5)	0 (0)	12 (19.4)	50 (80.7)	0 (0)	18 (28.1)	46 (71.9)
Chinese Greens	No Difference	89 (84.8)	16 (15.2)	0 (0)	44 (41.5)	48 (45.3)	14 (13.2)	2 (1.9)	21 (19.6)	84 (78.5)	2 (1.7)	34 (29.6)	79 (68.7)
Chinese Greens	Beneficial	7 (77.8)	2 (22.2)	0 (0)	3 (33.3)	6 (66.7)	0 (0)	0 (0)	5 (55.6)	4 (44.4)	0 (0)	5 (62.5)	3 (37.5)
Chinese Greens	Adverse	27 (87.1)	4 (12.9)	0 (0)	13 (44.8)	15 (51.7)	1 (3.5)	1 (3.5)	11 (37.9)	17 (58.6)	1 (3)	12 (36.4)	20 (60.6)
Rocket	No Difference	130 (88.4)	17 (11.6)	0 (0)	61 (41.5)	66 (44.9)	20 (13.6)	3 (2)	35 (23.7)	110 (74.3)	3 (1.9)	50 (31.9)	104 (66.2)
Rocket	Beneficial	5 (100)	0 (0)	0 (0)	1 (20.0)	4 (80.0)	0 (0)	0 (0)	2 (40.0)	3 (60.0)	0 (0)	2 (40.0)	3 (60.0)
Rocket	Adverse	17 (70.8)	7 (29.2)	0 (0)	10 (41.7)	12 (50.0)	2 (8.3)	2 (8.3)	5 (20.8)	17 (70.8)	2 (8.0)	4 (16.0)	19 (76)
Watercress	No Difference	61 (88.4)	8 (11.6)	0 (0)	32 (46.4)	27 (39.1)	10 (14.5)	2 (2.9)	22 (31.4)	46 (65.7)	2 (2.7)	27 (36)	46 (61.3)

Table S1. *Cont.*

		**JAM3**	**rs11604455**		**LAMP1**	**rs12871648**		**MAP1LC3B**	**rs2288483**		**MAP1LC3B**	**rs7204722**	
		C/C	C/T	T/T	A/A	A/C	C/C	C/C	C/G	G/G	C/C	C/T	T/T
Watercress	Beneficial	8 (88.9)	1 (11.1)	0 (0)	4 (50.0)	3 (37.5)	1 (12.5)	0 (0)	4 (50.0)	4 (50.0)	0 (0)	3 (37.5)	5 (62.5)
Watercress	Adverse	15 (93.8)	1 (6.3)	0 (0)	5 (33.3)	8 (53.3)	2 (13.3)	0 (0)	3 (20.0)	12 (80.0)	0 (0)	5 (29.4)	12 (70.6)
Horseradish	No Difference	71 (87.7)	10 (12.4)	0 (0)	43 (50.6)	31 (36.5)	11 (12.9)	2 (2.3)	20 (23.3)	64 (74.4)	2 (2.2)	30 (33.3)	58 (64.4)
Horseradish	Beneficial	2 (100)	0 (0)	0 (0)	0 (0)	2 (100)	0 (0)	0 (0)	2 (100)	0 (0)	0 (0)	2 (100)	0 (0)
Horseradish	Adverse	38 (82.6)	8 (17.4)	0 (0)	15 (34.1)	24 (54.6)	5 (11.4)	1 (2.3)	13 (29.6)	30 (68.2)	1 (2.1)	13 (27.1)	34 (70.8)
Mustard powder	No Difference	127 (85.2)	22 (14.8)	0 (0)	67 (43.5)	67 (43.5)	20 (13)	3 (1.9)	35 (22.4)	118 (75.6)	4 (2.5)	52 (31.9)	107 (65.6)
Mustard powder	Beneficial	3 (100)	0 (0)	0 (0)	0 (0)	3 (100)	0 (0)	0 (0)	2 (66.7)	1 (33.3)	0 (0)	2 (66.7)	1 (33.3)
Mustard powder	Adverse	45 (86.5)	7 (13.5)	0 (0)	16 (31.4)	30 (58.8)	5 (9.8)	2 (3.9)	9 (17.7)	40 (78.4)	2 (3.6)	12 (21.4)	42 (75.0)
Mustard sauce	No Difference	118 (84.3)	22 (15.7)	0 (0)	65 (44.8)	61 (42.1)	19 (13.1)	2 (1.4)	34 (23.1)	111 (75.5)	3 (2)	48 (31.6)	101 (66.5)
Mustard sauce	Beneficial	4 (100)	0 (0)	0 (0)	2 (50.0)	2 (50.0)	0 (0)	0 (0)	1 (25.0)	3 (75.0)	0 (0)	1 (25.0)	3 (75.0)
Mustard sauce	Adverse	57 (90.5)	6 (9.5)	0 (0)	20 (32.8)	34 (55.7)	7 (11.5)	2 (3.3)	14 (23.0)	45 (73.8)	2 (3)	18 (27.3)	46 (69.7)
Wasabi	No Difference	65 (86.7)	10 (13.3)	0 (0)	33 (43.4)	32 (42.1)	11 (14.5)	0 (0)	20 (26)	57 (74)	1 (1.2)	27 (33.3)	53 (65.4)
Wasabi	Beneficial	3 (100)	0 (0)	0 (0)	1 (50.0)	1 (50.0)	0 (0)	0 (0)	1 (50.0)	1 (50.0)	0 (0)	1 (33.3)	2 (66.7)
Wasabi	Adverse	51 (89.5)	6 (10.5)	0 (0)	25 (44.6)	26 (46.4)	5 (8.9)	3 (5.4)	16 (28.6)	37 (66.1)	3 (5.1)	17 (28.8)	39 (66.1)

Table S1. *Cont.*

		**MAP1LC3B**	**rs8044820**		**MAP1LC3B**	**rs8051218**		**MIF**	**rs755622**		**NKX2-3**	**rs10883365**	
		A/A	A/G	G/G	C/C	C/T	T/T	C/C	C/G	G/G	A/A	A/G	G/G
Broccoli	No Difference	163 (86.7)	24 (12.8)	1 (0.5)	0 (0)	14 (7.7)	168 (92.3)	6 (4.6)	52 (40.0)	72 (55.4)	33 (18.1)	94 (51.7)	55 (30.2)
Broccoli	Beneficial	19 (86.4)	3 (13.6)	0 (0)	0 (0)	2 (9.1)	20 (90.9)	2 (14.3)	7 (50.0)	5 (35.7)	8 (36.4)	10 (45.5)	4 (18.2)
Broccoli	Adverse	44 (84.6)	8 (15.4)	0 (0)	0 (0)	3 (5.8)	49 (94.2)	1 (2.8)	11 (30.6)	24 (66.7)	9 (18)	30 (60.0)	11 (22)
Cabbage	No Difference	117 (91.4)	10 (7.8)	1 (0.8)	0 (0)	14 (11.3)	110 (88.7)	3 (3.4)	39 (44.3)	46 (52.3)	17 (13.9)	65 (53.3)	40 (32.8)
Cabbage	Beneficial	4 (57.1)	3 (42.9)	0 (0)	0 (0)	1 (14.3)	6 (85.7)	0 (0)	2 (66.7)	1 (33.3)	4 (57.1)	2 (28.6)	1 (14.3)
Cabbage	Adverse	109 (84.5)	20 (15.5)	0 (0)	0 (0)	6 (4.7)	121 (95.3)	5 (5.7)	31 (35.2)	52 (59.1)	30 (23.6)	66 (52.0)	31 (24.4)
Cauliflower	No Difference	160 (87)	23 (12.5)	1 (0.5)	0 (0)	15 (8.5)	162 (91.5)	6 (4.8)	51 (40.5)	69 (54.8)	30 (17)	97 (54.8)	50 (28.3)
Cauliflower	Beneficial	16 (84.2)	3 (15.8)	0 (0)	0 (0)	1 (5.3)	18 (94.7)	1 (8.3)	7 (58.3)	4 (33.3)	8 (42.1)	8 (42.1)	3 (15.8)
Cauliflower	Adverse	53 (84.1)	10 (15.9)	0 (0)	0 (0)	3 (4.7)	61 (95.3)	2 (4.6)	11 (25.0)	31 (70.5)	13 (21)	36 (58.1)	13 (21)
Chinese Greens	No Difference	98 (86)	15 (13.2)	1 (0.9)	0 (0)	10 (9)	101 (91)	3 (4.0)	32 (42.1)	41 (54)	21 (18.8)	59 (52.7)	32 (28.6)
Chinese Greens	Beneficial	6 (66.7)	3 (33.3)	0 (0)	0 (0)	2 (22.2)	7 (77.8)	1 (20.0)	2 (40.0)	2 (40.0)	3 (37.5)	5 (62.5)	0 (0)
Chinese Greens	Adverse	29 (85.3)	5 (14.7)	0 (0)	0 (0)	2 (6.1)	31 (93.9)	0 (0)	8 (29.6)	19 (70.4)	4 (12.1)	19 (57.6)	10 (30.3)
Rocket	No Difference	138 (86.3)	21 (13.1)	1 (0.6)	0 (0)	14 (8.9)	143 (91.1)	4 (3.7)	43 (39.8)	61 (56.5)	32 (20.8)	85 (55.2)	37 (24.0)
Rocket	Beneficial	4 (80.0)	1 (20.0)	0 (0)	0 (0)	0 (0)	5 (100)	1 (33.3)	1 (33.3)	1 (33.3)	3 (60.0)	2 (40.0)	0 (0)
Rocket	Adverse	21 (84)	4 (16.0)	0 (0)	0 (0)	0 (0)	24 (100)	2 (13.3)	4 (26.7)	9 (60.0)	3 (12.5)	13 (54.2)	8 (33.3)

Table S1. *Cont.*

		MAP1LC3B	rs8044820		MAP1LC3B	rs8051218		MIF	rs755622		NKX2-3	rs10883365	
		A/A	A/G	G/G	C/C	C/T	T/T	C/C	C/G	G/G	A/A	A/G	G/G
Watercress	No Difference	69 (90.8)	6 (7.9)	1 (1.3)	0 (0)	2 (2.8)	69 (97.2)	2 (3.9)	19 (37.3)	30 (58.8)	14 (19.2)	37 (50.7)	22 (30.1)
Watercress	Beneficial	7 (77.8)	2 (22.2)	0 (0)	0 (0)	1 (11.1)	8 (88.9)	0 (0)	3 (50.0)	3 (50.0)	3 (33.3)	5 (55.6)	1 (11.1)
Watercress	Adverse	14 (82.4)	3 (17.7)	0 (0)	0 (0)	2 (11.8)	15 (88.2)	0 (0)	3 (27.3)	8 (72.7)	3 (17.7)	9 (52.9)	5 (29.4)
Horseradish	No Difference	82 (89.1)	10 (10.9)	0 (0)	0 (0)	9 (10.2)	79 (89.8)	3 (5.4)	24 (42.9)	29 (51.8)	21 (23.9)	41 (46.6)	26 (29.6)
Horseradish	Beneficial	1 (50.0)	1 (50.0)	0 (0)	0 (0)	0 (0)	2 (100)	0 (0)	1 (100)	0 (0)	1 (100)	0 (0)	0 (0)
Horseradish	Adverse	44 (91.7)	4 (8.3)	0 (0)	0 (0)	1 (2.1)	47 (97.9)	3 (9.1)	10 (30.3)	20 (60.6)	6 (12.5)	27 (56.3)	15 (31.3)
Mustard powder	No Difference	139 (84.8)	24 (14.6)	1 (0.6)	0 (0)	13 (8.2)	146 (91.8)	7 (6.4)	45 (40.9)	58 (52.7)	34 (21.1)	79 (49.1)	48 (29.8)
Mustard powder	Beneficial	2 (66.7)	1 (33.3)	0 (0)	0 (0)	0 (0)	3 (100)	0 (0)	0 (0)	1 (100)	2 (66.7)	1 (33.3)	0 (0)
Mustard powder	Adverse	50 (87.7)	7 (12.3)	0 (0)	0 (0)	3 (5.3)	54 (94.7)	2 (5.1)	12 (30.8)	25 (64.1)	8 (15.1)	33 (62.3)	12 (22.6)
Mustard sauce	No Difference	129 (84.3)	23 (15)	1 (0.7)	0 (0)	14 (9.4)	135 (90.6)	5 (5.1)	46 (46.5)	48 (48.5)	31 (20.7)	69 (46.0)	50 (33.3)
Mustard sauce	Beneficial	4 (100)	0 (0)	0 (0)	0 (0)	0 (0)	4 (100)	1 (50.0)	0 (0)	1 (50.0)	3 (75.0)	0 (0)	1 (25.0)
Mustard sauce	Adverse	61 (91)	6 (9)	0 (0)	0 (0)	4 (6.1)	62 (93.9)	2 (4.1)	16 (32.7)	31 (63.3)	11 (16.9)	39 (60.0)	15 (23.1)
Wasabi	No Difference	69 (85.2)	12 (14.8)	0 (0)	0 (0)	6 (7.8)	71 (92.2)	4 (8.2)	19 (38.8)	26 (53.1)	22 (27.9)	36 (45.6)	21 (26.6)
Wasabi	Beneficial	3 (100)	0 (0)	0 (0)	0 (0)	0 (0)	3 (100)	0 (0)	0 (0)	1 (100)	1 (33.3)	0 (0)	2 (66.7)
Wasabi	Adverse	53 (86.9)	8 (13.1)	0 (0)	0 (0)	3 (5.1)	56 (94.9)	1 (2.3)	17 (38.6)	26 (59.1)	12 (20.0)	30 (50.0)	18 (30.0)

Table S1. *Cont.*

		**NOD2**	**rs2066844**		**OCTN1**	**rs1050152**		**OCTN2**	**rs2631367**		**P4HA2**	**rs4361509**	
		C/C	C/T	T/T	C/C	C/T	T/T	C/C	C/G	G/G	A/A	A/G	G/G
Broccoli	No Difference	152 (86.9)	23 (13.1)	0 (0)	49 (26.9)	106 (58.2)	27 (14.8)	37 (20.0)	107 (57.8)	41 (22.2)	30 (16.2)	106 (57.3)	49 (26.5)
Broccoli	Beneficial	16 (76.2)	5 (23.8)	0 (0)	9 (40.9)	8 (36.4)	5 (22.7)	5 (22.7)	8 (36.4)	9 (40.9)	7 (33.3)	6 (28.6)	8 (38.1)
Broccoli	Adverse	47 (94)	3 (6.0)	0 (0)	8 (16.3)	21 (42.9)	20 (40.8)	21 (41.2)	21 (41.2)	9 (17.7)	14 (27.5)	27 (52.9)	10 (19.6)
Cabbage	No Difference	104 (87.4)	15 (12.6)	0 (0)	35 (29.2)	65 (54.2)	20 (16.7)	29 (23.0)	67 (53.2)	30 (23.8)	25 (20.0)	62 (49.6)	38 (30.4)
Cabbage	Beneficial	5 (83.3)	1 (16.7)	0 (0)	1 (14.3)	3 (42.9)	3 (42.9)	3 (42.9)	3 (42.9)	1 (14.3)	2 (33.3)	2 (33.3)	2 (33.3)
Cabbage	Adverse	109 (87.9)	15 (12.1)	0 (0)	31 (24.6)	67 (53.2)	28 (22.2)	32 (25.2)	65 (51.2)	30 (23.6)	26 (20.5)	71 (55.9)	30 (23.6)
Cauliflower	No Difference	146 (85.9)	24 (14.1)	0 (0)	52 (29.4)	99 (55.9)	26 (14.7)	35 (19.4)	99 (55.0)	46 (25.6)	30 (16.7)	100 (55.6)	50 (27.8)
Cauliflower	Beneficial	14 (77.8)	4 (22.2)	0 (0)	6 (31.6)	8 (42.1)	5 (26.3)	5 (26.3)	8 (42.1)	6 (31.6)	7 (38.9)	5 (27.8)	6 (33.3)
Cauliflower	Adverse	59 (95.2)	3 (4.8)	0 (0)	11 (18)	30 (49.2)	20 (32.8)	23 (36.5)	30 (47.6)	10 (15.9)	14 (22.6)	35 (56.5)	13 (21)
Chinese Greens	No Difference	92 (85.2)	15 (13.9)	1 (0.9)	31 (28.4)	61 (56.0)	17 (15.6)	21 (18.6)	63 (55.8)	29 (25.7)	20 (17.9)	56 (50.0)	36 (32.1)
Chinese Greens	Beneficial	8 (88.9)	1 (11.1)	0 (0)	1 (11.1)	6 (66.7)	2 (22.2)	2 (22.2)	6 (66.7)	1 (11.1)	2 (25.0)	6 (75.0)	0 (0)
Chinese Greens	Adverse	29 (90.6)	3 (9.4)	0 (0)	9 (27.3)	15 (45.5)	9 (27.3)	9 (28.1)	16 (50.0)	7 (21.9)	8 (24.2)	19 (57.6)	6 (18.2)
Rocket	No Difference	133 (88.1)	18 (11.9)	0 (0)	40 (26.3)	87 (57.2)	25 (16.5)	31 (19.8)	91 (58.0)	35 (22.3)	28 (17.8)	82 (52.2)	47 (29.9)
Rocket	Beneficial	3 (60.0)	2 (40.0)	0 (0)	1 (20.0)	2 (40.0)	2 (40.0)	2 (40.0)	2 (40.0)	1 (20.0)	2 (40.0)	3 (60.0)	0 (0)
Rocket	Adverse	20 (87)	3 (13)	0 (0)	6 (25.0)	8 (33.3)	10 (41.7)	10 (41.7)	8 (33.3)	6 (25.0)	9 (37.5)	10 (41.7)	5 (20.8)
Watercress	No Difference	58 (81.7)	13 (18.3)	0 (0)	18 (25.4)	39 (54.9)	14 (19.7)	16 (21.9)	41 (56.2)	16 (21.9)	16 (21.9)	37 (50.7)	20 (27.4)

Table S1. *Cont.*

		NOD2	rs2066844		OCTN1	rs1050152		OCTN2	rs2631367		P4HA2	rs4361509	
		C/C	C/T	T/T	C/C	C/T	T/T	C/C	C/G	G/G	A/A	A/G	G/G
Watercress	Beneficial	6 (66.7)	3 (33.3)	0 (0)	1 (11.1)	6 (66.7)	2 (22.2)	2 (22.2)	6 (66.7)	1 (11.1)	3 (37.5)	2 (25.0)	3 (37.5)
Watercress	Adverse	16 (94.1)	1 (5.9)	0 (0)	4 (23.5)	9 (52.9)	4 (23.5)	4 (23.5)	9 (52.9)	4 (23.5)	2 (12.5)	10 (62.5)	4 (25.0)
Horseradish	No Difference	75 (85.2)	13 (14.8)	0 (0)	22 (25.6)	48 (55.8)	16 (18.6)	18 (20.2)	51 (57.3)	20 (22.5)	18 (20.2)	46 (51.7)	25 (28.1)
Horseradish	Beneficial	2 (100)	0 (0)	0 (0)	0 (0)	1 (50.0)	1 (50.0)	1 (50.0)	1 (50.0)	0 (0)	1 (50.0)	1 (50.0)	0 (0)
Horseradish	Adverse	40 (85.1)	6 (12.8)	1 (2.1)	9 (18.8)	27 (56.3)	12 (25.0)	12 (25.5)	27 (57.5)	8 (17)	9 (20.0)	26 (57.8)	10 (22.2)
Mustard powder	No Difference	139 (89.7)	16 (10.3)	0 (0)	44 (27.9)	92 (58.2)	22 (13.9)	29 (18)	89 (55.3)	43 (26.7)	26 (16.3)	84 (52.5)	50 (31.3)
Mustard powder	Beneficial	1 (33.3)	2 (66.7)	0 (0)	0 (0)	2 (66.7)	1 (33.3)	1 (33.3)	2 (66.7)	0 (0)	1 (33.3)	2 (66.7)	0 (0)
Mustard powder	Adverse	41 (77.4)	12 (22.6)	0 (0)	11 (20.8)	28 (52.8)	14 (26.4)	16 (28.6)	32 (57.1)	8 (14.3)	13 (23.2)	30 (53.6)	13 (23.2)
Mustard sauce	No Difference	125 (86.2)	20 (13.8)	0 (0)	35 (23.8)	84 (57.1)	28 (19.1)	36 (24.0)	81 (54)	33 (22)	30 (20.1)	77 (51.7)	42 (28.2)
Mustard sauce	Beneficial	4 (100)	0 (0)	0 (0)	1 (33.3)	1 (33.3)	1 (33.3)	1 (25.0)	1 (25.0)	2 (50.0)	2 (50.0)	1 (25.0)	1 (25.0)
Mustard sauce	Adverse	53 (80.3)	12 (18.2)	1 (1.5)	12 (18.2)	35 (53)	19 (28.8)	19 (28.4)	39 (58.2)	9 (13.4)	16 (25.0)	35 (54.7)	13 (20.3)
Wasabi	No Difference	66 (84.6)	12 (15.4)	0 (0)	19 (24.4)	46 (59)	13 (16.7)	14 (17.7)	48 (60.8)	17 (21.5)	14 (17.7)	40 (50.6)	25 (31.7)
Wasabi	Beneficial	2 (66.7)	1 (33.3)	0 (0)	1 (50.0)	0 (0)	1 (50.0)	1 (33.3)	0 (0)	2 (66.7)	1 (33.3)	0 (0)	2 (66.7)
Wasabi	Adverse	51 (87.9)	6 (10.3)	1 (1.7)	10 (17)	32 (54.2)	17 (28.8)	17 (28.8)	33 (55.9)	9 (15.3)	17 (29.3)	32 (55.2)	9 (15.5)

Table S1. *Cont.*

		PTGER4	rs1373692		PTGER4	rs4613763		PTPN2	rs2542151		SELS	rs4965814	
		A/A	A/C	C/C	C/C	C/T	T/T	G/G	G/T	T/T	C/C	C/T	T/T
Broccoli	No Difference	18 (10.3)	83 (47.7)	73 (42)	3 (1.7)	46 (25.3)	133 (73.1)	5 (7.9)	17 (27)	41 (65.1)	1 (0.5)	50 (27)	134 (72.4)
Broccoli	Beneficial	4 (19.1)	11 (52.4)	6 (28.6)	0 (0)	6 (28.6)	15 (71.4)	2 (33.3)	4 (66.7)	0 (0)	0 (0)	9 (40.9)	13 (59.1)
Broccoli	Adverse	2 (4.2)	22 (45.8)	24 (50.0)	2 (4.0)	15 (30.0)	33 (66)	0 (0)	5 (41.7)	7 (58.3)	3 (5.8)	18 (34.6)	31 (59.6)
Cabbage	No Difference	15 (12.6)	56 (47.1)	48 (40.3)	2 (1.6)	30 (24.2)	92 (74.2)	5 (11.4)	12 (27.3)	27 (61.4)	1 (0.8)	31 (25.0)	92 (74.2)
Cabbage	Beneficial	0 (0)	5 (71.4)	2 (28.6)	0 (0)	1 (14.3)	6 (85.7)	0 (0)	2 (100)	0 (0)	0 (0)	2 (28.6)	5 (71.4)
Cabbage	Adverse	11 (9.2)	53 (44.2)	56 (46.7)	5 (4.0)	39 (31.5)	80 (64.5)	2 (5.9)	13 (38.2)	19 (55.9)	3 (2.3)	45 (35.2)	80 (62.5)
Cauliflower	No Difference	18 (10.7)	82 (48.5)	69 (40.8)	5 (2.8)	45 (25.4)	127 (71.8)	4 (7.3)	17 (30.9)	34 (61.8)	3 (1.7)	48 (26.5)	130 (71.8)
Cauliflower	Beneficial	3 (16.7)	10 (55.6)	5 (27.8)	0 (0)	5 (27.8)	13 (72.2)	1 (25.0)	3 (75.0)	0 (0)	0 (0)	9 (47.4)	10 (52.6)
Cauliflower	Adverse	4 (6.6)	26 (42.6)	31 (50.8)	0 (0)	18 (29)	44 (71)	2 (8.7)	6 (26.1)	15 (65.2)	1 (1.6)	19 (30.7)	42 (67.7)
Chinese Greens	No Difference	9 (8.5)	49 (46.2)	48 (45.3)	2 (1.8)	30 (26.6)	81 (71.7)	4 (14.3)	8 (28.6)	16 (57.1)	2 (1.8)	36 (32.4)	73 (65.8)
Chinese Greens	Beneficial	2 (22.2)	6 (66.7)	1 (11.1)	0 (0)	3 (33.3)	6 (66.7)	1 (25.0)	1 (25.0)	2 (50.0)	1 (11.1)	4 (44.4)	4 (44.4)
Chinese Greens	Adverse	3 (9.4)	11 (34.4)	18 (56.3)	1 (3)	14 (42.4)	18 (54.6)	0 (0)	5 (50.0)	5 (50.0)	1 (3)	14 (42.4)	18 (54.6)
Rocket	No Difference	13 (8.9)	69 (47.3)	64 (43.8)	4 (2.6)	41 (26.8)	108 (70.6)	5 (10.2)	18 (36.7)	26 (53.1)	3 (2)	45 (29.2)	106 (68.8)
Rocket	Beneficial	1 (20.0)	3 (60.0)	1 (20.0)	0 (0)	0 (0)	5 (100)	0 (0)	1 (50.0)	1 (50.0)	0 (0)	2 (40.0)	3 (60.0)
Rocket	Adverse	3 (12.5)	10 (41.7)	11 (45.8)	0 (0)	8 (33.3)	16 (66.7)	1 (16.7)	3 (50.0)	2 (33.3)	1 (4.0)	11 (44.0)	13 (52.0)
Watercress	No Difference	7 (9.7)	34 (47.2)	31 (43.1)	3 (4.1)	18 (24.3)	53 (71.6)	3 (11.1)	12 (44.4)	12 (44.4)	0 (0)	30 (41.1)	43 (58.9)

Table S1. *Cont.*

		PTGER4	rs1373692		PTGER4	rs4613763		PTPN2	rs2542151		SELS	rs4965814	
		A/A	A/C	C/C	C/C	C/T	T/T	G/G	G/T	T/T	C/C	C/T	T/T
Watercress	Beneficial	1 (11.1)	5 (55.6)	3 (33.3)	0 (0)	2 (22.2)	7 (77.8)	1 (100)	0 (0)	0 (0)	0 (0)	4 (44.4)	5 (55.6)
Watercress	Adverse	3 (20.0)	2 (13.3)	10 (66.7)	0 (0)	5 (31.3)	11 (68.8)	0 (0)	1 (50.0)	1 (50.0)	2 (11.8)	7 (41.2)	8 (47.1)
Horseradish	No Difference	6 (7.0)	39 (45.4)	41 (47.7)	1 (1.1)	23 (25.6)	66 (73.3)	2 (8.0)	10 (40.0)	13 (52.0)	3 (3.4)	24 (27)	62 (69.7)
Horseradish	Beneficial	0 (0)	2 (100)	0 (0)	0 (0)	1 (50.0)	1 (50.0)	0 (0)	0 (0)	1 (100)	0 (0)	0 (0)	2 (100)
Horseradish	Adverse	6 (13)	18 (39.1)	22 (47.8)	1 (2.1)	18 (38.3)	28 (59.6)	2 (15.4)	5 (38.5)	6 (46.2)	0 (0)	25 (52.1)	23 (47.9)
Mustard powder	No Difference	14 (9)	73 (47.1)	68 (43.9)	5 (3.1)	34 (21)	123 (75.9)	4 (8.3)	19 (39.6)	25 (52.1)	3 (1.9)	49 (30.4)	109 (67.7)
Mustard powder	Beneficial	0 (0)	2 (66.7)	1 (33.3)	0 (0)	0 (0)	3 (100)	0 (0)	0 (0)	1 (100)	0 (0)	1 (33.3)	2 (66.7)
Mustard powder	Adverse	8 (15.4)	25 (48.1)	19 (36.5)	1 (1.9)	22 (42.3)	29 (55.8)	2 (10.5)	6 (31.6)	11 (57.9)	0 (0)	17 (30.9)	38 (69.1)
Mustard sauce	No Difference	12 (8.3)	69 (47.6)	64 (44.1)	4 (2.7)	37 (24.5)	110 (72.9)	3 (7.5)	15 (37.5)	22 (55.0)	3 (2)	46 (30.7)	101 (67.3)
Mustard sauce	Beneficial	1 (25.0)	2 (50.0)	1 (25.0)	0 (0)	1 (25.0)	3 (75.0)	0 (0)	1 (50.0)	1 (50.0)	0 (0)	2 (50.0)	2 (50.0)
Mustard sauce	Adverse	9 (14.5)	25 (40.3)	28 (45.2)	0 (0)	21 (33.3)	42 (66.7)	2 (9.5)	6 (28.6)	13 (61.9)	0 (0)	25 (37.3)	42 (62.7)
Wasabi	No Difference	7 (9.2)	34 (44.7)	35 (46.1)	2 (2.5)	20 (25.3)	57 (72.2)	2 (9.1)	8 (36.4)	12 (54.6)	3 (3.8)	27 (33.8)	50 (62.5)
Wasabi	Beneficial	1 (33.3)	2 (66.7)	0 (0)	0 (0)	0 (0)	3 (100)	0 (0)	0 (0)	1 (100)	0 (0)	1 (33.3)	2 (66.7)
Wasabi	Adverse	5 (8.8)	25 (43.9)	27 (47.4)	1 (1.8)	21 (36.8)	35 (61.4)	2 (14.3)	5 (35.7)	7 (50.0)	0 (0)	24 (41.4)	34 (58.6)

Table S1. *Cont.*

		SELS rs7178239			xSEP15 rs5845			xSEP15 rs5859			SEPP1 rs3877899		
		C/C	C/G	G/G	A/A	A/G	G/G	C/C	C/T	T/T	A/A	A/G	G/G
Broccoli	No Difference	138 (74.6)	46 (24.9)	1 (0.5)	12 (6.7)	51 (28.5)	116 (64.8)	118 (66.3)	49 (27.5)	11 (6.2)	10 (5.6)	53 (29.6)	116 (64.8)
Broccoli	Beneficial	14 (63.6)	8 (36.4)	0 (0)	3 (13.6)	8 (36.4)	11 (50.0)	11 (52.4)	6 (28.6)	4 (19.1)	0 (0)	7 (35.0)	13 (65)
Broccoli	Adverse	32 (61.5)	17 (32.7)	3 (5.8)	3 (5.9)	22 (43.1)	26 (51)	26 (52.0)	21 (42)	3 (6.0)	3 (5.9)	12 (23.5)	36 (70.6)
Cabbage	No Difference	94 (75.8)	29 (23.4)	1 (0.8)	6 (5)	30 (24.8)	85 (70.3)	86 (71.7)	29 (24.2)	5 (4.2)	6 (4.8)	35 (28.0)	84 (67.2)
Cabbage	Beneficial	5 (71.4)	2 (28.6)	0 (0)	3 (42.9)	2 (28.6)	2 (28.6)	2 (28.6)	2 (28.6)	3 (42.9)	0 (0)	3 (50.0)	3 (50.0)
Cabbage	Adverse	83 (64.3)	42 (32.6)	4 (3.1)	11 (8.7)	49 (38.9)	66 (52.4)	67 (54.9)	43 (35.3)	12 (9.8)	5 (4.1)	38 (31.2)	79 (64.8)
Cauliflower	No Difference	134 (74)	44 (24.3)	3 (1.7)	15 (8.5)	53 (29.9)	109 (61.6)	111 (63.4)	50 (28.6)	14 (8.0)	11 (6.3)	51 (29.1)	113 (64.6)
Cauliflower	Beneficial	11 (57.9)	8 (42.1)	0 (0)	2 (10.5)	7 (36.8)	10 (52.6)	10 (55.6)	5 (27.8)	3 (16.7)	0 (0)	7 (41.2)	10 (58.8)
Cauliflower	Adverse	43 (68.3)	18 (28.6)	2 (3.2)	3 (5)	22 (36.7)	35 (58.3)	36 (59)	22 (36.1)	3 (4.9)	2 (3.2)	18 (29)	42 (67.7)
Chinese Greens	No Difference	74 (66.7)	35 (31.5)	2 (1.8)	10 (9.3)	32 (29.6)	66 (61.1)	69 (64.5)	29 (27.1)	9 (8.4)	8 (7.1)	35 (31.3)	69 (61.6)
Chinese Greens	Beneficial	4 (44.4)	4 (44.4)	1 (11.1)	1 (11.1)	3 (33.3)	5 (55.6)	5 (55.6)	2 (22.2)	2 (22.2)	0 (0)	1 (16.7)	5 (83.3)
Chinese Greens	Adverse	21 (63.6)	11 (33.3)	1 (3)	1 (3)	10 (30.3)	22 (66.7)	22 (68.8)	9 (28.1)	1 (3.1)	0 (0)	7 (20.6)	27 (79.4)
Rocket	No Difference	108 (69.7)	43 (27.7)	4 (2.6)	12 (8.0)	41 (27.2)	98 (64.9)	99 (66)	40 (26.7)	11 (7.3)	11 (7.1)	51 (32.9)	93 (60.0)
Rocket	Beneficial	3 (60.0)	2 (40.0)	0 (0)	1 (20.0)	2 (40.0)	2 (40.0)	2 (40.0)	2 (40.0)	1 (20.0)	0 (0)	1 (20.0)	4 (80.0)
Rocket	Adverse	15 (60.0)	9 (36)	1 (4.0)	1 (4.0)	12 (48.0)	12 (48.0)	11 (47.8)	11 (47.8)	1 (4.4)	0 (0)	6 (26.1)	17 (73.9)
Watercress	No Difference	46 (63)	27 (37)	0 (0)	3 (4.2)	16 (22.5)	52 (73.2)	54 (76.1)	14 (19.7)	3 (4.2)	3 (4.1)	16 (21.6)	55 (74.3)
Watercress	Beneficial	6 (66.7)	3 (33.3)	0 (0)	1 (11.1)	2 (22.2)	6 (66.7)	6 (66.7)	2 (22.2)	1 (11.1)	0 (0)	2 (28.6)	5 (71.4)

Table S1. *Cont.*

		SELS	rs7178239		xSEP15	rs5845		xSEP15	rs5859		SEPP1	rs3877899	
		C/C	C/G	G/G	A/A	A/G	G/G	C/C	C/T	T/T	A/A	A/G	G/G
Watercress	Adverse	8 (47.1)	7 (41.2)	2 (11.8)	0 (0)	10 (58.8)	7 (41.2)	7 (46.7)	7 (46.7)	1 (6.7)	0 (0)	8 (50.0)	8 (50.0)
Horseradish	No Difference	63 (70.8)	23 (25.8)	3 (3.4)	5 (5.9)	28 (32.9)	52 (61.2)	53 (62.4)	28 (32.9)	4 (4.7)	7 (8.0)	23 (26.1)	58 (65.9)
Horseradish	Beneficial	2 (100)	0 (0)	0 (0)	0 (0)	2 (100)	0 (0)	0 (0)	2 (100)	0 (0)	0 (0)	0 (0)	2 (100)
Horseradish	Adverse	26 (54.2)	22 (45.8)	0 (0)	2 (4.2)	20 (41.7)	26 (54.2)	26 (57.8)	16 (35.6)	3 (6.7)	0 (0)	13 (27.7)	34 (72.3)
Mustard powder	No Difference	112 (69.1)	46 (28.4)	4 (2.5)	16 (10.1)	38 (23.9)	105 (66)	104 (66.2)	38 (24.2)	15 (9.6)	12 (7.6)	48 (30.6)	97 (61.8)
Mustard powder	Beneficial	2 (66.7)	1 (33.3)	0 (0)	1 (33.3)	2 (66.7)	0 (0)	0 (0)	2 (66.7)	1 (33.3)	0 (0)	1 (33.3)	2 (66.7)
Mustard powder	Adverse	39 (70.9)	16 (29.1)	0 (0)	3 (5.6)	24 (44.4)	27 (50.0)	28 (51.9)	23 (42.6)	3 (5.6)	0 (0)	19 (35.2)	35 (64.8)
Mustard sauce	No Difference	104 (69.3)	43 (28.7)	3 (2)	10 (6.8)	38 (25.9)	99 (67.4)	100 (69)	36 (24.8)	9 (6.2)	9 (6.2)	41 (28.1)	96 (65.8)
Mustard sauce	Beneficial	2 (50.0)	2 (50.0)	0 (0)	0 (0)	1 (25.0)	3 (75.0)	3 (75.0)	1 (25.0)	0 (0)	0 (0)	1 (25.0)	3 (75.0)
Mustard sauce	Adverse	44 (65.7)	23 (34.3)	0 (0)	6 (9.1)	25 (37.9)	35 (53)	35 (55.6)	21 (33.3)	7 (11.1)	0 (0)	20 (31.3)	44 (68.8)
Wasabi	No Difference	51 (63.8)	26 (32.5)	3 (3.8)	7 (9.1)	23 (29.9)	47 (61)	49 (64.5)	21 (27.6)	6 (7.9)	8 (10.3)	25 (32.1)	45 (57.7)
Wasabi	Beneficial	3 (100)	0 (0)	0 (0)	0 (0)	2 (66.7)	1 (33.3)	1 (33.3)	2 (66.7)	0 (0)	0 (0)	1 (33.3)	2 (66.7)
Wasabi	Adverse	37 (62.7)	21 (35.6)	1 (1.7)	3 (5.1)	24 (40.7)	32 (54.2)	32 (58.2)	19 (34.6)	4 (7.3)	0 (0)	23 (38.3)	37 (61.7)

Table S1. *Cont.*

		SLC11A1	rs3731865		SLC11A2	rs224589		SLC11A2	rs427020		SLC6A6	rs41284011	
		C/C	C/G	G/G	G/G	G/T	T/T	C/C	C/T	T/T	C/C	C/T	T/T
Broccoli	No Difference	18 (10.3)	69 (39.7)	87 (50.0)	100 (55.3)	71 (39.2)	10 (5.5)	32 (20.1)	75 (47.2)	52 (32.7)	184 (99.5)	1 (0.5)	0 (0)
Broccoli	Beneficial	2 (9.1)	7 (31.8)	13 (59.1)	10 (47.6)	10 (47.6)	1 (4.8)	2 (11.8)	10 (58.8)	5 (29.4)	21 (95.5)	1 (4.6)	0 (0)
Broccoli	Adverse	6 (11.8)	17 (33.3)	28 (54.9)	24 (48.0)	19 (38.0)	7 (14.0)	9 (18.8)	25 (52.1)	14 (29.2)	52 (100)	0 (0)	0 (0)
Cabbage	No Difference	10 (8.3)	46 (38.3)	64 (53.3)	68 (54.8)	51 (41.1)	5 (4.0)	21 (19.1)	57 (51.8)	32 (29.1)	124 (99.2)	1 (0.8)	0 (0)
Cabbage	Beneficial	1 (20.0)	0 (0)	4 (80.0)	3 (42.9)	2 (28.6)	2 (28.6)	3 (60.0)	0 (0)	2 (40.0)	6 (85.7)	1 (14.3)	0 (0)
Cabbage	Adverse	14 (11.6)	41 (33.9)	66 (54.6)	64 (52.0)	48 (39)	11 (8.9)	22 (19.6)	57 (50.9)	33 (29.5)	129 (100)	0 (0)	0 (0)
Cauliflower	No Difference	20 (11.7)	67 (39.2)	84 (49.1)	95 (53.7)	73 (41.2)	9 (5.1)	30 (19)	75 (47.5)	53 (33.5)	180 (99.5)	1 (0.6)	0 (0)
Cauliflower	Beneficial	1 (5.3)	6 (31.6)	12 (63.2)	7 (38.9)	10 (55.6)	1 (5.6)	2 (14.3)	8 (57.1)	4 (28.6)	18 (94.7)	1 (5.3)	0 (0)
Cauliflower	Adverse	4 (6.5)	19 (30.7)	39 (62.9)	32 (52.5)	21 (34.4)	8 (13.1)	12 (21.1)	34 (59.7)	11 (19.3)	63 (100)	0 (0)	0 (0)
Chinese Greens	No Difference	9 (8.3)	42 (38.9)	57 (52.8)	58 (52.3)	46 (41.4)	7 (6.3)	20 (20.4)	51 (52.0)	27 (27.6)	111 (99.1)	1 (0.9)	0 (0)
Chinese Greens	Beneficial	1 (12.5)	1 (12.5)	6 (75.0)	2 (22.2)	6 (66.7)	1 (11.1)	3 (37.5)	4 (50.0)	1 (12.5)	9 (100)	0 (0)	0 (0)
Chinese Greens	Adverse	3 (9.7)	9 (29)	19 (61.3)	17 (51.5)	14 (42.4)	2 (6.1)	7 (25.0)	14 (50.0)	7 (25.0)	33 (100)	0 (0)	0 (0)
Rocket	No Difference	13 (8.7)	52 (34.9)	84 (56.4)	79 (51)	67 (43.2)	9 (5.8)	29 (21.6)	68 (50.8)	37 (27.6)	154 (98.7)	2 (1.3)	0 (0)
Rocket	Beneficial	1 (20.0)	0 (0)	4 (80.0)	4 (80.0)	0 (0)	1 (20.0)	1 (25.0)	2 (50.0)	1 (25.0)	5 (100)	0 (0)	0 (0)
Rocket	Adverse	3 (12.0)	8 (32)	14 (56.0)	17 (70.8)	7 (29.2)	0 (0)	0 (0)	11 (50.0)	11 (50.0)	25 (100)	0 (0)	0 (0)
Watercress	No Difference	6 (8.6)	21 (30.0)	43 (61.4)	34 (46.6)	36 (49.3)	3 (4.1)	14 (21.2)	36 (54.6)	16 (24.2)	72 (98.6)	1 (1.4)	0 (0)

Table S1. *Cont.*

		SLC11A1	rs3731865		SLC11A2	rs224589		SLC11A2	rs427020		SLC6A6	rs41284011	
		C/C	C/G	G/G	G/G	G/T	T/T	C/C	C/T	T/T	C/C	C/T	T/T
Watercress	Beneficial	1 (12.5)	2 (25.0)	5 (62.5)	4 (44.4)	5 (55.6)	0 (0)	1 (14.3)	4 (57.1)	2 (28.6)	8 (88.9)	1 (11.1)	0 (0)
Watercress	Adverse	1 (6.3)	6 (37.5)	9 (56.3)	4 (25.0)	8 (50.0)	4 (25.0)	6 (42.9)	6 (42.9)	2 (14.3)	17 (100)	0 (0)	0 (0)
Horseradish	No Difference	8 (9.3)	32 (37.2)	46 (53.5)	50 (56.2)	34 (38.2)	5 (5.6)	15 (20.0)	33 (44.0)	27 (36)	88 (98.9)	1 (1.1)	0 (0)
Horseradish	Beneficial	0 (0)	0 (0)	2 (100)	1 (50.0)	1 (50.0)	0 (0)	0 (0)	1 (100)	0 (0)	2 (100)	0 (0)	0 (0)
Horseradish	Adverse	5 (10.9)	13 (28.3)	28 (60.9)	22 (48.9)	19 (42.2)	4 (8.9)	9 (21.4)	21 (50.0)	12 (28.6)	48 (100)	0 (0)	0 (0)
Mustard powder	No Difference	13 (8.4)	63 (40.7)	79 (51)	88 (55.4)	63 (39.6)	8 (5)	26 (18.2)	71 (49.7)	46 (32.2)	161 (98.8)	2 (1.2)	0 (0)
Mustard powder	Beneficial	1 (33.3)	0 (0)	2 (66.7)	2 (66.7)	0 (0)	1 (33.3)	1 (50.0)	0 (0)	1 (50.0)	3 (100)	0 (0)	0 (0)
Mustard powder	Adverse	9 (16.7)	11 (20.4)	34 (63)	23 (43.4)	26 (49.1)	4 (7.6)	8 (17)	27 (57.5)	12 (25.5)	55 (100)	0 (0)	0 (0)
Mustard sauce	No Difference	13 (9.1)	60 (42)	70 (49)	79 (52.7)	60 (40.0)	11 (7.3)	23 (17.4)	66 (50.0)	43 (32.6)	149 (98.7)	2 (1.3)	0 (0)
Mustard sauce	Beneficial	0 (0)	1 (25.0)	3 (75.0)	4 (100)	0 (0)	0 (0)	0 (0)	2 (66.7)	1 (33.3)	4 (100)	0 (0)	0 (0)
Mustard sauce	Adverse	8 (12.3)	18 (27.7)	39 (60.0)	30 (47.6)	29 (46.0)	4 (6.4)	9 (15.5)	32 (55.2)	17 (29.3)	67 (100)	0 (0)	0 (0)
Wasabi	No Difference	8 (10.7)	21 (28.0)	46 (61.3)	39 (49.4)	37 (46.8)	3 (3.8)	9 (13.2)	35 (51.5)	24 (35.3)	78 (97.5)	2 (2.5)	0 (0)
Wasabi	Beneficial	0 (0)	0 (0)	3 (100)	2 (66.7)	1 (33.3)	0 (0)	0 (0)	1 (100)	0 (0)	3 (100)	0 (0)	0 (0)
Wasabi	Adverse	7 (12.3)	18 (31.6)	32 (56.1)	30 (51.7)	22 (37.9)	6 (10.3)	12 (21.8)	30 (54.6)	13 (23.6)	59 (100)	0 (0)	0 (0)

Table S1. *Cont.*

		**SLC6A6**	**rs4685154**		**STAT3**	**rs744166**		**TF**	**rs1799899**		**TFF3**	**rs225369**	
		C/C	C/G	G/G	A/A	A/G	G/G	A/A	A/G	G/G	C/C	C/G	G/G
Broccoli	No Difference	64 (35.0)	88 (48.1)	31 (16.9)	72 (40.2)	81 (45.3)	26 (14.5)	0 (0)	16 (8.5)	172 (91.5)	50 (27.3)	79 (43.2)	54 (29.5)
Broccoli	Beneficial	7 (31.8)	12 (54.6)	3 (13.6)	8 (36.4)	12 (54.6)	2 (9.1)	0 (0)	3 (13.6)	19 (86.4)	6 (27.3)	12 (54.6)	4 (18.2)
Broccoli	Adverse	18 (34.6)	27 (51.9)	7 (13.5)	21 (42)	21 (42)	8 (16.0)	1 (1.9)	7 (13.5)	44 (84.6)	11 (21.2)	24 (46.2)	17 (32.7)
Cabbage	No Difference	39 (31.2)	61 (48.8)	25 (20.0)	38 (30.9)	63 (51.2)	22 (17.9)	0 (0)	8 (6.4)	118 (93.7)	33 (26.4)	58 (46.4)	34 (27.2)
Cabbage	Beneficial	2 (28.6)	3 (42.9)	2 (28.6)	2 (28.6)	5 (71.4)	0 (0)	0 (0)	1 (14.3)	6 (85.7)	3 (42.9)	3 (42.9)	1 (14.3)
Cabbage	Adverse	46 (36.2)	61 (48.0)	20 (15.8)	61 (49.6)	47 (38.2)	15 (12.2)	0 (0)	14 (10.8)	116 (89.2)	33 (25.8)	55 (43)	40 (31.3)
Cauliflower	No Difference	64 (35.6)	87 (48.3)	29 (16.1)	69 (39.7)	80 (46.0)	25 (14.4)	0 (0)	16 (8.7)	167 (91.3)	54 (30.2)	77 (43)	48 (26.8)
Cauliflower	Beneficial	6 (31.6)	10 (52.6)	3 (15.8)	7 (36.8)	11 (57.9)	1 (5.3)	0 (0)	3 (15.8)	16 (84.2)	6 (31.6)	10 (52.6)	3 (15.8)
Cauliflower	Adverse	21 (33.3)	31 (49.2)	11 (17.5)	25 (40.3)	26 (41.9)	11 (17.7)	0 (0)	7 (10.9)	57 (89.1)	12 (19.1)	27 (42.9)	24 (38.1)
Chinese Greens	No Difference	45 (40.2)	50 (44.6)	17 (15.2)	39 (34.8)	61 (54.5)	12 (10.7)	1 (0.9)	9 (8.0)	103 (91.2)	34 (30.4)	44 (39.3)	34 (30.4)
Chinese Greens	Beneficial	2 (22.2)	5 (55.6)	2 (22.2)	2 (25.0)	4 (50.0)	2 (25.0)	0 (0)	2 (22.2)	7 (77.8)	2 (22.2)	5 (55.6)	2 (22.2)
Chinese Greens	Adverse	7 (21.2)	19 (57.6)	7 (21.2)	11 (33.3)	16 (48.5)	6 (18.2)	0 (0)	5 (14.7)	29 (85.3)	9 (27.3)	17 (51.5)	7 (21.2)
Rocket	No Difference	53 (34.4)	72 (46.8)	29 (18.8)	57 (37.5)	74 (48.7)	21 (13.8)	2 (1.3)	13 (8.2)	143 (90.5)	39 (25.2)	69 (44.5)	47 (30.3)
Rocket	Beneficial	2 (40.0)	3 (60.0)	0 (0)	2 (40.0)	3 (60.0)	0 (0)	0 (0)	1 (20.0)	4 (80.0)	2 (40.0)	2 (40.0)	1 (20.0)
Rocket	Adverse	7 (28.0)	12 (48.0)	6 (24.0)	9 (39.1)	11 (47.8)	3 (13)	0 (0)	2 (8.0)	23 (92)	10 (40.0)	4 (16.0)	11 (44.0)
Watercress	No Difference	29 (39.7)	33 (45.2)	11 (15.1)	24 (33.3)	39 (54.2)	9 (12.5)	0 (0)	8 (10.7)	67 (89.3)	22 (29.7)	31 (41.9)	21 (28.4)
Watercress	Beneficial	2 (22.2)	4 (44.4)	3 (33.3)	2 (22.2)	6 (66.7)	1 (11.1)	0 (0)	1 (11.1)	8 (88.9)	5 (55.6)	3 (33.3)	1 (11.1)

Table S1. *Cont.*

		**SLC6A6**	**rs4685154**		**STAT3**	**rs744166**		**TF**	**rs1799899**		**TFF3**	**rs225369**	
		C/C	C/G	G/G	A/A	A/G	G/G	A/A	A/G	G/G	C/C	C/G	G/G
Watercress	Adverse	5 (29.4)	8 (47.1)	4 (23.5)	7 (41.2)	7 (41.2)	3 (17.7)	0 (0)	1 (5.9)	16 (94.1)	4 (23.5)	11 (64.7)	2 (11.8)
Horseradish	No Difference	28 (31.8)	45 (51.1)	15 (17.1)	34 (38.2)	43 (48.3)	12 (13.5)	1 (1.1)	4 (4.4)	86 (94.5)	25 (28.1)	35 (39.3)	29 (32.6)
Horseradish	Beneficial	2 (100)	0 (0)	0 (0)	1 (100)	0 (0)	0 (0)	0 (0)	0 (0)	2 (100)	0 (0)	0 (0)	2 (100)
Horseradish	Adverse	17 (35.4)	24 (50.0)	7 (14.6)	19 (40.4)	23 (48.9)	5 (10.6)	0 (0)	6 (12.5)	42 (87.5)	12 (25.0)	21 (43.8)	15 (31.3)
Mustard powder	No Difference	55 (34)	75 (46.3)	32 (19.8)	61 (38.6)	78 (49.4)	19 (12.0)	1 (0.6)	14 (8.6)	147 (90.7)	43 (26.7)	64 (39.8)	54 (33.5)
Mustard powder	Beneficial	2 (66.7)	1 (33.3)	0 (0)	2 (66.7)	1 (33.3)	0 (0)	0 (0)	1 (33.3)	2 (66.7)	1 (33.3)	1 (33.3)	1 (33.3)
Mustard powder	Adverse	20 (37)	26 (48.2)	8 (14.8)	23 (43.4)	24 (45.3)	6 (11.3)	1 (1.8)	5 (8.8)	51 (89.5)	19 (34.6)	22 (40.0)	14 (25.5)
Mustard sauce	No Difference	54 (36)	70 (46.7)	26 (17.3)	57 (38.5)	70 (47.3)	21 (14.2)	1 (0.7)	14 (9.2)	137 (90.1)	42 (27.8)	63 (41.7)	46 (30.5)
Mustard sauce	Beneficial	1 (25.0)	3 (75.0)	0 (0)	1 (25.0)	2 (50.0)	1 (25.0)	0 (0)	0 (0)	4 (100)	0 (0)	3 (75.0)	1 (25.0)
Mustard sauce	Adverse	22 (33.3)	34 (51.5)	10 (15.2)	29 (45.3)	29 (45.3)	6 (9.4)	1 (1.5)	7 (10.5)	59 (88.1)	18 (27.3)	30 (45.5)	18 (27.3)
Wasabi	No Difference	27 (33.8)	38 (47.5)	15 (18.8)	25 (32.1)	42 (53.9)	11 (14.1)	1 (1.3)	7 (8.8)	72 (90.0)	18 (22.8)	35 (44.3)	26 (32.9)
Wasabi	Beneficial	2 (66.7)	1 (33.3)	0 (0)	1 (33.3)	1 (33.3)	1 (33.3)	0 (0)	0 (0)	3 (100)	1 (33.3)	1 (33.3)	1 (33.3)
Wasabi	Adverse	17 (29.3)	28 (48.3)	13 (22.4)	25 (42.4)	27 (45.8)	7 (11.9)	1 (1.6)	4 (6.6)	56 (91.8)	15 (25.4)	29 (49.2)	15 (25.4)

Table S1. *Cont.*

		**TLR9**	**rs5743836**		**TNFALPHA**	**rs1800629**		**TNFRSF1B**	**rs3397**		**TNFSF15**	**rs10982412**	
		C/C	C/T	T/T	A/A	A/G	G/G	C/C	C/T	T/T	A/A	A/G	G/G
Broccoli	No Difference	3 (1.8)	50 (29.2)	118 (69)	6 (3.3)	48 (26.4)	128 (70.3)	3 (42.9)	3 (42.9)	1 (14.3)	4 (2.6)	41 (26.6)	109 (70.8)
Broccoli	Beneficial	1 (4.8)	4 (19.1)	16 (76.2)	0 (0)	8 (36.4)	14 (63.6)	0 (0)	0 (0)	0 (0)	0 (0)	2 (9.5)	19 (90.5)
Broccoli	Adverse	3 (6.0)	10 (20.0)	37 (74)	1 (2)	12 (24.5)	36 (73.5)	1 (33.3)	2 (66.7)	0 (0)	0 (0)	12 (26.1)	34 (73.9)
Cabbage	No Difference	4 (3.4)	41 (35.0)	72 (61.5)	4 (3.2)	32 (25.8)	88 (71)	4 (33.3)	7 (58.3)	1 (8.3)	2 (1.8)	26 (23.9)	81 (74.3)
Cabbage	Beneficial	0 (0)	1 (16.7)	5 (83.3)	0 (0)	2 (28.6)	5 (71.4)	0 (0)	1 (100)	0 (0)	0 (0)	0 (0)	7 (100)
Cabbage	Adverse	4 (3.3)	23 (18.9)	95 (77.9)	4 (3.2)	37 (29.8)	83 (66.9)	1 (14.3)	4 (57.1)	2 (28.6)	2 (1.8)	29 (26.6)	78 (71.6)
Cauliflower	No Difference	3 (1.8)	47 (28.3)	116 (69.9)	6 (3.4)	48 (27)	124 (69.7)	3 (42.9)	3 (42.9)	1 (14.3)	3 (2)	39 (25.5)	111 (72.6)
Cauliflower	Beneficial	1 (5.6)	4 (22.2)	13 (72.2)	0 (0)	7 (36.8)	12 (63.2)	0 (0)	0 (0)	0 (0)	0 (0)	2 (11.1)	16 (88.9)
Cauliflower	Adverse	3 (4.8)	14 (22.6)	45 (72.6)	2 (3.3)	18 (30.0)	40 (66.7)	1 (25.0)	2 (50.0)	1 (25.0)	1 (1.8)	14 (25.0)	41 (73.2)
Chinese Greens	No Difference	2 (1.9)	35 (32.7)	70 (65.4)	4 (3.6)	24 (21.4)	84 (75.0)	4 (33.3)	7 (58.3)	1 (8.3)	1 (1)	29 (29.9)	67 (69.1)
Chinese Greens	Beneficial	1 (11.1)	2 (22.2)	6 (66.7)	0 (0)	3 (37.5)	5 (62.5)	0 (0)	0 (0)	0 (0)	0 (0)	0 (0)	8 (100)
Chinese Greens	Adverse	1 (3.2)	8 (25.8)	22 (71)	1 (3.1)	10 (31.3)	21 (65.6)	0 (0)	1 (50.0)	1 (50.0)	0 (0)	11 (34.4)	21 (65.6)
Rocket	No Difference	4 (2.7)	46 (30.9)	99 (66.4)	5 (3.3)	37 (24.2)	111 (72.6)	3 (21.4)	9 (64.3)	2 (14.3)	5 (3.8)	34 (25.8)	93 (70.5)
Rocket	Beneficial	0 (0)	2 (40.0)	3 (60.0)	0 (0)	2 (40.0)	3 (60.0)	0 (0)	0 (0)	0 (0)	0 (0)	0 (0)	5 (100)
Rocket	Adverse	0 (0)	4 (17.4)	19 (82.6)	0 (0)	7 (29.2)	17 (70.8)	0 (0)	1 (50.0)	1 (50.0)	0 (0)	9 (39.1)	14 (60.9)
Watercress	No Difference	2 (2.9)	20 (29.4)	46 (67.7)	2 (2.7)	17 (23.3)	54 (74)	1 (20.0)	4 (80.0)	0 (0)	0 (0)	15 (23.1)	50 (76.9)

Table S1. *Cont.*

		**TLR9**	**rs5743836**		**TNFALPHA**	**rs1800629**		**TNFRSF1B**	**rs3397**		**TNFSF15**	**rs10982412**	
		C/C	C/T	T/T	A/A	A/G	G/G	C/C	C/T	T/T	A/A	A/G	G/G
Watercress	Beneficial	1 (12.5)	1 (12.5)	6 (75.0)	0 (0)	3 (33.3)	6 (66.7)	1 (50.0)	0 (0)	1 (50.0)	0 (0)	1 (11.1)	8 (88.9)
Watercress	Adverse	1 (5.9)	2 (11.8)	14 (82.4)	0 (0)	5 (31.3)	11 (68.8)	1 (50.0)	1 (50.0)	0 (0)	0 (0)	6 (37.5)	10 (62.5)
Horseradish	No Difference	1 (1.2)	28 (32.9)	56 (65.9)	3 (3.4)	20 (22.7)	65 (73.9)	2 (40.0)	3 (60.0)	0 (0)	1 (1.3)	20 (26)	56 (72.7)
Horseradish	Beneficial	0 (0)	0 (0)	2 (100)	0 (0)	0 (0)	1 (100)	0 (0)	0 (0)	0 (0)	0 (0)	0 (0)	2 (100)
Horseradish	Adverse	2 (4.4)	14 (30.4)	30 (65.2)	1 (2.1)	13 (27.7)	33 (70.2)	2 (28.6)	4 (57.1)	1 (14.3)	0 (0)	14 (29.8)	33 (70.2)
Mustard powder	No Difference	2 (1.3)	49 (31.8)	103 (66.9)	4 (2.5)	37 (22.8)	121 (74.7)	4 (28.6)	8 (57.1)	2 (14.3)	4 (2.9)	36 (26.1)	98 (71)
Mustard powder	Beneficial	0 (0)	1 (33.3)	2 (66.7)	0 (0)	2 (66.7)	1 (33.3)	0 (0)	0 (0)	0 (0)	0 (0)	0 (0)	3 (100)
Mustard powder	Adverse	3 (5.9)	11 (21.6)	37 (72.6)	3 (5.9)	14 (27.5)	34 (66.7)	1 (25.0)	2 (50.0)	1 (25.0)	0 (0)	14 (28.6)	35 (71.4)
Mustard sauce	No Difference	2 (1.4)	44 (30.6)	98 (68.1)	4 (2.7)	34 (22.5)	113 (74.8)	3 (27.3)	7 (63.6)	1 (9.1)	3 (2.3)	34 (25.6)	96 (72.2)
Mustard sauce	Beneficial	0 (0)	1 (33.3)	2 (66.7)	0 (0)	0 (0)	4 (100)	0 (0)	0 (0)	1 (100)	0 (0)	0 (0)	4 (100)
Mustard sauce	Adverse	3 (4.7)	13 (20.3)	48 (75.0)	2 (3.2)	17 (27.4)	43 (69.4)	2 (33.3)	4 (66.7)	0 (0)	0 (0)	12 (20.0)	48 (80.0)
Wasabi	No Difference	2 (2.6)	24 (31.6)	50 (65.8)	4 (5)	14 (17.5)	62 (77.5)	1 (25.0)	3 (75.0)	0 (0)	1 (1.5)	14 (20.3)	54 (78.3)
Wasabi	Beneficial	0 (0)	0 (0)	3 (100)	0 (0)	0 (0)	3 (100)	0 (0)	0 (0)	0 (0)	0 (0)	1 (33.3)	2 (66.7)
Wasabi	Adverse	2 (3.6)	12 (21.4)	42 (75.0)	1 (1.8)	20 (35.1)	36 (63.2)	2 (25.0)	5 (62.5)	1 (12.5)	0 (0)	14 (25.0)	42 (75.0)

Table S1. *Cont.*

		**TNFSF15**	**rs3810936**		**TNFSF15**	**rs7029554**		**TNFSF15**	**rs7867918**		**TNRC6C**	**rs4362447**	
		C/C	C/T	T/T	A/A	A/G	G/G	G/G	G/T	T/T	C/C	C/T	T/T
Broccoli	No Difference	80 (44.0)	85 (46.7)	17 (9.3)	121 (65.8)	58 (31.5)	5 (2.7)	131 (70.8)	46 (24.9)	8 (4.3)	82 (46.6)	76 (43.2)	18 (10.2)
Broccoli	Beneficial	9 (42.9)	8 (38.1)	4 (19.1)	20 (90.9)	2 (9.1)	0 (0)	14 (63.6)	6 (27.3)	2 (9.1)	8 (38.1)	9 (42.9)	4 (19.1)
Broccoli	Adverse	24 (48.0)	22 (44.0)	4 (8.0)	34 (68)	16 (32)	0 (0)	36 (72)	14 (28.0)	0 (0)	16 (32.7)	28 (57.1)	5 (10.2)
Cabbage	No Difference	59 (48.0)	52 (42.3)	12 (9.8)	86 (68.8)	34 (27.2)	5 (4.0)	90 (72)	27 (21.6)	8 (6.4)	54 (44.6)	54 (44.6)	13 (10.7)
Cabbage	Beneficial	2 (28.6)	3 (42.9)	2 (28.6)	7 (100)	0 (0)	0 (0)	4 (57.1)	2 (28.6)	1 (14.3)	2 (28.6)	4 (57.1)	1 (14.3)
Cabbage	Adverse	52 (41.6)	61 (48.8)	12 (9.6)	85 (67.5)	40 (31.8)	1 (0.8)	89 (70.1)	36 (28.4)	2 (1.6)	55 (45.8)	51 (42.5)	14 (11.7)
Cauliflower	No Difference	76 (42.9)	83 (46.9)	18 (10.2)	121 (67.6)	54 (30.2)	4 (2.2)	130 (72.2)	42 (23.3)	8 (4.4)	81 (47.4)	75 (43.9)	15 (8.8)
Cauliflower	Beneficial	9 (50.0)	6 (33.3)	3 (16.7)	17 (89.5)	2 (10.5)	0 (0)	11 (57.9)	6 (31.6)	2 (10.5)	7 (36.8)	8 (42.1)	4 (21.1)
Cauliflower	Adverse	29 (46.8)	29 (46.8)	4 (6.5)	40 (64.5)	21 (33.9)	1 (1.6)	43 (69.4)	18 (29)	1 (1.6)	22 (36.7)	30 (50.0)	8 (13.3)
Chinese Greens	No Difference	51 (45.1)	49 (43.4)	13 (11.5)	74 (65.5)	35 (31)	4 (3.5)	79 (69.9)	29 (25.7)	5 (4.4)	49 (45.4)	45 (41.7)	14 (13)
Chinese Greens	Beneficial	4 (50.0)	2 (25.0)	2 (25.0)	7 (87.5)	1 (12.5)	0 (0)	4 (44.4)	3 (33.3)	2 (22.2)	3 (37.5)	2 (25.0)	3 (37.5)
Chinese Greens	Adverse	17 (51.5)	14 (42.4)	2 (6.1)	21 (63.6)	12 (36.4)	0 (0)	26 (78.8)	7 (21.2)	0 (0)	12 (36.4)	16 (48.5)	5 (15.2)
Rocket	No Difference	71 (45.8)	68 (43.9)	16 (10.3)	102 (65.4)	49 (31.4)	5 (3.2)	110 (70.1)	38 (24.2)	9 (5.7)	63 (42.3)	66 (44.3)	20 (13.4)
Rocket	Beneficial	2 (40.0)	2 (40.0)	1 (20.0)	5 (100)	0 (0)	0 (0)	2 (40.0)	2 (40.0)	1 (20.0)	2 (40.0)	3 (60.0)	0 (0)
Rocket	Adverse	14 (60.9)	7 (30.4)	2 (8.7)	14 (58.3)	9 (37.5)	1 (4.2)	17 (70.8)	7 (29.2)	0 (0)	4 (18.2)	16 (72.7)	2 (9.1)
Watercress	No Difference	34 (46.0)	30 (40.5)	10 (13.5)	55 (74.3)	18 (24.3)	1 (1.4)	52 (70.3)	17 (23.0)	5 (6.8)	35 (48.6)	27 (37.5)	10 (13.9)
Watercress	Beneficial	4 (44.4)	2 (22.2)	3 (33.3)	8 (88.9)	1 (11.1)	0 (0)	4 (44.4)	3 (33.3)	2 (22.2)	3 (33.3)	4 (44.4)	2 (22.2)

Table S1. *Cont.*

		**TNFSF15**	**rs3810936**		**TNFSF15**	**rs7029554**		**TNFSF15**	**rs7867918**		**TNRC6C**	**rs4362447**	
		C/C	C/T	T/T	A/A	A/G	G/G	G/G	G/T	T/T	C/C	C/T	T/T
Watercress	Adverse	10 (58.8)	7 (41.2)	0 (0)	9 (52.9)	7 (41.2)	1 (5.9)	12 (70.6)	5 (29.4)	0 (0)	6 (35.3)	7 (41.2)	4 (23.5)
Horseradish	No Difference	43 (47.8)	37 (41.1)	10 (11.1)	63 (70.0)	25 (27.8)	2 (2.2)	60 (66.7)	26 (28.9)	4 (4.4)	40 (45.5)	40 (45.5)	8 (9.1)
Horseradish	Beneficial	1 (100)	0 (0)	0 (0)	1 (100)	0 (0)	0 (0)	1 (50.0)	1 (50.0)	0 (0)	0 (0)	1 (100)	0 (0)
Horseradish	Adverse	21 (45.7)	22 (47.8)	3 (6.5)	32 (66.7)	16 (33.3)	0 (0)	34 (70.8)	13 (27.1)	1 (2.1)	19 (41.3)	20 (43.5)	7 (15.2)
Mustard powder	No Difference	80 (49.7)	67 (41.6)	14 (8.7)	105 (64.8)	50 (30.9)	7 (4.3)	111 (68.5)	44 (27.2)	7 (4.3)	70 (44.9)	70 (44.9)	16 (10.3)
Mustard powder	Beneficial	1 (33.3)	2 (66.7)	0 (0)	3 (100)	0 (0)	0 (0)	1 (33.3)	1 (33.3)	1 (33.3)	1 (33.3)	2 (66.7)	0 (0)
Mustard powder	Adverse	20 (39.2)	26 (51)	5 (9.8)	35 (66)	18 (34)	0 (0)	40 (74.1)	12 (22.2)	2 (3.7)	21 (40.4)	25 (48.1)	6 (11.5)
Mustard sauce	No Difference	75 (49.7)	61 (40.4)	15 (9.9)	97 (64.2)	48 (31.8)	6 (4.0)	105 (69.5)	39 (25.8)	7 (4.6)	67 (45)	65 (43.6)	17 (11.4)
Mustard sauce	Beneficial	3 (75.0)	1 (25.0)	0 (0)	4 (100)	0 (0)	0 (0)	1 (25.0)	3 (75.0)	0 (0)	2 (50.0)	2 (50.0)	0 (0)
Mustard sauce	Adverse	23 (37.1)	33 (53.2)	6 (9.7)	50 (76.9)	15 (23.1)	0 (0)	45 (68.2)	18 (27.3)	3 (4.6)	23 (37.1)	31 (50.0)	8 (12.9)
Wasabi	No Difference	37 (46.8)	34 (43)	8 (10.1)	56 (70.0)	23 (28.8)	1 (1.3)	53 (66.3)	24 (30.0)	3 (3.8)	37 (47.4)	34 (43.6)	7 (9)
Wasabi	Beneficial	2 (66.7)	1 (33.3)	0 (0)	2 (66.7)	1 (33.3)	0 (0)	2 (66.7)	1 (33.3)	0 (0)	1 (33.3)	2 (66.7)	0 (0)
Wasabi	Adverse	27 (46.6)	22 (37.9)	9 (15.5)	43 (71.7)	15 (25.0)	2 (3.3)	42 (70.0)	15 (25.0)	3 (5)	24 (42.1)	24 (42.1)	9 (15.8)

Table S1. *Cont.*

		TRAIP	rs10865959		TRAIP	rs17598137		TRAIP	rs2271960		TRAIP	rs6446298	
		C/C	C/G	G/G	A/A	A/C	C/C	C/C	C/T	T/T	C/C	C/T	T/T
Broccoli	No Difference	0 (0)	107 (57.8)	78 (42.2)	1 (0.6)	25 (14.3)	149 (85.1)	36 (19.8)	99 (54.4)	47 (25.8)	87 (47.5)	81 (44.3)	15 (8.2)
Broccoli	Beneficial	0 (0)	10 (45.5)	12 (54.6)	0 (0)	2 (10.0)	18 (90.0)	3 (15)	8 (40.0)	9 (45)	6 (27.3)	12 (54.6)	4 (18.2)
Broccoli	Adverse	0 (0)	22 (42.3)	30 (57.7)	1 (2)	10 (19.6)	40 (78.4)	8 (15.7)	26 (51)	17 (33.3)	23 (44.2)	19 (36.5)	10 (19.2)
Cabbage	No Difference	0 (0)	75 (59.5)	51 (40.5)	1 (0.8)	19 (15.6)	102 (83.6)	27 (21.4)	65 (51.6)	34 (27)	62 (49.6)	52 (41.6)	11 (8.8)
Cabbage	Beneficial	0 (0)	5 (71.4)	2 (28.6)	0 (0)	0 (0)	7 (100)	2 (28.6)	3 (42.9)	2 (28.6)	3 (42.9)	4 (57.1)	0 (0)
Cabbage	Adverse	0 (0)	65 (50.8)	63 (49.2)	1 (0.8)	18 (15)	101 (84.2)	22 (17.7)	67 (54)	35 (28.2)	54 (42.5)	60 (47.2)	13 (10.2)
Cauliflower	No Difference	0 (0)	106 (58.9)	74 (41.1)	1 (0.6)	23 (13.5)	147 (86)	35 (19.7)	96 (53.9)	47 (26.4)	84 (47.2)	78 (43.8)	16 (9)
Cauliflower	Beneficial	0 (0)	10 (52.6)	9 (47.4)	0 (0)	2 (11.8)	15 (88.2)	3 (17.7)	8 (47.1)	6 (35.3)	5 (26.3)	11 (57.9)	3 (15.8)
Cauliflower	Adverse	0 (0)	25 (39.1)	39 (60.9)	1 (1.6)	12 (19.7)	48 (78.7)	12 (19.4)	28 (45.2)	22 (35.5)	30 (46.9)	24 (37.5)	10 (15.6)
Chinese Greens	No Difference	0 (0)	70 (62)	43 (38.1)	1 (0.9)	17 (15.6)	91 (83.5)	30 (26.8)	54 (48.2)	28 (25.0)	56 (49.6)	48 (42.5)	9 (8.0)
Chinese Greens	Beneficial	0 (0)	4 (44.4)	5 (55.6)	0 (0)	0 (0)	8 (100)	0 (0)	5 (62.5)	3 (37.5)	1 (11.1)	7 (77.8)	1 (11.1)
Chinese Greens	Adverse	0 (0)	19 (55.9)	15 (44.1)	0 (0)	5 (15.2)	28 (84.9)	6 (17.7)	18 (52.9)	10 (29.4)	16 (47.1)	14 (41.2)	4 (11.8)
Rocket	No Difference	0 (0)	92 (57.9)	67 (42.1)	2 (1.3)	23 (15.1)	127 (83.6)	31 (19.9)	87 (55.8)	38 (24.4)	74 (46.8)	71 (44.9)	13 (8.2)
Rocket	Beneficial	0 (0)	3 (60.0)	2 (40.0)	0 (0)	0 (0)	5 (100)	1 (20.0)	2 (40.0)	2 (40.0)	1 (20.0)	3 (60.0)	1 (20.0)
Rocket	Adverse	0 (0)	9 (37.5)	15 (62.5)	0 (0)	4 (16.0)	21 (84)	2 (8.0)	14 (56.0)	9 (36)	10 (41.7)	12 (50.0)	2 (8.3)
Watercress	No Difference	0 (0)	43 (58.9)	30 (41.1)	1 (1.4)	9 (12.7)	61 (85.9)	15 (20.3)	44 (59.5)	15 (20.3)	33 (45.2)	33 (45.2)	7 (9.6)

Table S1. *Cont.*

		TRAIP	rs10865959		TRAIP	rs17598137		TRAIP	rs2271960		TRAIP	rs6446298	
		C/C	C/G	G/G	A/A	A/C	C/C	C/C	C/T	T/T	C/C	C/T	T/T
Watercress	Beneficial	0 (0)	6 (66.7)	3 (33.3)	0 (0)	0 (0)	8 (100)	1 (12.5)	5 (62.5)	2 (25.0)	1 (11.1)	7 (77.8)	1 (11.1)
Watercress	Adverse	0 (0)	7 (41.2)	10 (58.8)	0 (0)	5 (29.4)	12 (70.6)	3 (17.7)	8 (47.1)	6 (35.3)	7 (41.2)	8 (47.1)	2 (11.8)
Horseradish	No Difference	0 (0)	53 (58.9)	37 (41.1)	1 (1.2)	10 (11.5)	76 (87.4)	16 (18)	49 (55.1)	24 (27)	37 (41.6)	42 (47.2)	10 (11.2)
Horseradish	Beneficial	0 (0)	1 (50.0)	1 (50.0)	0 (0)	0 (0)	2 (100)	1 (50.0)	0 (0)	1 (50.0)	1 (50.0)	0 (0)	1 (50.0)
Horseradish	Adverse	0 (0)	26 (54.2)	22 (45.8)	1 (2.2)	7 (15.2)	38 (82.6)	10 (21.3)	27 (57.5)	10 (21.3)	17 (35.4)	28 (58.3)	3 (6.3)
Mustard powder	No Difference	0 (0)	87 (54)	74 (46.0)	1 (0.6)	22 (14.1)	133 (85.3)	30 (18.8)	86 (53.8)	44 (27.5)	74 (46.3)	70 (43.8)	16 (10.0)
Mustard powder	Beneficial	0 (0)	3 (100)	0 (0)	0 (0)	0 (0)	3 (100)	1 (33.3)	2 (66.7)	0 (0)	1 (33.3)	2 (66.7)	0 (0)
Mustard powder	Adverse	0 (0)	31 (54.4)	26 (45.6)	1 (1.9)	8 (14.8)	45 (83.3)	10 (18.2)	30 (54.6)	15 (27.3)	23 (40.4)	27 (47.4)	7 (12.3)
Mustard sauce	No Difference	0 (0)	83 (55.0)	68 (45)	0 (0)	19 (13.2)	125 (86.8)	26 (17.5)	79 (53)	44 (29.5)	66 (43.7)	69 (45.7)	16 (10.6)
Mustard sauce	Beneficial	0 (0)	2 (50.0)	2 (50.0)	1 (25.0)	0 (0)	3 (75.0)	3 (75.0)	0 (0)	1 (25.0)	3 (75.0)	1 (25.0)	0 (0)
Mustard sauce	Adverse	0 (0)	36 (54.6)	30 (45.5)	1 (1.6)	9 (14.1)	54 (84.4)	14 (21.5)	32 (49.2)	19 (29.2)	27 (41.5)	29 (44.6)	9 (13.9)
Wasabi	No Difference	0 (0)	49 (62)	30 (38.0)	0 (0)	10 (12.8)	68 (87.2)	16 (20.5)	43 (55.1)	19 (24.4)	37 (46.8)	37 (46.8)	5 (6.3)
Wasabi	Beneficial	0 (0)	2 (66.7)	1 (33.3)	1 (33.3)	0 (0)	2 (66.7)	2 (66.7)	1 (33.3)	0 (0)	2 (66.7)	1 (33.3)	0 (0)
Wasabi	Adverse	0 (0)	27 (45)	33 (55.0)	1 (1.8)	10 (17.5)	46 (80.7)	12 (20.0)	30 (50.0)	18 (30.0)	23 (38.3)	30 (50.0)	7 (11.7)

Table S1. *Cont.*

		**USP4**	**rs1865741**		**USP4**	**rs9881860**		**VDR**	**rs7975232**	
		C/C	C/T	T/T	A/A	A/T	T/T	A/A	A/C	C/C
Broccoli	No Difference	59 (32.1)	94 (51.1)	31 (16.9)	4 (2.3)	62 (34.8)	112 (62.9)	55 (30.6)	97 (53.9)	28 (15.6)
Broccoli	Beneficial	7 (31.8)	12 (54.6)	3 (13.6)	0 (0)	8 (36.4)	14 (63.6)	8 (36.4)	7 (31.8)	7 (31.8)
Broccoli	Adverse	15 (28.9)	22 (42.3)	15 (28.9)	5 (9.8)	17 (33.3)	29 (56.9)	15 (30.0)	25 (50.0)	10 (20.0)
Cabbage	No Difference	39 (31)	69 (54.8)	18 (14.3)	3 (2.4)	42 (34.2)	78 (63.4)	37 (30.3)	67 (54.9)	18 (14.8)
Cabbage	Beneficial	4 (57.1)	2 (28.6)	1 (14.3)	0 (0)	1 (14.3)	6 (85.7)	3 (42.9)	3 (42.9)	1 (14.3)
Cabbage	Adverse	40 (31.5)	59 (46.5)	28 (22.1)	5 (4.1)	43 (35.0)	75 (61)	38 (30.7)	59 (47.6)	27 (21.8)
Cauliflower	No Difference	59 (33.0)	90 (50.3)	30 (16.8)	4 (2.3)	59 (33.9)	111 (63.8)	54 (30.9)	91 (52.0)	30 (17.1)
Cauliflower	Beneficial	6 (31.6)	11 (57.9)	2 (10.5)	0 (0)	8 (42.1)	11 (57.9)	8 (42.1)	6 (31.6)	5 (26.3)
Cauliflower	Adverse	19 (29.7)	27 (42.2)	18 (28.1)	5 (8.1)	20 (32.3)	37 (59.7)	20 (32.3)	33 (53.2)	9 (14.5)
Chinese Greens	No Difference	39 (34.5)	62 (54.9)	12 (10.6)	3 (2.8)	35 (32.1)	71 (65.1)	33 (31.1)	56 (52.8)	17 (16.0)
Chinese Greens	Beneficial	3 (33.3)	4 (44.4)	2 (22.2)	0 (0)	3 (37.5)	5 (62.5)	1 (11.1)	5 (55.6)	3 (33.3)
Chinese Greens	Adverse	10 (29.4)	14 (41.2)	10 (29.4)	3 (8.8)	11 (32.4)	20 (58.8)	13 (39.4)	13 (39.4)	7 (21.2)
Rocket	No Difference	45 (28.5)	87 (55.1)	26 (16.5)	4 (2.6)	57 (37.3)	92 (60.1)	52 (34.9)	71 (47.7)	26 (17.5)
Rocket	Beneficial	2 (40.0)	2 (40.0)	1 (20.0)	0 (0)	3 (60.0)	2 (40.0)	3 (60.0)	2 (40.0)	0 (0)
Rocket	Adverse	8 (33.3)	7 (29.2)	9 (37.5)	3 (12.5)	3 (12.5)	18 (75.0)	6 (24.0)	13 (52.0)	6 (24.0)
Watercress	No Difference	25 (34.3)	35 (48.0)	13 (17.8)	3 (4.2)	26 (36.1)	43 (59.7)	24 (34.3)	32 (45.7)	14 (20.0)
Watercress	Beneficial	3 (33.3)	6 (66.7)	0 (0)	0 (0)	4 (44.4)	5 (55.6)	4 (44.4)	4 (44.4)	1 (11.1)
Watercress	Adverse	4 (23.5)	9 (52.9)	4 (23.5)	1 (6.3)	6 (37.5)	9 (56.3)	4 (25.0)	8 (50.0)	4 (25.0)
Horseradish	No Difference	30 (33.3)	48 (53.3)	12 (13.3)	2 (2.3)	30 (34.5)	55 (63.2)	29 (34.1)	45 (52.9)	11 (12.9)
Horseradish	Beneficial	2 (100)	0 (0)	0 (0)	0 (0)	0 (0)	2 (100)	2 (100)	0 (0)	0 (0)
Horseradish	Adverse	16 (33.3)	21 (43.8)	11 (22.9)	4 (8.3)	19 (39.6)	25 (52.1)	9 (19.6)	25 (54.4)	12 (26.1)
Mustard powder	No Difference	48 (29.8)	84 (52.2)	29 (18)	7 (4.5)	52 (33.1)	98 (62.4)	47 (30.3)	84 (54.2)	24 (15.5)
Mustard powder	Beneficial	2 (66.7)	1 (33.3)	0 (0)	0 (0)	1 (33.3)	2 (66.7)	2 (66.7)	1 (33.3)	0 (0)
Mustard powder	Adverse	20 (35.7)	26 (46.4)	10 (17.9)	1 (1.8)	20 (35.7)	35 (62.5)	21 (38.2)	24 (43.6)	10 (18.2)
Mustard sauce	No Difference	49 (32.5)	79 (52.3)	23 (15.2)	6 (4.1)	42 (28.8)	98 (67.1)	42 (29)	80 (55.2)	23 (15.9)
Mustard sauce	Beneficial	1 (25.0)	1 (25.0)	2 (50.0)	1 (25.0)	2 (50.0)	1 (25.0)	3 (75.0)	1 (25.0)	0 (0)
Mustard sauce	Adverse	25 (38.5)	27 (41.5)	13 (20.0)	1 (1.5)	24 (36.9)	40 (61.5)	19 (28.4)	33 (49.3)	15 (22.4)
Wasabi	No Difference	21 (26.6)	46 (58.2)	12 (15.2)	2 (2.6)	29 (38.2)	45 (59.2)	25 (32.9)	37 (48.7)	14 (18.4)
Wasabi	Beneficial	2 (66.7)	0 (0)	1 (33.3)	1 (33.3)	0 (0)	2 (66.7)	3 (100)	0 (0)	0 (0)
Wasabi	Adverse	17 (28.8)	30 (50.9)	12 (20.3)	3 (5.1)	25 (42.4)	31 (52.5)	17 (29.3)	29 (50.0)	12 (20.7)
